# SubsidyExplorer: A decision-support tool to improve our understanding of the ecological and economic effects of reforming fisheries subsidies

**DOI:** 10.1371/journal.pone.0265829

**Published:** 2022-06-03

**Authors:** Katherine D. Millage, Vienna R. Saccomanno, Matthew M. Warham, Laura Lea Rubino, Anna Schuhbauer, U. Rashid Sumaila, Christopher Costello

**Affiliations:** 1 Bren School of Environmental Science and Management, University of California Santa Barbara, Santa Barbara, California, United States of America; 2 Marine Science Institute, University of California Santa Barbara, Santa Barbara, California, United States of America; 3 Institute for the Ocean and Fisheries, The University of British Columbia, Vancouver, British Columbia, Canada; 4 School of Public Policy and Global Affairs, The University of British Columbia, Vancouver, British Columbia, Canada; 5 Department of Economics, University of California Santa Barbara, Santa Barbara, California, United States of America; Maurice Lamontagne Institute, CANADA

## Abstract

The magnitude of subsidies provided to the fishing sector by governments worldwide is immense—an estimated $35.4 billion USD per year. The majority of these subsidies may be impeding efforts to sustainably manage fisheries by incentivizing overfishing and overcapacity. Recognizing the threat these subsidies pose, the World Trade Organization has set a goal of reaching an agreement that would end fisheries subsidies that contribute to overcapacity, overfishing, and illegal fishing. However, negotiations have been hampered by uncertainty around the likely effects of reforming these subsidies. Here we present a novel method for translating a bioeconomic model into an interactive online decision support tool that draws upon real-world data on fisheries subsidies and industrial fishing activity so users can directly compare the relative ambition levels of different subsidy reform options.

## Introduction

Global capture fisheries produce more than 90 million tonnes of fish per year and employ roughly 39 million people [1]. Given their social significance, it is unsurprising that governments highly subsidize this sector, with approximately $35.4 billion USD per year provided to marine fisheries [2]. A key defining characteristic of most fisheries subsidies is that they allow the fisheries sector to be more profitable than it would otherwise be [3]. Some subsidies support crucial functions such as fisheries management [4], but others—often termed “capacity-enhancing subsidies”—may have concerning effects. Capacity-enhancing subsidies artificially lower the costs or increase the revenues of fishing, thereby giving rise to overcapacity, which can lead to overfishing. The threat that capacity-enhancing subsidies pose to fisheries sustainability has long been understood [5], leading the United Nations (UN) to call for an end to certain fisheries subsidies that contribute to overcapacity, overfishing, and illegal fishing as part of Sustainable Development Goal (SDG) 14.6 [6].

The World Trade Organization (WTO) has been discussing fisheries subsidies reform for two decades. The latest round of reform negotiations began in 2017, at which time the WTO set a goal of adopting an agreement on fisheries subsidies that would deliver on SDG 14.6, ending the provisioning of harmful fisheries subsidies. Member delegations to the WTO began submitting subsidy reform proposals at the end of 2017. Proposed reform criteria focused primarily on three key categories: 1) subsidies to illegal, unreported, and unregulated (IUU) fishing, 2) subsidies to fishing on overfished stocks, and 3) subsidies contributing to overcapacity and overfishing.

While delegates from each WTO Member country (“negotiators”) have identified a set of potential reforms to consider, negotiations have been hampered by the unwillingness of some Members to take actions that might negatively affect their respective fishing sectors. Fisheries are a sensitive issue for many countries because of their socio-economic (e.g., employment, production of food, maintenance of traditional livelihoods) and geopolitical (e.g., avoidance of conflict, regional influence) importance [7, 8]. Uncertainty around which countries and fishing sectors would be affected by reforms and how big the effects would actually be has only exacerbated such insecurities. Given the interconnectedness of global fisheries and diversity of fishery subsidy programs across the world [9], the costs and benefits of reform will undoubtedly be differentially distributed across Member nations. Though removing subsidies may be simple in theory, the process of reaching an agreement is fraught with complications. Much to the chagrin of the larger WTO community, some Members have taken a strong position in these negotiations: they want to see the complete removal of all capacity-enhancing subsidies. This approach has received much opposition (and, realistically speaking, is unlikely to be an outcome of the negotiations) because of the near-term costs it could impose on currently subsidized fishers. Therefore, within each of the three aforementioned categories, Members have proposed a number of different potential rules related to subsidy provisioning—termed “disciplines”—that have served as the basis for discussions related to a potential agreement. The final agreement’s ultimate consequences will depend on the collection of specific disciplines included (the “reform package”).

Decision support tools are increasingly being used as powerful instruments for making the findings or methods of academic research more accessible to policy makers [10–12]. Here we present an interactive, web-based decision support application called SubsidyExplorer (http://www.subsidyexplorer.org/) designed to help overcome the information gaps that have persistently hampered WTO subsidy negotiations. A hands-on tool that allows WTO negotiators to explore the potential outcomes of their proposals, SubsidyExplorer was collaboratively developed by academic research groups, non-governmental organizations, and other participants in the WTO negotiations. This tool uses a bioeconomic model to translate subsidy reform scenarios into global fishery consequences. It gives users the ability to directly compare the relative ambition levels of different subsidy reform packages and explore and interact with fisheries subsidies data alongside other relevant fisheries metrics. Here we present the novel datasets and analyses undertaken to develop this tool and provide an overview of the SubsidyExplorer platform. We then provide an overview of the results contained within SubsidyExplorer, provide illustrative examples that demonstrate the utility of the tool to negotiators, and conclude with key insights that could support ambitious subsidy reform at the WTO.

### Negotiating positions and policy context in 2021 leading to development of SubsidyExplorer

In May 2021, the Chair of the WTO fisheries subsidies negotiations released a draft text of a potential agreement, the first such text to be made publicly available since an earlier round of negotiations in 2007. This draft negotiating text reveals many of the key sticking points that have plagued WTO negotiations regarding subsidy reform for decades. Many of these issues may become obsolete as negotiations continue into 2022 and Members find ways to reach consensus. Nonetheless, we provide the following brief overview of key negotiating positions and points of contention in 2021 because they provide relevant historical context for the development of the SubsidyExplorer tool.

At its core, the 2021 draft text is a collection of compromises put together by the Chair in an effort to find common ground and strike a balance between the numerous positions voiced by Members between 2017–2021. Throughout the course of this round of WTO negotiations, one such source of common ground has been the inclusion of disciplines for subsidies to IUU fishing. Most Members agree with the idea of prohibiting subsidies to a vessel (or operator of a vessel) when they have been found to be engaged in IUU fishing. The main source of debate on the topic centers around who gets to make such a determination, how quickly it would result in subsidies being removed (“the prohibition”), and for how long that prohibition might last.

The second category of potential disciplines—subsidies to fishing on overfished stocks—has been more contentious, but the idea is to prohibit subsidies to fishing on stocks that are already overfished. Some Members, including Australia, New Zealand, and the United States, have pushed for the inclusion of scientifically-based targets (e.g., current biomass relative to that which would produce the maximum sustainable yield, B/B_MSY_), but many others have pushed back, citing a lack of resources available to conduct such assessments. A compromise has been suggested that would give coastal states and Regional Fisheries Management Organizations (RFMOs) the freedom to make their own determinations regarding stocks that are overfished within their areas of jurisdiction and require all to use the best scientific evidence available to them to make these determinations.

The final category of reforms addresses what many Members consider to be the most important goal: removing subsidies that contribute to overcapacity and overfishing. There has been much disagreement throughout the negotiations on how such disciplines should be crafted (e.g., a list of prohibited subsidies vs. a cap and tier approach). Many Members, including China, Brazil, Indonesia, and the United States, have put forth cap-based approaches, but consensus is now building around a list-based approach. A key challenge that remains is achieving alignment on the types of subsidies to be prohibited under this discipline and balancing this with an exemption, which has been supported by the EU, for Members that can demonstrate the existence of measures that would protect against negative subsidy effects. Specific rules related to fishing that happens outside the jurisdiction of any nation (“on the high seas”) also remain a sticking point, but undoubtedly, the largest point of contention relates to the inclusion of special and differential treatment (S&DT) for developing country Members. S&DT is a politically sensitive topic that could have implications for disciplines across all three categories. It has led multiple coalitions of Members, including the Least Developed Countries (LDC) group, the African, Caribbean, and Pacific (ACP) group, and a collection of South Asian countries led by India, to put forth proposals that address this topic. The balances that are ultimately struck on these issues will largely define the impact of a potential agreement.

## Materials and methods

**Fig 1:**
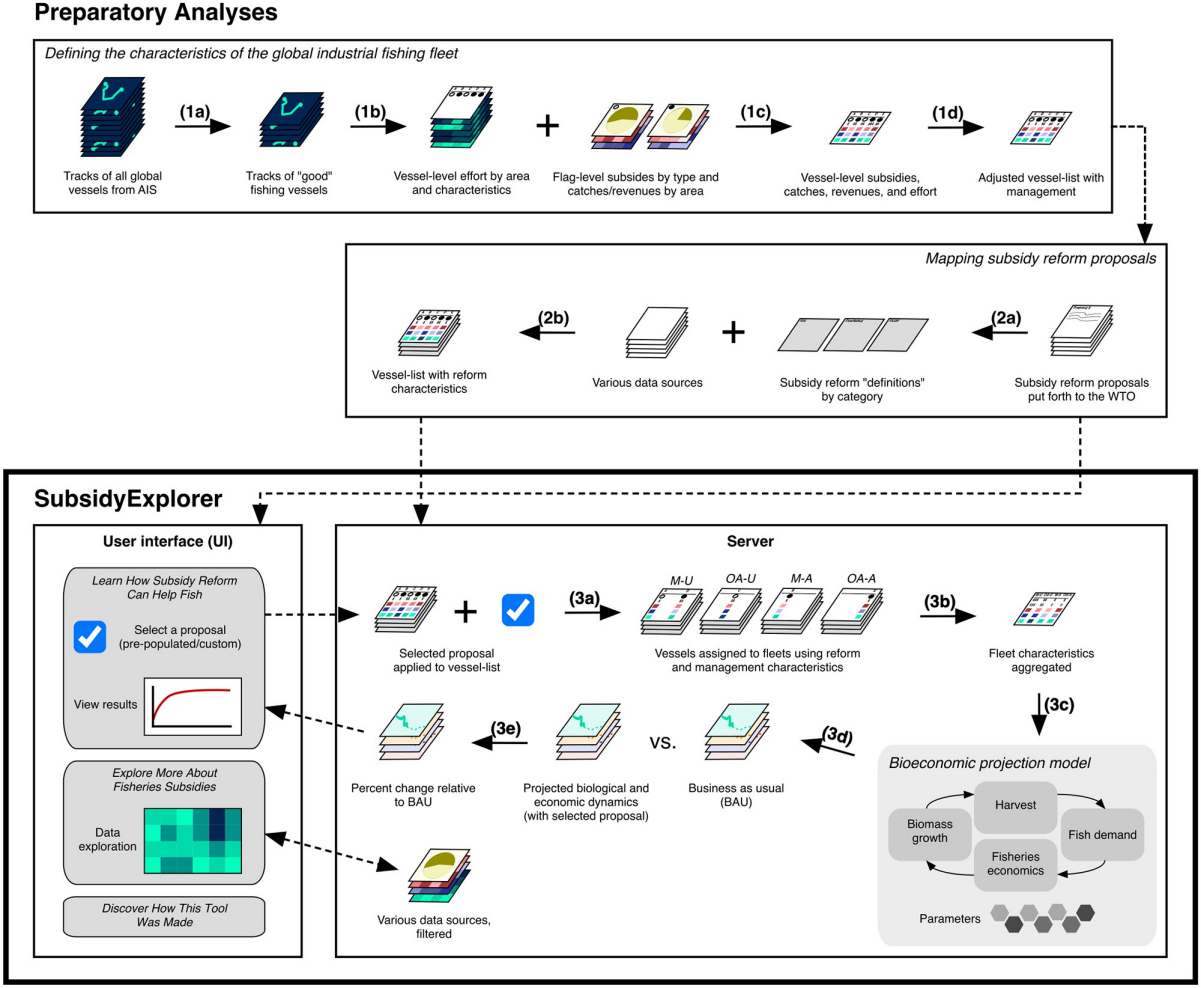
Conceptual schematic of the data, analyses, and processes underlying the SubsidyExplorer toolkit. The first box (steps 1a—1d) illustrates the process of combining data sources to create a dataset that defines the activities of the global industrial fishing fleet—described in the text in subsection “Defining the characteristics of the global industrial fishing fleet”. The second box (steps 2a—2c) represents the process of mapping subsidy reform proposals submitted to the WTO on to the global vessel database—to allow for their effects to later be modeled—and is described in the text in subsection “Modeling subsidy reform proposals”. The products created in the first two series of steps feed into SubsidyExplorer, but the component analyses are not contained within the toolkit itself. The final two boxes contained within the bolded outline represent the user interface and server of the SubsidyExplorer tool, and the happenings that occur therein. Steps 3a—3d illustrate the process of running the model and generating results when a user selects a subsidy reform proposal—described in the text in subsections “Bioeconomic projection model” and “Characterization of fishing fleets and their behavior”.

SubsidyExplorer uses the following general decision-making framework to compare policies: Step 1) Choose among different subsidy reform proposals submitted to the WTO by different delegations or design a new subsidy reform proposal by selecting from individual disciplines; Step 2) Learn about the chosen proposal and any existing data limitations, make assumptions to overcome these limitations if necessary; Step 3) Run the bioeconomic model; Step 4) Compare the effects of the chosen proposal on different biological and economic indicators and assess regional breakdown of impacts. Here we describe the data, preparatory analyses, and processes underlying the SubsidyExplorer toolkit that support this framework (Fig 1). First, we explain how we collated information from different sources—including near real-time positional data collected from satellites—to build a global database of industrial fishing activity. Second, we outline the process of breaking down and mapping potential WTO subsidy reform proposals onto this database. Third, we describe the bioeconomic projection model used to evaluate and compare the different policies and explain the process of delimiting fishing fleets that takes place within SubsidyExplorer. We conclude with a brief description of the packages used to develop the toolkit.

### Defining the characteristics of the global industrial fishing fleet

One foundational analysis that we performed to develop SubsidyExplorer involved combining data from many different sources to create a dataset that defines the activities of the global industrial fishing fleet. This novel analysis leverages satellite-derived estimates of global fishing effort from Global Fishing Watch (GFW) [13, 14], an aggregated version of which is freely available to download at https://globalfishingwatch.org/. There are very few estimates of total fishing effort at a global scale, but GFW offers a novel mechanism for tracking fishing behavior in near real time at the individual vessel level [14]. GWF data serves as the foundation of our global industrial fishing database, and the use of this novel dataset allowed us to collect high-resolution data on individual vessels’ behavior and characteristics. More information on the GFW dataset is included in the supporting information (section 1.4 of S1 Appendix).

The raw data used to create the GFW dataset may include transmissions from anything in the ocean with an AIS transponder. Therefore, to curate a trusted list of fishing vessels we take several steps to identify and extract data only originating from sources that are confirmed to be fishing vessels: 1) we remove all transmissions from objects that are likely fishing gear (buoys, nets, etc.), rather than vessels; 2) we apply a number of filters to remove transmissions associated with non-fishing and inactive vessels, as well as vessels that may be intentionally misrepresenting (“spoofing”) their positions; and 3) we manually remove certain vessels identified as non-fishing vessels (Fig 1, 1a). The specific criteria used to identify “good” fishing vessels is detailed in section 2.3.1 of S1 Appendix.

Once we create our list of fishing vessels, we then extract the number of hours that each vessel spent fishing in every Exclusive Economic Zone (EEZ) and on the high seas (areas of the ocean beyond the jurisdiction of any country) in a given year (Fig 1, 1b). We also extract eight characteristics for each vessel: flag state, vessel class, total length (m), gross tonnage (gt), engine power (kW), ship name, call sign, and registration number assigned by The International Maritime Organization (IMO). We then calculate fishing effort in units of fishing kilowatt-hours (kWh) by weighting the hours spent fishing by the engine power of the vessel. Expressing fishing effort in kWh (as opposed to just hours) gives us a better metric for comparing fishing effort across vessels with different gear types and/or sizes [14–17]. In total, the SubsidyExplorer toolkit’s GFW-derived database of fishing effort (for 2018) and vessel characteristics includes information from 70,586 unique vessels (Figs B and C in S1 Appendix).

We then estimate the catches, revenue, and subsidies associated with each vessel in our database using global landings data from the Food and Agriculture Organization of the UN (FAO) Global Capture Production Database [18], ex-vessel fish prices [19], and estimates of global fisheries subsidies being provided to industrial fisheries worldwide [20] (Fig 1, 1c). To estimate the landed value (“revenue”) of all species reported in the FAO Global Capture Production Database [18], we match ex-vessel prices [19] extrapolated to 2018 USD to capture production (“catch”) in 2018. We then calculate catch, revenue, and subsidy rates by flag state and FAO region based on the total observed fishing effort in each area from GFW. These rates—expressed in terms of tonnes/kWh, USD/kWh, and USD/kWh respectively—are then used to proportionally allocate catches, estimated landed value, and subsidies, based on the total fishing effort expended (in kWh) by each vessel [15]. Catches and estimated landed values are allocated by flag state and FAO region, while subsidies are allocated only by flag state. For example, if a single flag state provided $2 million USD in subsidies in 2018 to its vessels, and the total fishing effort by those vessels amounted to 15 million kWh, we would calculate a subsidy rate of $0.133 USD/kWh for that flag state. The calculation of catch and revenue rates differ slightly because we consider both flag state and FAO region. Imagine that vessels flagged to the aforementioned state spent 5 million kWh fishing in a single FAO region, and caught a total of 0.5 million tonnes of fish, with a landed value of $1 million USD in that region. We would calculate catch and revenue rates of 0.1 tonnes/kWh and $0.2 USD/kWh respectively for vessels flagged to that state fishing in that FAO region. More information on the process of combining these datasets can be found in section 2.3.3 of S1 Appendix.

Next, we convert the values of subsidies estimated for each vessel to “effective subsidies,” to account for the fact that one dollar of one type of subsidy may not have the same effect on fishing effort as one dollar of another type of subsidy [9]. The Organisation for Economic Co-operation and Development (OECD) has done extensive research on the relative effects of different types of fisheries subsidies on fishing effort, harvests, fleet capacity, fishers’ income, and stock size [9, 21]. We leverage these findings to calculate the “effective” (normalized) monetary values of different subsidy types as they relate to fishing effort (Tables H and I in S1 Appendix).

Finally, the causal relationship between certain types of fishery subsidies and overfishing (or overcapacity) likely depends on the effectiveness of the management system in place in the fishery. For example, subsidies that reduce fishing costs are less likely to have an effect in fisheries managed with quotas as compared to those managed with input restrictions (e.g., gear and vessel size restrictions) [22]. We therefore assign management scores [23] to each of our vessels based on the areas where they fish (Figs D and E in S1 Appendix). This allows us to later classify vessels into different management tiers that respond differently to subsidy reform (Fig 1, 1d).

### Modeling subsidy reform proposals

Short descriptions of all subsidy reform proposals being considered by WTO negotiators (and represented in SubsidyExplorer) are included in section 4.3 of S1 Appendix, as are the specific assumptions we made to model each text. To capture the reality that each proposal—or set of disciplines—would impact fishing vessels and fleets differently, we developed a method for linking the proposals to our global vessel database based on vessel characteristics and behavior. Here we briefly describe the process of creating additional characteristics to allow for the identification of vessels in the SubsidyExplorer toolkit that would be likely to be affected by different disciplines, resulting in removal of their respective subsidies.

These additional characteristics are based on our interpretation(s) of how existing data could be leveraged to identify prohibited subsidies based on a particular discipline (“definitions”) (Fig 1, 2a). For some proposed disciplines, we came up with more than one possible interpretation, or identified multiple data sources that could be used. We therefore allow for the possibility that different definitions could be used to isolate the effects of the same potential discipline depending on one’s interpretation. Some of the additional characteristics are binary (i.e., does a certain condition apply—yes or no?). Others have a discrete number of possible options (e.g., development status), and some have a range of possible values (e.g., the proportion of time spent fishing on the high seas) (Fig 1, 2b). Some characteristics therefore allow for alternative thresholds concerning a proposed discipline to be explored within the SubsidyExplorer toolkit.

The additional characteristics that we applied to our global vessel database include the flag-state’s development status, the fractions of effort spent fishing in areas beyond national jurisdiction (“high seas”), in the EEZs of other nations (“distant water”), and in territorial waters, and whether the vessel fished in disputed areas. Additionally, we identified whether each vessel was listed as having engaged in IUU fishing and determined the weighted mean stock status(es) in the area(s) in which each vessel fished [24, 25]. More information on the definitions we applied to different disciplines, and the additional vessel characteristics we created to apply subsidy reform proposals to our global vessel database is available in section 4.1 of S1 Appendix.

Some subsidy reform proposals suggest an alternative cap and tier approach to limiting subsidies, requiring us to also define country-level characteristics. Under a cap-based approach, WTO Members would be divided into tiers based on some country-level criteria, and then the total amount of permissible subsidies for Members in each tier would be capped based on other criteria or according to an agreed upon rule. For example, a potential capping rule could be to limit fisheries subsidies to a predetermined percentage of current levels. However, different tiers of Members could be established, perhaps defined by development status, such that countries in one tier (e.g., developed countries) were subject to a lower capping percentage (e.g., 3%), while those in another tier (e.g., developing countries) were allowed a higher percentage (e.g., 5%). The additional characteristics that we created for each country to model such proposals (section 4.2 of S1 Appendix) include total subsidy provisioning to the industrial fishing fleet by type [2, 20], marine capture production [18], landed value of marine capture production [18, 19], and subsidy dollars provided per fisher [2, 20, 26].

### Bioeconomic projection model

The structural bioeconomic model that underlies the SubsidyExplorer toolkit predicts how a global aggregate fishery would change under different subsidy reform policies. The simple biological model forecasts how global fish biomass will change based on the way that the selected subsidy reform policy affects the fishing mortality rate. This is determined by the characteristics of the respective portions of the global fishing fleet likely to be affected or unaffected by that reform. An economic model then calculates profits based on the fish price resulting from total harvest each year.

The model underpinning the SubsidyExplorer toolkit is similar to those used in the “Sunken Billions” reports [27, 28], which estimate rent losses for the global marine fishery. Our model was also inspired by, and shares many similarities with, bioeconomic models used in other academic fisheries works [e.g., 24, 29–31]. Though our model—as do many of the models from which ours draws inspiration—incorporates demand-side factors (to a limited extent), it primarily focuses on production-side dynamics and is not intended to predict market outcomes. Nonetheless, this model allows for the biological and economic performance of the global fishery to be assessed under different simulations in a transparent and comparable manner. This type of model is not intended to analyze the performance of individual fisheries. Indeed, one of the key advantages of this type of model is its simplicity—by keeping the number of parameters that must be estimated to a manageable number, we can keep the assumptions that need to be made to estimate those parameters to a minimum while still using robust empirical data on global fisheries. Descriptions of the models that inspired ours are provided in section 3.1 of S1 Appendix alongside a brief discussion of their key similarities and differences to the model underlying the SubsidyExplorer tool.

There are four main modules in the model: biomass growth, harvest, global fish demand, and fleet response. The component functions of these modules are discussed in more detail in the following sections.

#### Biomass growth

For our biomass growth module, we use a form of the basic logistic surplus production model commonly referred to as the Pella-Tomlinson Model [32]. With discrete units of time (hereafter denoted with the subscript *t* and considered to be years for the purposes of our model), biomass in the next year (*b*_*t+1*_), is described by:

$$b_{t+1}=b_{t}+\;\frac{\phi +\;1}{\phi }gb_{t}(1-(\frac{b_{t}}{K}{)}^{\phi })\;-\;h_{t}$$ (1)

where *b*_*t*_ is biomass in the current year, *φ* is a scalar parameter that allows for asymmetry in the production curve, *g* is the population growth rate for the global stock, *K* is the carrying capacity for the global stock (maximum population size allowable for growth to be positive), and *h*_*t*_ is the total harvest across all fleets in the current year.

The population growth rate of the stock (*g*) can be estimated directly from maximum sustainable yield (*MSY*), *K*, and *φ* as follows:

$$g=\frac{MSY}{K}(\phi +1{)}^{\frac{1}{\phi }}$$ (2)

#### Harvest

Our harvest module is made up of the sum of the individual harvest functions for each of the four fishing fleets. The creation of these fleets is detailed in the next section. Harvest for fleet *j* in the current year is represented by:

$$h_{j,t}=q_{j}b_{t}e_{j,t}$$ (3)

where *q*_*j*_ is a fleet-specific catchability parameter (time-invariant), *b*_*t*_ is the total global stock biomass in the current year, and *e*_*j*,*t*_ is the fishing effort of fleet *j* in the current year.

We estimate *q*_*j*_ directly for each fleet as follows:

$$q_{j}=\frac{h_{j,\;0}}{b_{0}*\;e_{j,0}}$$ (4)

where *h*_*j*,*0*_ is the harvest of fleet *j* in the base year, *b*_*0*_ is the total global stock biomass in the base year, and *e*_*j*,*0*_ is the fishing effort of fleet *j* in the base year. When the SubsidyExplorer toolkit was first developed and shared with WTO negotiators, 2018 was the most recent year for which complete catch and effort data were available globally and is therefore used as the base year for all simulations.

Total harvest in the current year is the sum of the harvests from all four fleets:

$$h_{t}=\sum_{i=1}^{j}h_{j,t}$$ (5)

#### Global fish demand

In order to allow the price of fish to change as a function of harvest, we introduce a demand function for fish, and we assume it is downward-sloping. Total harvest in a given year gives rise to fish price through the constant elasticity of demand function:

$$p_{t}=(\frac{1}{\delta }{)}^{\frac{1}{\epsilon }}{h_{t}}^{\frac{1}{\epsilon }}$$ (6)

where *ε* is the constant elasticity of demand, *δ* is a constant, and *h*_*t*_ is the total harvest across all fleets in the current year. We calculate *δ* as follows:

$$\delta =\frac{h_{0}}{{p_{0}}^{\epsilon }}$$ (7)

where *h*_*0*_ is the total global harvest in the base year, *p*_*0*_ is the global fish price in the base year, and *ε* is the constant elasticity of demand.

#### Fleet response

Our fleet response module assumes that the fishing costs of fleet *j* in a given year are a function of that fleet’s fishing effort in the current year (*e*_*j*,*t*_) and a fleet-specific cost coefficient (*α*_*j*_, time-invariant) as follows:

$$c_{j,t}=\alpha_{j}{e_{j,t}}^{\beta }$$ (8)

where *β* is a scalar cost parameter that determines the shape of the cost curve (i.e., how non-linear costs are).

We assume that fisheries profits are calculated as revenue less costs plus subsidies for each fleet. Revenue for fleet *j* in the current year is *p*_t_*h_*j*,*t*_. Costs for fleet *j* are given by Eq 8. Profits for fleet *j* in a given year (*π*_*j*,*t*_) are therefore equal to:

$$\pi_{j,t}=p_{t}h_{j,t}-c_{j,t}+s_{j,t}e_{j,t}$$ (9)

where *s*_*j*,*t*_ is the rate of subsidization (i.e., how much each unit of effort is being subsidized, expressed in units of 2018 USD/kWh) for fleet *j* in the current year, and *e*_*j*,*t*_ is fleet *j*’s total fishing effort in the same year.

Fishing fleets in our model engage in rational fishing behavior, such that equal marginal profits exist from effort (the ‘ideal free distribution’). Fleets will therefore continue to increase their effort so long as there are profits to be made [33]. In equilibrium, marginal profits in all areas will be zero, and differences in cost structure will define differences in effort across fleets. Assuming that global fishery profits are equal to 0, we can estimate the cost coefficients for each fleet as follows:

$$\alpha_{j}=\frac{{(p}_{0}*h_{j,0})+{(s}_{j,0}*e_{j,0})}{{e_{j,0}}^{\beta }}$$ (10)

where *p*_*0*_ is the global fish price in the base year, *h*_*j*,*0*_ is the total harvest by fleet *j* in the base year, *s*_*j*,*0*_ is the rate of subsidization of fleet *j* in the base year, and *e*_*j*,*0*_ is the total fishing effort exerted by fleet *j* in the base year. In solving for the cost coefficient for fleet *j* in this way, we are accounting for the role that subsidies play in artificially lowering fishers’ operating costs.

Effort in the next year for fleet *j* (*e*_*j*,*t+1*_) adjusts in response to profits and the management regime of the fleet. For fleets considered to be in open-access (denoted by the subscript *OA*) this is given by:

$$e_{OA,t+1}=\eta \pi_{OA,t}+\;e_{OA,t}$$ (11)

where *η* is a parameter that regulates the speed at which effort enters and exits the fishery. For managed fleets (denoted by the subscript *M*) this is given by:

$$e_{M,t+1}=\omega \pi_{M,t}+\;e_{M,t}$$ (12)

where *ω* is again a parameter that regulates the speed at which effort enters and exits the fishery, but where *ω* <<< *η*.

#### Model parameterization

Similar to works that inspired our model [27, 28], the base form of our analysis considers all global marine fisheries as one large fishery. It is a typical aggregate fisheries model based upon fisheries economics theory and empirical knowledge and is parameterized at the global level. See section 3.2.1 and Tables D and E in S1 Appendix for more information on parameterization for the global analysis. This type of model is a simplified characterization of the global fishery and is not designed to analyze the performance of individual fisheries. Modeling the world’s fisheries as a global stock allows for the easy assessment of different subsidy reform policies in a robust and transparent way due to its simplicity. However, most countries are obviously interested in assessing the effects of different reform policies on a more localized scale. The tool therefore also includes the ability to explore results at a regional level, though we note that the regional results are likely to be less reliable than the global results [27]. See section 3.2.2 and Tables E and G in S1 Appendix for more information on parameterization for the regional analysis.

### Characterization of fishing fleets and their behavior

It is highly unlikely that all fishing vessels will be affected by reform, and it is possible that unaffected vessels may actually increase their fishing effort in response to increased biomass and/or prices. This potential “rebound effect” could reduce the effectiveness of a subsidy reform policy (Fig A in S1 Appendix) and is important to consider. Rebound effects could occur in response to 1) increases in biomass over time resulting from decreased effort (and therefore catches) by the reform-affected fleet, 2) increases in fish price resulting from decreased supply from the reform-affected fleet, or 3) some combination of the two.

In order to capture these effects, we use the additional characteristics assigned to our global vessel list to create four fishing fleets in our model: two reform-affected fleets (managed and open-access, hereafter denoted by the subscripts *M-A* and *OA-A* respectively) and two reform-unaffected fleets (managed and open-access, hereafter denoted by the subscripts *M-U* and *OA-U* respectively). The distinction between managed and open-access is made using one of the two indicators of fishery management effectiveness defined above. For simplicity, we set the threshold separating managed from open-access fleets at the 50% quantile of management scores, but users have the option to change this in the SubsidyExplorer toolkit. The sizes and compositions of each fleet depend on the subsidy reform proposal(s) or disciplines(s) that the user selects in the SubsidyExplorer toolkit (Fig 1, 3a).

To illustrate the rebound effect described above and to explain how fleet characterization works in SubsidyExplorer, consider the following simple example. There are ten fishing vessels, five of which are classified as managed (*M*) vessels based on the area in which they fish, and the remainder are considered to be in open-access (*OA*). All vessels are currently subsidized, and the total amount of subsidies received by managed vessels is the same as that received by open-access vessels. We want to model the effects of a discipline prohibiting subsidies to all vessels partaking in IUU fishing. If one managed vessel, and two open-access vessels fulfill this criteria, the sizes and compositions of the four fleets are defined as follows: Fleet *M-U* includes the 4 vessels fishing in a managed area that have not been identified as partaking in IUU fishing; fleet *M-A* includes the 1 vessel fishing in a managed area that has been identified as partaking in IUU fishing; fleet *OA-U* includes the 3 vessels fishing in an open-access area that have not been identified as partaking in IUU fishing; fleet *OA-A* includes the 1 vessel fishing in an open-access area that has been identified as partaking in IUU fishing.

The total characteristics of each of the four fleets are then defined within the SubsidyExplorer toolkit by aggregating the base year characteristics of their component vessels (catches, subsidies, effort, and revenues) (Fig 1, 3b). In our simple example, this is very straightforward as only fleets *M-U* and *OA-U* contain more than one vessel, but in the tool, all four fleets usually contain multiple vessels. Catchability and cost coefficients are estimated for each fleet based on Eqs 4 and 10.

When fisheries receive capacity-enhancing subsidies, their costs are artificially lowered (captured in the cost coefficients calculated above). In unmanaged or poorly-managed fisheries, fishing effort is often directly coupled with profit, which is why subsidies that lower costs (or increase revenues) can drive overfishing. In well-managed fisheries, effort and profits are often decoupled, with effort (or catches) being determined externally. Capacity-enhancing subsidies can still incentivize increases in effort in well-managed fisheries depending on the degree to which subsidies and profits are decoupled (i.e., the effectiveness of management), but likely to a lesser degree than in unmanaged or poorly-managed fisheries. We apply this logic to our four fleets.

Once our four fleets have been defined and their cost coefficients have been calculated, prohibited subsidies with the potential to be capacity-enhancing are removed from the two directly affected fleets (*OA-A* and *M-A*) and the model projection begins (Fig 1, 3c). The actual type(s) and magnitude(s) of subsidies removed from these fleets depend on the specific disciplines selected as part of the proposal being modeled, but generally following the following criteria: a) For disciplines pertaining to IUU and overfished stocks, all capacity-enhancing subsidies are removed from vessels triggering a prohibition, b) for disciplines pertaining to overcapacity and overfishing, only the identified type(s) (and/or magnitude(s), in the case of cap-based proposals) of capacity-enhancing subsidies are removed from the vessels triggering a prohibition. This means that the magnitudes of subsidies allocated to the two affected fleets post-reform may not be equal to zero. The two unaffected fleets (*OA-U* and *M-U*) retain all of their original subsidies.

Our model of how fleets respond to changes in subsidies is consistent with theoretical and empirical works that have explored the effects of subsidies on fishing behavior. In the absence of effective control of fishing effort (i.e., in open-access fisheries), subsidies that improve the profitability of the fishery will result in increases in fishing effort as participants race to capture a greater share of the profit until it has been dissipated as a result of increased catches (and increased costs) [34]. Effective management may be able to protect against some of the negative overfishing effects of subsidies [22], but overcapacity can still occur, causing losses in welfare and international distortions in trade [35]. Removing profit-enhancing subsidies will result in short-term economic losses as fishers’ costs increase, but will ultimately lead to ecological and economic gains in the long-term [35].

This theory of change manifests in our model in the following way. Once the reforms are implemented in our projection model, all four fleets begin with their respective base year efforts, but the two fleets from which subsidies have been removed face higher costs and will therefore end up with negative profits. As a result, they will adjust their effort in the next time step in response as specified by Eqs 11 and 12:

$$e_{OA-A,t+1}=\eta \pi_{OA-A,t}+\;e_{OA-A,t}$$ (13.1)

$$e_{M-A,t+1}=\omega \pi_{M-A,t}+\;e_{M-A,t}$$ (13.2)

Parameters *ω* and *η* limit the degree to which these fleets can decrease their effort if profits are negative (i.e., *π*_*OA-A*,*t*_ *< 0* or *π*_*M-A*,*t*_ *< 0*) in a single time step (with *η* allowing for a much greater response than *ω*). Accordingly, reforms that affect the open-access fleet will result in greater decreases in effort. Eventually, as the effort in the two affected fleets decreases in response to the subsidy reforms, effort in the two unaffected fleets may increase (i.e., the rebound effect) in response to the changes in biomass or price described above that have increased profits. The effort of these two fleets in the next time step is also dictated by Eqs 11 and 12 as follows:

$$e_{OA-U,t+1}=\eta \pi_{OA-U,t}+\;e_{OA-U,t}$$ (14.1)

$$e_{M-U,t+1}=\omega \pi_{M-U,t}+\;e_{M-U,t}$$ (14.2)

Parameters *ω* and *η* similarly limit the degree to which these fleets can increase their effort if profits are positive (i.e., *π*_*OA-U*,*t*_ *> 0* or *π*_*M-U*,*t*_ *> 0*) in a single time step. As a result of these multi-fleet dynamics, the SubsidyExplorer toolkit can corroborate the theory of change associated with reforming fisheries subsidies by simulating the effects of any given subsidy reform package, and helping WTO negotiators understand the complex rebound effects likely to result.

### Application development

Open-source tools, such as the Shiny framework (hereafter, “Shiny”) for the R programming language, are an ideal platform for building web-based decision support tools. They allow for easy integration of statistical models and analyses from R into a user-friendly interface. The SubsidyExplorer toolkit was developed using the Shiny web framework for R version 3.6.2 [36]. The following packages were instrumental to the creation of this decision support tool: *tidyverse* for data wrangling and plotting [37], *shiny* and *shinydashboard* for the user interface framework [38, 39], *plotly* for interactive figures [40], and *leaflet* for interactive maps [41]. All code and data used in the creation of SubsidyExplorer are available at https://doi.org/10.5281/zenodo.5593733.

## Results and discussion

### SubsidyExplorer as a decision-support tool

**Fig 2:**
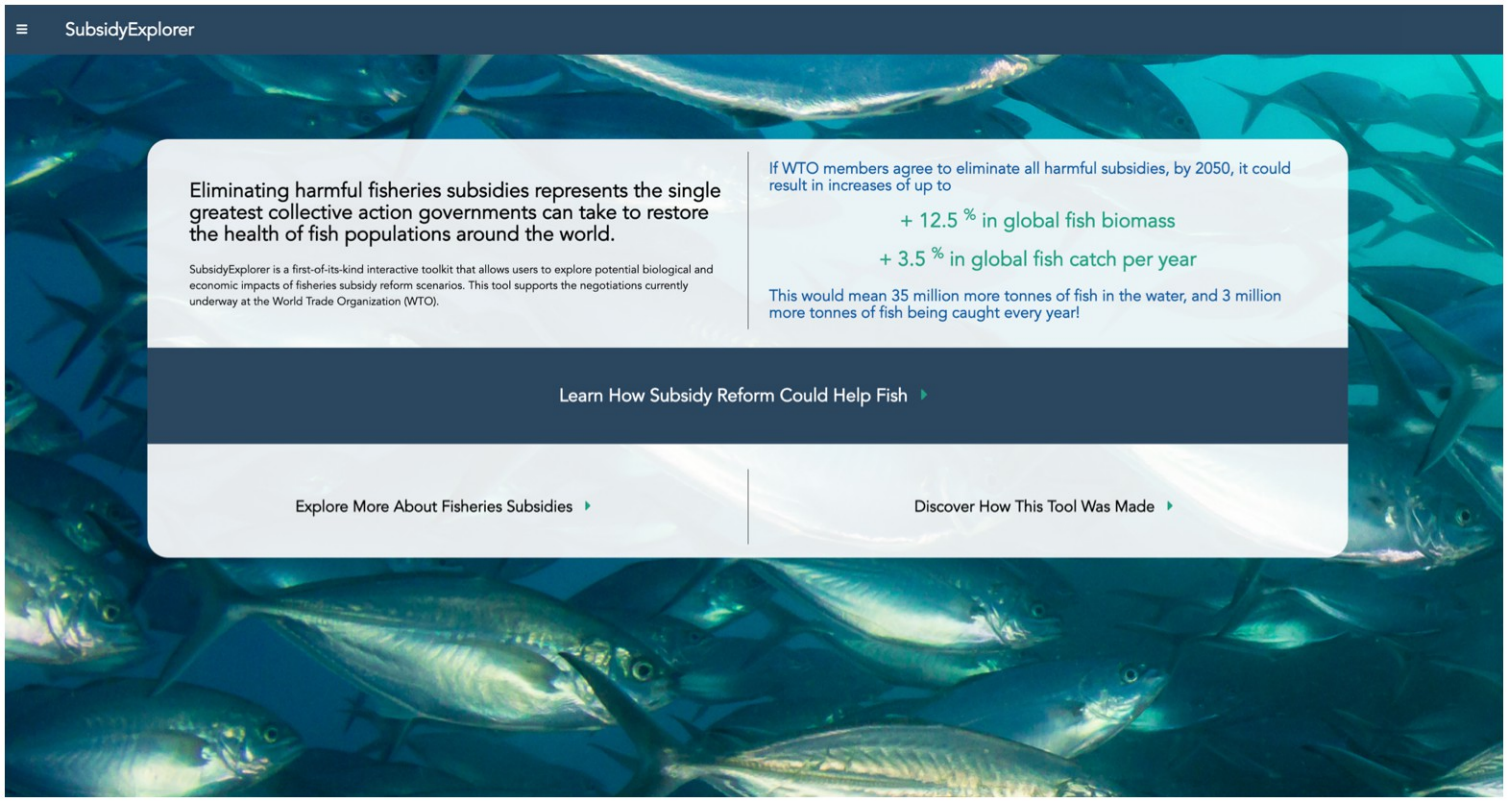
SubsidyExplorer landing page. The first page of SubsidyExplorer provides contextual information on the purpose of the app, as well as a summary of high-level results. This page presents the user with three options of how to proceed. The first option, “Learn How Subsidy Reform Could Help Fish”, takes users to the app’s primary feature: a decision-making framework for comparing different subsidy reform proposals. The second option, “Explore More About Fisheries Subsidies’’, takes users to a section devoted to data exploration. Finally, the third option, “Discover How This Tool Was Made”, houses resources and more information on the methods underlying the tool. Republished from www.subsidyexplorer.com under a CC BY license with permission from UCSB, original copyright 2021.

SubsidyExplorer is a one-of-a-kind tool that 1) enables users to compare different subsidy reform proposals that the WTO is currently negotiating, and 2) provides users with other relevant information and helpful data visualizations on global fisheries subsidies. Here we describe the design and utility of SubsidyExplorer. The app is divided into three key sections, all of which can be accessed from the landing page (Fig 2). The first section contains the aforementioned decision-making framework for comparing subsidy reform policies, and navigation through this section of the app roughly parallels the steps in that framework. The second section gives users an opportunity to explore the data underlying the tool. Finally, the third section provides additional information and resources on the methods used to develop the app.

**Fig 3:**
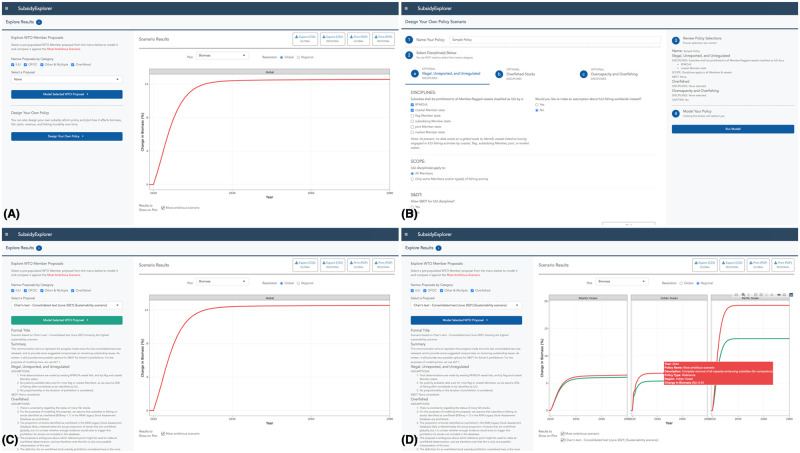
Learn How Subsidy Reform Can Help Fish: Policy comparison portal. The first section of SubsidyExplorer executes the tool’s primary feature: a decision-making framework for comparing different subsidy reform proposals. Within this section, the user can (A) select from proposals that have been submitted to the WTO, (B) design their own policy, (C) run a global bioeconomic projection model, and (D) evaluate the global and regional effects of the selected policy on different biological and economic indicators. Republished from www.subsidyexplorer.com under a CC BY license with permission from UCSB, original copyright 2021.

In the first section of the tool, the user is presented with two options for comparing policies: 1) choose among existing subsidy reform proposals that different delegations have submitted to the WTO or 2) create a new subsidy reform proposal by selecting from individual disciplines. To examine an existing proposal, the user can select a proposal that has been submitted to the WTO using a dropdown menu (Fig 3A). Alternatively, to create a new proposal, the user can use the “Design Your Own Policy” button at the bottom of the left panel to proceed to manual discipline selection (Fig 3B). If users select an existing proposal from the menu, additional options and information appear in the left panel to initiate the second step of the framework (Fig 3A): Learn about the chosen proposal and any existing data limitations, and make assumptions to overcome these limitations if necessary. Once a selection is made from either section, the user can initiate the third step in the decision-making process by clicking on the button to run the bioeconomic model (Fig 3C). Lastly, the figure and surrounding widgets allow the user to evaluate the effects of the chosen proposal on different biological and economic indicators and assess the regional breakdown of impacts (Fig 3D).

**Fig 4:**
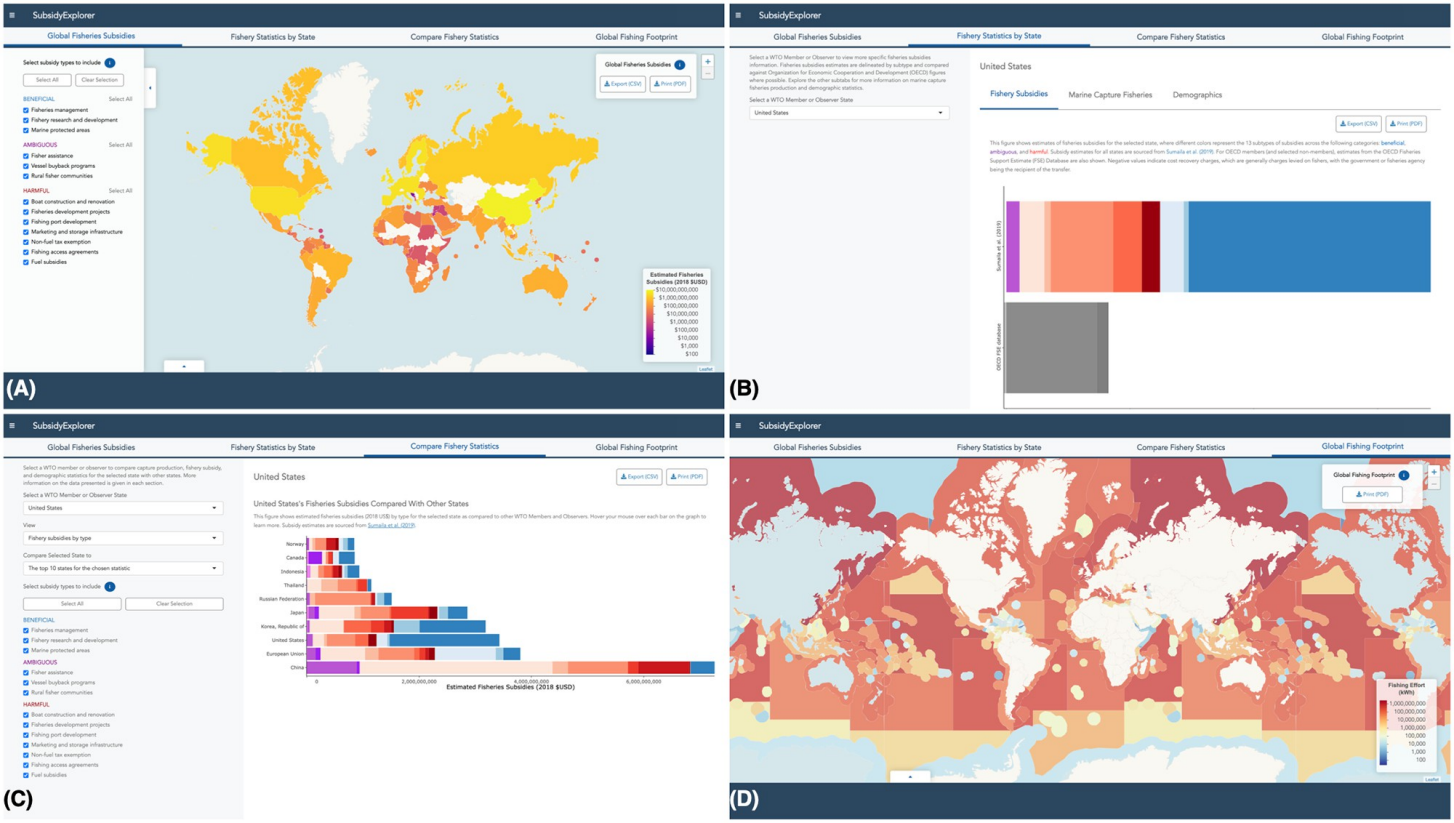
Explore More About Fisheries Subsidies: Data exploration portal. The second section of SubsidyExplorer serves to satisfy the tool’s secondary objective: to allow users to view and interact with data on fisheries subsidies in the context of other relevant fisheries information. Within this section, the user can (A) explore data on subsidy provisioning by country, (B) learn more about the makeup of fisheries subsidies provided by different countries (as well as other types of fisheries and demographic data), (C) compare absolute and relative metrics across countries, and (D) explore patterns of fishing effort globally. Land boundaries depicted in (A) and (D) were made with Natural Earth (free vector and raster map data @ naturalearthdata.com). Marine boundaries depicted in (D) were made using the Maritime Boundaries Geodatabase, version 10 [49]. Republished from www.subsidyexplorer.com under a CC BY license with permission from UCSB, original copyright 2021.

In the second section, users can explore the tool’s underlying data in four sections: global fisheries subsidies (Fig 4A), fishery statistics by state (Fig 4B), compare fishery statistics (Fig 4C), and global fishing footprint (Fig 4D).

### Maximizing the impacts of fisheries subsidies reforms: Results and key takeaways

**Fig 5:**
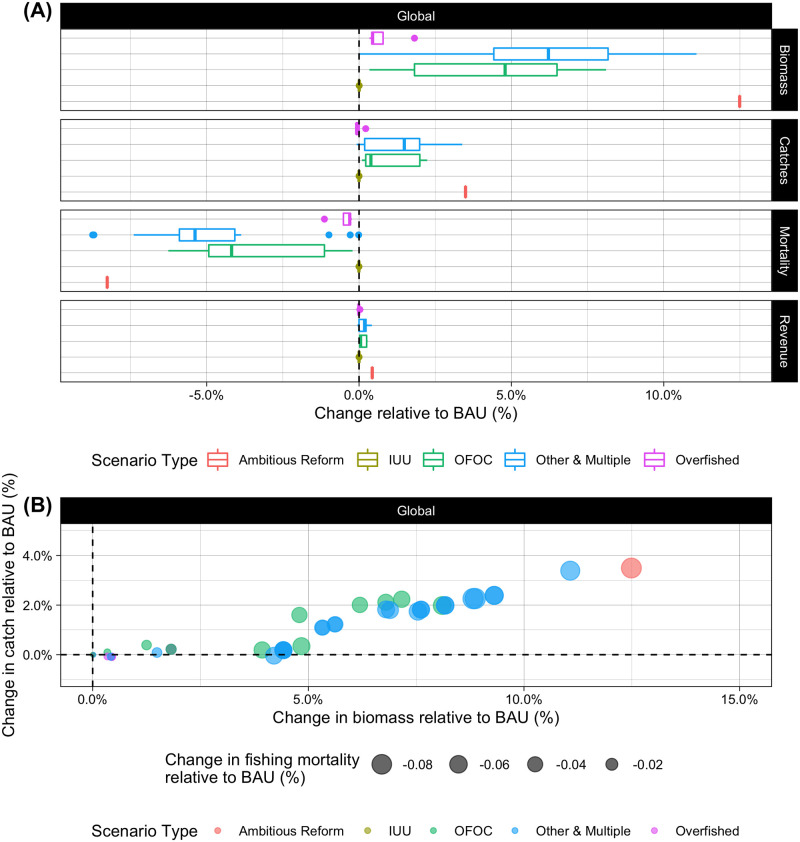
Global simulation model results (2050) for all pre-populated subsidy reform proposals included in SubsidyExplorer. In panel (A), changes in biomass, catch, fishing mortality, and revenue (%) are shown for different proposal types relative to a business as usual (BAU) scenario in which subsidy provisioning continues unchanged from 2018. The type of reform proposal is denoted by the color. Proposals may pertain to reforming subsidies to illegal, unreported, and unregulated (“IUU”) fishing, subsidies to fishing on overfished stocks (“Overfished”), subsidies contributing to overcapacity and overfishing (“OFOC”), or to multiple types (“Other & Multiple”). The “Ambitious Reform” scenario represents complete removal of all subsidies with the potential to be capacity-enhancing and thus represents the upper bound of effects seen from this model. Panel (B) shows changes in biomass (%) relative to BAU on the x-axis and changes in catch (%) relative to BAU on the y-axis for each individual proposal. Changes in fishing mortality (%) relative to BAU are denoted by the size of the point. The type of reform proposal is still denoted by the color of the point.

The SubsidyExplorer tool yields many insights on the likely magnitudes and distributions of effects associated with the three main categories of fisheries subsidies disciplines: 1) subsidies to IUU fishing, 2) subsidies to fishing on overfished stocks, and 3) subsidies contributing to overcapacity and overfishing. Here we present the global results from all of the pre-populated subsidy reform scenarios contained within the tool (Fig 5 and Table J in S1 Appendix; regional results are presented in Fig F and Tables K-M in S1 Appendix) and discuss general takeaways regarding the potential effects of different subsidy reform disciplines within each focus area. We also highlight specific questions and ongoing challenges that WTO negotiators are facing to demonstrate how SubsidyExplorer can be a useful tool for overcoming them.

A key feature of the SubsidyExplorer toolkit within the “Learn How Subsidy Reform Can Help Fish” portal is the ability of a user to design their own subsidy reform proposal. Because of the many different disciplines that could be chosen and the wide range of thresholds that could be explored for those disciplines, there is a wide range of possible results one could obtain from using the SubsidyExplorer tool. Nonetheless, this advanced functionality will only appeal to certain users. Most users will be primarily interested in comparing the results from the proposals being considered by WTO delegates, which are shown in Fig 5. Within SubsidyExplorer, subsidy reform scenarios are assessed based on the changes in total biomass, catch, revenue, and fishing mortality that would be likely to result relative to a “business as usual” (BAU) scenario in which subsidy provisioning would continue indefinitely as it was in the base year. Results are therefore presented as the percent change in those four metrics relative to the BAU scenario. Fig 5A shows the ranges of global effects for different types of proposals. Fig 5B shows the percent changes in global biomass, catch, and fishing mortality relative to BAU for all 44 individual pre-populated proposals represented in SubsidyExplorer as of March 2022 (presented individually in Table J in S1 Appendix). The percent change in fishing mortality relative to BAU is given by the size of the points. Most of the early proposals put forth to the WTO were limited to a single category of disciplines, though many of the more recent proposals incorporate disciplines from all three categories of fisheries subsidies (included as “Other and Multiple” in Fig 5). We therefore use the three categories of disciplines to summarize and discuss global results and key takeaways from different proposals in the following sections.

#### Subsidies to illegal, unreported, and unregulated (IUU) fishing

Though there is general consensus among WTO Members that there should be no subsidies supporting IUU fishing, we find the likely effects of all potential disciplines relating to IUU fishing, as demonstrated in SubsidyExplorer, to be trivial relative to BAU given current data availability on IUU operators. Proposals focused predominantly on disciplining subsidies supporting IUU fishing, on average, only result in an increase in biomass of 0.005% relative to BAU by 2050 (Fig 5). Effects on catch, revenue, and fishing mortality are also projected to be effectively nil.

To understand the reason for this finding, consider the number of vessels likely to be affected by one approach to identifying IUU fishing that is called for in many proposals: to eliminate subsidies to vessels that appear on RFMO IUU vessel lists (e.g., IUU (Option A)). By searching global RFMO IUU lists [42], we find that fewer than 175 vessels of the 2.8 million motorized fishing vessels estimated to operate across global oceans in 2018 had been identified as partaking in IUU activity. From our global database of industrial fishing vessels, we found that these vessels as a group accounted for less than 1% of all industrial fishing effort in 2018. Accordingly, it is unsurprising that projections from SubsidyExplorer suggest trivial effects on biomass, catches, revenue, and fishing mortality.

It is important to note that any calculations relating to this class of disciplines are hampered by significant data limitations, and therefore, these disciplines should not be written off entirely. Many proposals containing IUU disciplines include alternative methods of identifying IUU activity that would trigger the prohibition (other than relying on RFMO lists, as described above). These could include allowing coastal- and flag-states the ability to make such determinations so long as the processes by which they are made are fair and transparent, or allowing the subsidizing-state to make such a determination unilaterally. As most of these entities do not currently maintain their own lists of IUU operators (or at least do not make them publicly available), we cannot model the effects of many potential disciplines in this category. Therefore, including IUU disciplines in a final agreement could very well have a greater impact than our model suggests. Additionally, disciplines related to IUU fishing could have values that are well outside the scope of our model, such as acting as a consensus-building foundation for a global agreement, and sending a strong anti-IUU message [43].

#### Subsidies to fishing on overfished stocks

The second category of disciplines under negotiation relates to the prohibition of subsidies for fishing on overfished stocks. To date, Members have struggled to reach an agreement on an approach for disciplining such subsidies. This lack of consensus is largely due to the fact that it is challenging to define an “overfished stock” and the data needed to make such determinations (as well as the money and resources required to collect such data) do not exist in many geographies. Additionally, this process is hampered by the fact that it is nearly impossible to establish a causal relationship between any one Member’s subsidies and the overfished status of a stock.

Although we are unable to estimate truly causal relationships, we rely on a simpler metric to generate insights related to reforming subsidies based on stock status. We analyze the effects of prohibiting subsidies for fisheries that occur in the geographic range of a stock that is known to be overfished. Stock status is relatively well documented for many of the world’s largest and most lucrative fisheries [25] and the geographic boundaries of these stocks have been well-mapped [44]. Combining these ranges with our database of global industrial fishing activity, we find that 28% of time spent fishing by industrial vessels in 2018 occurred in areas where the median stock status was overfished (defined here as having a B/B_MSY_ of less than 1). However, many of these fisheries are also among the most highly regulated and are already benefiting from reduced exploitation rates. Disciplines targeting such fisheries are unlikely to yield effects as significant as those impacting fisheries that lack stringent control measures [22, 45]. Accordingly, we find that proposals focusing on disciplining subsidies for fishing on overfished stocks can have meaningful effects compared to a BAU scenario, depending on the assumptions made with regards to defining an overfished stock. Proposals focused predominantly on disciplining subsidies to fishing on overfished stocks result in increases in biomass of 0.34–1.82% relative to BAU by 2050, changes in catch ranging from -0.09% to 0.22%, and decreases in fishing mortality of 0.24–1.14% (Fig 5).

As demonstrated in the SubsidyExplorer toolkit, several variables can have major implications for the potential impacts of an overfished subsidy discipline. These variables include the management measures in place in subsidized fisheries, the definition of an overfished stock, and the entity responsible for making the overfished determination (i.e., data availability and/or source). To better understand these challenges, consider two hypothetical subsidy reform scenarios from the SubsidyExplorer toolkit. In the first, overfished stock determinations are made based on the RAM Legacy Stock Assessment Database [25] (many of the fisheries included in this database are highly regulated). In the second, data-limited methods are employed to estimate stock status for fisheries lacking formal stock assessments (many of which are lacking effective regulation [24]. Using the same definition of an overfished stock (B/B_MSY_ < 1) for both scenarios, a discipline relying upon the first method of making an overfished stock determination (e.g., Comprehensive text proposal) would only yield a 1.82% increase in global fish biomass by 2050 compared to BAU according to the SubsidyExplorer model. Alternatively, a discipline using the second method of making an overfished stock determination—an assumption that can be made in the “Design Your Own Policy” section in SubsidyExplorer—could increase global biomass by 5.76% over the same period. Though the first method covers the world’s largest fisheries from which the majority of capture production originates, the disproportionately large effect seen from the second is largely driven by the fact that many smaller fish stocks, which are often caught alongside or in addition to bigger stocks, are in poorer health or the fisheries management scores are lower in regions where these stocks exist.

An alternative approach that some proposals have advocated for is a “negative effects’’ approach in which disciplines would only apply if a (negative) causal relationship could be proven to exist between a subsidy and stock status. However, given the lack of available information on subsidy provision, uncertainty around the status of most individual fish stocks, and the difficulty of establishing a causal relationship between the two, the impact of such an approach would likely be limited, as more data would be needed to trigger the prohibition. Data do not readily exist on a global scale to establish such causality, but one potential proxy could be to consider a more conservative definition of an overfished stock. Using the SubsidyExplorer tool, the two methods for determining an overfished stock, as presented in the previous paragraph, result in even smaller increases in global fish biomass in 2050 relative to BAU (0.44% and 2.37%, respectively) if a more conservative definition of an overfished stock is used (B/B_MSY_ < 0.8). We used such an approach to represent the more conservative language of some proposals disciplining subsidies to fishing on overfished stocks in SubsidyExplorer (e.g., Overfished—Negative effects + rebuttable (Option B)).

### Subsidies that contribute to overcapacity and overfishing

The final, and arguably the most impactful, category of subsidy disciplines under negotiation relates to the prohibition of subsidies that contribute to overcapacity and overfishing. These subsidies are thought to be at the root of the problem and, therefore, removing all subsidies in this category should theoretically have the single largest effect on global fisheries health. Indeed, from our database of industrial fishing activity we find that nearly 99% of all industrial fishing effort originates from states that provide these types of fishery subsidies [2, 20], and thus it is unsurprising that we find that proposals focusing on disciplining subsidies that contribute to overcapacity and overfishing have the greatest effects compared to BAU. Proposals focused predominantly on disciplining subsidies contributing to overcapacity and overfishing result in increases in biomass of 0.34–8.11% relative to BAU by 2050, increases in catch ranging from 0.10–2.24%, and decreases in fishing mortality of 0.21–6.26% (Fig 5).

Though it is likely unrealistic that WTO negotiations will result in the complete removal of subsidies that contribute to overfishing and overcapacity, it is a helpful exercise to illustrate what this “most ambitious” subsidy reform scenario would look like. Built into SubsidyExplorer is a reference scenario in which all subsidies with the potential to be capacity enhancing are removed (the red point in Fig 5). In such a scenario, global fish biomass could increase by 12.49% and global catch could increase by 3.49% annually relative to BAU by 2050. This would be roughly equivalent to having 35 million more tonnes of fish in the ocean by 2050, while also catching 3 million more tonnes of fish each year.

Though the potential benefits of this discipline are significant, Members struggle to reach consensus about which types of subsidies to prohibit in this category. Given the heterogeneity in global subsidy programs, a list-based approach—in which Members negotiate a fixed set of qualifying subsidy types or programs that contribute to overcapacity and overfishing—requires a great deal of finesse to both be succinct enough to reach agreement on and detailed enough to represent the heterogeneity in Members’ subsidy programs.

The alternative cap and tier approach where each Member would have a maximum subsidy amount that could be dispersed has received varying degrees of support. As demonstrated in SubsidyExplorer, the potential effects of such an approach on global biomass are variable (but not necessarily insignificant) depending on how the tiers are defined, and whether the subsidy cap is determined based on a percentage of landed value, a percentage of existing subsidies, or some other formula. Additionally, a cap-based approach does better reflect the individual circumstances of different Members without introducing blanket exemptions by allowing for different rules to be applied to different countries based upon the tiering criteria. For example, the four cap-based proposals submitted to the WTO that are included in SubsidyExplorer could yield a range of possible effects on global biomass relative to BAU, from increases of 0.34–7.17%. A big concern with a capping approach is that it could result in maintenance of the status quo (or an agreement with little impact) if rules related to the cap are too slack.

For example, some of the cap-based proposals include a set of “green box” subsidies that would be exempt from the cap. This feature has the potential to limit the impact that this kind of proposal could have. Negotiations around which subsidy types would qualify as “green box” brought back many of the same stalemates from earlier attempts to identify prohibited subsidies using a list-based approach, resulting in many Members preferring to revert back entirely to a list-based approach. Furthermore, the inclusion of “green box” subsidies presents a potential way in which disciplines that might otherwise result in meaningful subsidy reductions could be circumvented. This is a common criticism of carbon cap and trade programs in which certain sectors (such as transportation or agriculture) are often exempt [46, 47], and is another reason why support for a cap-based approach has dwindled.

While the debate between a list- or cap-based approach has been central to this category of disciplines, two secondary issues have arisen: prohibiting subsidies that support fishing activities in areas beyond a Members’ jurisdiction (on the high seas or in the EEZs of other states) and the S&DT that should be allowed for developing and LDC Members. Only 6% of global marine capture landings come from fishing on the high seas [15], but prohibiting subsidies to fishing in these areas has been a potential source of agreement among Members. Nonetheless, very few vessels fish solely on the high seas. Therefore, the potential impacts of such a discipline would be dependent on the thresholds used to identify subsidy programs or recipients likely to trigger a prohibition. For example, if we define a high-seas vessel as one that spends at least 95% of its time fishing on the high seas, we identify 2,144 vessels from our global vessel database fitting this description in 2018. Together these vessels are responsible for approximately 16% of global industrial fishing effort. However, defining a high-seas vessel as one that spends at least 5% of its annual fishing activity on the high seas, we identify 4,075 vessels, accounting for approximately 34% of global industrial fishing effort. Exploring such alternative thresholds in the ‘Design Your Own Proposal’ section of SubsidyExplorer, we find that a potential prohibition of all subsidies with the potential to be capacity enhancing based on the first definition of high seas fishing might only result in a 1.20% increase in global biomass relative to BAU, while the second could yield an increase of 3.90%. Nonetheless, measures targeting high seas fishing could have meaningful equality implications [48], which could help to explain why reforming subsidies for high seas fishing has received a great deal of attention and support from many Members.

While the negotiation is still fraught with challenges, model runs from the SubsidyExplorer tool suggest that to reach a truly ambitious final agreement, substantial reforms for capacity-enhancing subsidies must be included. Given the ubiquity of capacity-enhancing fisheries subsidies, reforming them will undoubtedly result in significant near-term costs for fishers. Therefore, WTO Members should also focus on developing mechanisms that would make this an equitable transition, such as repurposing existing subsidies and directing them to benefit fishers in a way that does not incentivize additional fishing [45].

## Conclusions

While there may be no silver bullet to reforming fisheries subsidies, there exists a dramatic difference in the level of ambition of different types of subsidy disciplines under consideration by WTO negotiators, and therefore their likely biological and economic effects. Here we have demonstrated how a decision-support tool—SubsidyExplorer—can help to overcome some of the information gaps that have persistently hampered WTO subsidy negotiations by allowing negotiators to explore the potential outcomes of their proposals within a straightforward and comparable framework. Many of the insights generated by this tool are powerful and could have meaningful implications for the ongoing negotiations in 2022. Nonetheless, the modeling framework contained within SubsidyExplorer, as well as those employed in similar global aggregate analyses, is intentionally simplified and strategic, rather than tactical. This is a key limitation of such an approach, and tactical implementation of fisheries subsidies reforms should draw on far more granular data and detailed modeling approaches than were used in this analysis.

Uncertainties and disagreements regarding management of the world’s oceans will undoubtedly continue into the future, encompassing issues beyond just reforming fisheries subsidies. A better understanding of the likely effects of potential interventions can be beneficial to policy makers in such situations, and open access platforms that allow for alternatives to be analyzed and assessed in a comparable and easily understandable way can help to make this possible. Here we have shown how scientific analyses and decision support tools such as SubsidyExplorer can be developed and leveraged to help policy makers achieve more effective evidence-based management. The development of such decision-support tools by the scientific community is a tangible way to inform ocean management into the future.

## Supporting information

S1 Appendix Supplementary information for the SubsidyExplorer toolkit. This appendix contains detailed information pertaining to raw data sources, the data processing workflow, the bioeconomic model, and subsidy reform proposals submitted by Members to the WTO. This file also includes supplementary figures and tables.
(PDF)
