# Supplementary material for: SubsidyExplorer: A decision-support tool to improve our understanding of the ecological and economic effects of reforming fisheries subsidies
Source: PLoS One. 2022 Jun 3;17(6):e0265829. doi: 10.1371/journal.pone.0265829 (PMC9165768; doi:10.1371/journal.pone.0265829)
Supplement: S1 Appendix — This appendix contains detailed information pertaining to raw data sources, the data processing workflow, the bioeconomic model, and subsidy reform proposals submitted by Members to the WTO. This file also includes supplementary figures and tables. (PDF) [file pone.0265829.s001.pdf]

S1 Appendix

Supplementary information for

SubsidyExplorer: A decision-support tool to improve our understanding of the effects of reforming fisheries subsidies

Katherine D. Millage[1,2*], Vienna R. Saccomanno[1,#a], Matthew M. Warham[1,#b], Laura Lea Rubino[1,#c], Anna Schuhbauer[3], U. Rashid Sumaila[3,4], Christopher Costello[1,5]

Corresponding Author: Katherine D. Millage

Email: kmillage@ucsb.edu

This PDF file includes:

Supplementary materials and methods

1. Raw data sources
2. Data processing
3. Bioeconomic model
4. Modeling subsidy reform proposals

Supplementary figures and tables

SI references

Supplementary materials and methods

All data and code for the SubsidyExplorer toolkit is available at https://doi.org/10.5281/zenodo.5593733 or at https://github.com/emlab-ucsb/SubsidyExplorer. All analysis was done using R version 3.6.2 [1], and the SubsidyExplorer toolkit was built using the *shiny* [2] package. The toolkit can be accessed online at http://www.subsidyexplorer.org/ or at https://emlab-ucsb.shinyapps.io/SubsidyExplorer/.

1. Raw data sources

Data for this analysis came from numerous publicly available datasets, as well as some datasets obtained directly from their creator(s) and used with permission. For ease of description, we sort these datasets into three categories based on the subjects to which they primarily pertain and/or are used for in the SubsidyExplorer tool: 1) demographics, 2) fisheries (subsidies), and 3) fisheries (other).

Demographic datasets (described in section 1.1):

- The World Bank World Development Indicators (WDI) database [3];
- The 2017 Food and Agriculture Organization (FAO) of the United Nations (UN) Yearbook of Fishery and Aquaculture Statistics [4];
- Estimates of the number of full-time equivalent (FTE) jobs in marine capture fisheries from Teh and Sumaila [5].

Fisheries subsidy datasets (described in section 1.2):

- Estimates of global fisheries subsidies from Sumaila et al. [6];
- The Organisation for Economic Co-operation and Development (OECD) fisheries support estimate (FSE) database [7];
- Estimates of global small-scale fisheries subsidies from Schuhbauer et al. [8, 38].

Other fisheries datasets (described in section 1.3):

- The FAO Global Capture Production Database [9];
- Reconstructed global ex-vessel prices of fished species from Melnychuk et al. [10];
- The RAM Legacy Stock Assessment Database (RAMLDB) [11, 12];
- Estimates of global stock status from Costello et al. [13];
- Fisheries management indicators from Melnychuk et al. [14];
- The Combined Illegal, Unreported, and Unregulated (IUU) Vessel List [15].

All code used to process the data listed above, as well as the raw data itself (whenever possible), are included in the "data" folder of the project repository.

This analysis also leverages satellite-derived estimates of global fishing effort from Global Fishing Watch (GFW) [16, 17]. An aggregated version of the GFW data is freely available to download at https://globalfishingwatch.org/, but this analysis draws on higher-resolution data that was available to the authors as part of a research collaboration between emLab and GFW. All code used to process the raw data is included in the project repository, but the raw data is hosted in Google's BigQuery cloud data system and is not publicly accessible.

1.1. Demographic datasets

1.1.1. World Bank WDI database

The World Bank WDI database [3] is a global database containing time series of 1,600 different indicators related to many different facets of development. These data are aggregated to the country, regional, and global level. The WDI data used in this analysis were last downloaded through the WDI package for R [18] on August 6, 2020. Three indicators were extracted for all countries for all available years between 2000-2018: total GDP in current $USD (NY.GDP.MKTP.CD), the contribution to GDP from fisheries, forestry, and agriculture in current $USD (NV.AGR.TOTL.CD), and total population (SP.POP.TOTL).

1.1.2. FAO Yearbook of Fishery and Aquaculture Statistics 2017

The FAO Yearbook of Fishery and Aquaculture Statistics is an annual publication that compiles statistical data on capture fisheries, aquaculture production, employment, commodities production and trade, apparent fish consumption and fishing fleets. Released in 2019, the 2017 issue [4] was the most recent release at the time that SubsidyExplorer was created. The data on the total number of fishers by country from the Employment section of the Yearbook was used in this analysis. This data is either reported directly by each country's fishery agency to the FAO, or is estimated directly by the FAO. For most major fishing countries, data is available for 1995, 2000, 2005, 2010, and 2012-2017. We manually transcribed these data from PDF to CSV format.

1.1.3. Contribution of marine fisheries to worldwide employment (Teh and Sumaila 2011)

Teh and Sumaila [5] compiled available information on marine fisheries employment for 144 countries from the FAO, as well as a number of other peer-reviewed and grey literature sources. Where information was missing, out of date, or considered to be unreliable, they used a proportional transfer approach to estimate the number of persons in each country employed in the marine fisheries sector. They then converted the number of marine fisheries jobs in each country into FTE units to better allow for comparison across countries. Estimates of FTE marine fisheries employment in this database are from 2003. These data were provided to us directly by their creators.

1.2. Fisheries subsidies datasets

1.2.1. Updated estimates and analysis of global fisheries subsidies (Sumaila et al. 2019)

Sumaila et al. [6] identified and compiled published information on financial transfers provided to the fishing sector by governments and estimated the likely magnitudes of fisheries subsidies (in 2018 USD) in countries for which this information was not available. They assign subsidies to one of 13 categories (Table A) based on many factors including the policy objective of the subsidy, the description of the subsidy program, scope, coverage, and duration, sources of funding, the administering authority, annual magnitude, recipients, and mechanisms of transfer [19]. Each category is associated with one of the three subsidy types first described by Sumaila et al. [20]: beneficial ("good") subsidies, capacity-enhancing ("bad") subsidies, and ambiguous ("neutral") subsidies. This work builds upon the estimates previously presented in 2009 USD by Sumaila et al. [19]. These data were provided to us directly by their creators.

By definition, beneficial subsidies are those that lead to investment in natural capital assets (maximize economic rents). In the context of fisheries, these types of subsidies often aim to increase the growth of fish stocks via conservation, allow for improved monitoring of catch rates, or enhance fisheries management to achieve biologically or economically optimal use of the resource. Subsidies included in the following three categories are considered to be "good" subsidies: 1) fisheries management programs and services, 2) fishery research and development (R&D), and 3) Marine Protected Areas (MPAs). Capacity-enhancing subsidies are those that lead to disinvestments in natural capital assets. In the fisheries context, "bad" subsidies allow for fishing capacity to develop beyond the point that would be sustainable in the long term by artificially increasing profits. This overcapacity can then compound overexploitation problems such as overfishing. Subsidies included in the following seven categories are considered to be "bad" subsidies: 1) fuel subsidies and tax exemptions, 2) boat construction, renewal, and modernization, 3) fishing port construction and renovation, 4) price and marketing support (processing and storage infrastructure), 5) fishery development projects and support services, 6) foreign access agreements, and 7) non-fuel tax exemptions.

Ambiguous subsidies are those that may lead to either investment or disinvestment in the fishery resource, often depending on the specific mechanisms of the subsidy program. Subsidies included in the following three categories are considered to be "neutral" subsidies: 1) fisher assistance programs, 2) vessel buyback programs, 3) rural fishers' community development programs.

1.2.2. OECD FSE database

The OECD FSE database [7] measures and describes fisheries support policies across all OECD member countries and selected non-members. The FSE database analyzes fisheries policies and expresses the amount of support they provide to the fishing industry using support indicators, which are comparable across time and between countries. In order to be included in this database, a policy must generate a transfer to fishers, regardless of the nature, objectives or impacts of the policy. 'Transfers' can include budgetary payments such as direct payments to fishers and general support for the fishing sector in terms of management, harbors, and other infrastructure, as well as non-budgetary payments such as tax measures. In order to qualify as a 'transfer', there also must be a clear source (i.e. the group bearing the cost) and recipient (i.e. the group receiving the benefit) of the value. The FSE Manual [21] specifies a very clear set of criteria for identifying the scope of fisheries policies that can be included in the FSE database.

The classification system used by the OECD to identify different types of policies first makes the distinction between non-budgetary transfers to individuals, budgetary transfers to individuals, transfers to the sector generally, and cost recovery charges. Within each of these four categories, policy measures are then classified according to implementation criteria, defined as the conditions under which transfers are provided to fishers (or the conditions of eligibility for the payment). The categories and sub-categories used in the FSE database, as well as brief descriptions of the types of programs included in each are listed in Table B.

These data can be accessed and downloaded directly from the OECD iLibrary portal in several formats (https://stats.oecd.org/Index.aspx?DataSetCode=FISH_FSE). The data used in this analysis were last downloaded as a CSV file on March 10, 2019.

1.2.3. The global fisheries subsidies divide between small- and large-scale fisheries (Schuhbauer et al. 2020)

Schuhbauer et al. [38] identified and compiled published information on financial transfers provided to small-scale fisheries by governments and estimated the likely magnitudes of small-scale fisheries subsidies (in 2018 USD) in countries for which this information was not available. They use the same subsidy types described by Sumaila et al. [20].

The data used in this analysis are published updates of those from [8], previously in 2012 USD. These updated estimates were provided to us directly by their creators and are based upon the global subsidy estimates published in [6].

1.3. Other fisheries datasets

1.3.1. FAO global capture production database

Released on May 21, 2020, version 2020.1.0 of the FAO global capture production database [9] contains an annual time series of capture production statistics from 1950 - 2018. This dataset can be accessed and downloaded directly in several formats from http://www.fao.org/fishery/statistics/global-capture-production/en. The data used in this analysis were last downloaded as a CSV file on July 25, 2019.

The FAO global capture production database includes catches of fish, crustaceans and mollusks, and other aquatic animals, plants, and mammals taken for commercial, industrial, recreational, and subsistence purposes. This dataset does not include production from aquaculture, or discarded catches. All aquatic organisms included in this database are classified according to ~2,400 commercial species items. These species items may comprise a single species, genus, family, or higher taxonomic level. These species items are then matched to the Aquatic Sciences and Fisheries Information System (ASFIS) list of species (~13,000 items). Each species item is also classified into one of 50 different FAO International Standard Statistical Classifications of Aquatic Animals and Plants (ISSCAAP) groups on the basis of taxonomic, ecological, and economic characteristics. These 50 groups constitute nine higher ISSCAAP divisions.

All capture production entries are also classified by fishing area. There are eight major inland fishing areas and 19 major marine fishing areas established for fishery statistical purposes (Fig. B, hereafter referred to as "FAO regions"). Production statistics included in the FAO capture production database are reported to the FAO by national offices.

1.3.2. Reconstruction of global ex-vessel prices of fished species (Melnychuk et al. 2017)

Melnychuk et al. [10] created a streamlined approach to estimate nominal ex-vessel fish prices for all species items reported in the FAO global capture production database. They used three datasets from FAO to reconstruct ex-vessel prices between 1976-2012: 1) global commodities production and trade (1976-2013), 2) world fishery production - estimated value by groups of species (1994-2012), and 3) global capture production (1950-2014). These data were provided directly to us by their creators.

Their method uses three linkage tables to equate export values of fishery commodities, generally reported as the intersection of species (or groups of species) with product types (e.g. fresh; frozen; dried), with

landing records of species. First, they pooled commodities across product types, removing commodities associated with ISSCAAP groups for which a mean ex-vessel price estimate was not available from the FAO. Second, they further aggregated the pooled commodities into ISSCAAP groups in order to compare estimated mean ex-vessel prices by group with those reported by the FAO. Lastly, they linked ASFIS species reported in the FAO global production database to one of the pooled commodities, providing an estimate of average ex-vessel price by year for 1861 of the 2033 ASFIS species associated with landings records. The 172 species for which ex-vessel price estimates were not available came from 14 out of the 50 possible ISSCAAP groups (4 groups of aquatic mammals; 4 groups of aquatic plants; frogs and other amphibians; crocodiles and alligators; turtles; corals; pearls and other shells; sponges).

1.3.3. RAM Legacy Stock Assessment Database (RAMLDB)

The RAMLDB compiles and makes available the results of fisheries stock assessments undertaken by national agencies and regional fisheries management organizations (RFMOs) around the world [11]. These assessments predominantly come from Australia, New Zealand, Canada, the United States, Peru, South Africa, Russia, Argentina, Japan, the European Union (EU), and RFMOs covering multinational tuna and billfish stocks. The proportion of commercially exploited stocks included in the RAMLDB varies significantly by region. The version of the database used in this assessment is 4.491, released on February 20, 2020 [12]. The RAMLDB can be downloaded directly from https://www.ramlegacy.org/. The data used in this analysis were last downloaded on July 25, 2020.

1.3.4. Global fishery prospects under contrasting management regimes (Costello et al. 2016)

The majority of global fish stocks are not assessed with formal stock assessments, and are instead managed using a variety of data-limited methods or are unassessed [22]. No global database exists with the results of these data-limited methods of estimating the status of fisheries for which formal stocks assessments have not been conducted. Costello et al. [13] used data from the FAO Global Capture Production Database to provide estimates of the status of 4,316 fisheries (defined by species-country-FAO region triples) not included in the RAMLDB. These data were provided to us directly by their creators.

1.3.5. Fisheries management impacts on target species status (Melnychuk et al. 2017)

Melnychuk et al. [14] used expert surveys to characterize the fisheries management systems of 28 countries. They evaluated five different attributes of each fisheries management system for each of 10 species in each country: research, management, enforcement, socioeconomics, and stock status. The scores from different attributes were averaged and aggregated by country to calculate a total Fisheries Management Index (FMI) score. We manually transcribed these data to CSV format from Table S2 in the supplementary materials of [14].

1.3.6. Combined Illegal, Unreported, and Unregulated (IUU) Vessel List

Most RFMOs maintain their own lists of vessels that have been found to carry out or support IUU fishing. The Combined IUU Fishing Vessel List was created by Trygg Mat Tracking, a Norwegian non-profit, to consolidate all of the different RFMO IUU lists into one [15]. This dataset can be downloaded directly as an Excel file (https://iuu-vessels.org/Home/Download). The data used in this analysis were last downloaded on November 30, 2019.

1.4. Global Fishing Watch (GFW)

Very few estimates of total fishing effort exist on a global scale, but GFW is a novel way of tracking fishing behavior in near real time on an individual vessel level [16, 17]. GFW has processed more than 22 billion automatic identification system (AIS) positions broadcast by fishing vessels across the world. Designed to help vessels avoid collisions, AIS broadcasts a vessel's identity, position, speed, and turning angle to nearby vessels, and these transmissions are also picked up by satellite- or land-based receivers allowing companies to store and distribute this information. GFW has identified more than 80,000 unique fishing vessels ranging in length from 6 - 146 m. GFW used data on 45,441 marine vessels listed on official fleet registries to train a convolutional neural network (CNN) to identify vessel characteristics. This model can use the behavior of vessels (as broadcast by AIS) to identify six classes of fishing vessels and six classes of non-fishing vessels with 95% accuracy and can predict vessel length, engine power, and gross tonnage.

As it only includes fishing vessels with AIS systems onboard, GFW does not represent the total global fishing effort. The International Maritime Organization (IMO) requires all vessels greater than 300 tons traveling in international waters to have AIS, though certain countries also require smaller vessels to use the device [17]. There is great uncertainty regarding the total number of active fishing vessels in the world, but Kroodsma et al. [16] estimated that the number of vessels with AIS comprised approximately 56% of all vessels larger than 24 m, 9% of vessels 12-24 m, and only 0.2% of vessels under 12 m. They also estimated that vessels with AIS likely contributed between 26% - 34% of the global fishing effort (kW hours expended) of all vessels in the world, with that value increasing to 50% - 70% for all vessels fishing more than 100 nautical miles from shore (halfway to the Exclusive Economic Zone (EEZ) boundary).

The number of vessels with AIS has been increasing greatly since the period covered by the aforementioned study (2012 - 2016) [17], so it is likely that the 2018 data used in this analysis represents more than 34% of global fishing effort of all vessels in the world, and more than 70% of fishing effort for vessels fishing more than 100 nautical miles from shore (both high-end estimates for the period between 2012 - 2016). For more information on the coverage of the GFW dataset, refer to the supplementary information in [16].

2. Data Processing

The objectives of the SubsidyExplorer toolkit are twofold: 1) allow users to explore the potential bio-economic effects of different fishery subsidies reforms, and 2) synthesize and present existing data on fisheries and fishery subsidies that is relevant to the WTO negotiations. Most of the information presented in the latter section is raw data that have been minimally processed, and these data account for many of the inputs into the analysis presented in the former section. Here we briefly describe the processing of the raw data sources discussed above and note which of these processed data feed into the bio-economic model discussed in section 4, and which are solely included for data visualization and exploration purposes.

2.1. Country naming, political entities, and dependencies

Our use of the word "countries" refers to countries, territories, and areas without distinction. The naming of countries varies widely across the data sources used in our analysis, and we recognize that this is often intentional and political. Nonetheless, for this analysis we use the country names recognized by the WTO for display purposes whenever possible. For WTO Non-Members, we use the names recognized by the FAO for display purposes. Our use of a particular name is not meant to convey any opinions regarding the sovereignty or status of any country, territory, or area.

It is important to note that the EU as a political entity is a Member of the WTO, in addition to many of the individual countries that make up the EU. EU countries are often represented at the WTO by the EU delegation—rather than by their respective delegations—which is the case for the fishery subsidies negotiations [23]. For the purposes of this analysis, we consider the EU to be comprised of the following 28 countries (all data used in this analysis dates from 2018 or before): Austria, Belgium, Bulgaria, Croatia, Cyprus, Czech Republic, Denmark, Estonia, Finland, France, Germany, Greece, Hungary, Ireland, Italy, Latvia, Lithuania, Luxembourg, Malta, Netherlands, Poland, Portugal, Romania, Slovak Republic, Slovenia, Spain, Sweden, and the United Kingdom.

There are a number of places in the SubsidyExplorer toolkit where information is presented for the EU as a whole, rather than for the individual countries that make up the EU. Conversely, there are some places where it makes more sense to display information pertaining to EU countries independently. Whenever possible, we aim to show both. In the subsequent sections, where relevant, we will make note of how information pertaining to EU countries was aggregated and/or disaggregated.

There are also parts of our analysis where it is important to associate the activities and/or information related to overseas dependencies with their sovereign states. We try to rely on the positions taken by the WTO regarding the sovereignty or status of different countries when doing this, and these groupings are not meant to represent any options on behalf of the authors on this subject. We will make note where relevant in the subsequent sections of whether information pertaining to dependencies was considered separately from or aggregated into totals for their sovereign states.

2.2. Data processing workflow

The general processing of all datasets discussed in sections 1.1-1.3 is performed and detailed in .../scripts/01_data_wrangling.Rmd in the project repository. All datasets were imported into R where country names were standardized using ISO-3 character codes, and data was converted to a "tidy" format (one row for each data entry) using the *tidyverse* [24], *janitor* [25], *countrycode* [26], and *readxl* [27] packages.

Aggregated entries were created for the EU for all demographic and fisheries subsidies datasets, as well as for the FAO global capture production dataset by summing the values for all EU countries. Data for the overseas dependencies of EU countries were included in these totals whenever available. An aggregate FMI score was calculated for the EU by averaging the FMI scores of all EU countries represented in the data.

We also created aggregate entries for the United States (including data from its overseas dependencies) for the World Bank WDI and the FAO global capture production data.

2.2.1. Extrapolating ex-vessel prices and estimating the landed value of FAO capture production

The database of ex-vessel prices reconstructed by Melnychuk et al. [10] only contains estimates through 2012, and so it was necessary to extrapolate this database to 2018 in order to estimate the landed value of capture production from the FAO in the same year. We used the Consumer Price Index (CPI) from the U.S. Bureau of Labor Statistics [28] to extrapolate ex-vessel prices in 2012 USD into equivalents for each year between 2013-2018. The CPI rates we used are given in Table C. This method of extrapolating ex-vessel prices assumes that the relative values of different fish products remain constant. As ex-vessel prices are dependent on supply and demand, this assumption may not be completely true. However, in the absence of better (and equally comprehensive) ex-vessel data we believe this to provide a reasonable approximation on a global scale.

To estimate the landed value of all species reported in the FAO capture production database, we then match our extrapolated ex-vessel prices in 2018 USD to global capture production from 2018. We first matched ex-vessel prices and landings by species (using both the scientific name and ASFIS species name). For species reflected in the FAO capture production data without matching ex-vessel price estimates, we instead assigned the average ex-vessel price for the ISSCAAP group to which the species belongs. Species from the 14 ISSCAAP groups for which ex-vessel price estimates were not available (4 groups of aquatic mammals; 4 groups of aquatic plants; frogs and other amphibians; crocodiles and alligators; turtles; corals; pearls and other shells; sponges) are assumed to have a price of $0 USD and therefore do not contribute to the total estimates of landed value for each country. Landings of these species do, however, contribute to the total reported capture production for each country.

2.2.2. Calculating relative metrics of fishery subsidization

Using relative rates of fishery subsidization (as opposed to absolute magnitudes) to compare countries has been championed by many WTO delegations, and has been used in previous peer-reviewed studies ([29] is one such example). We therefore calculate a number of relative metrics of fishery subsidization using the subsidy estimates from Sumaila et al. [6] for data visualization purposes. For each of the fishery subsidy types defined by Sumaila et al., we calculate the following:

- Ratio of subsidies to total GDP
- Subsidies per capita (USD/person)
- Subsidies per fisher (USD/fisher)
- Subsidies per FTE marine capture employment (USD/FTE jobs)
- Subsidies per tonne of capture production (USD/mt)
- Ratio of subsidies to total landed value of capture production

Relative metrics are only available if data was available from both sources for a given country. All subsidy estimates used in the calculation of these metrics are in 2018 USD. Additionally, all capture production data are from 2018, and estimates of landed value are in 2018 USD. The most recent year for which data was available for a given country was used for all other metrics.

2.3. Construction of global vessel list

Creation of the global vessel list from GFW is performed and detailed in .../scripts/02_vessel_list.Rmd in the project repository. The *bigrquery* [30] package was used to access the raw GFW data hosted on BigQuery.

The effort, catch, and subsidy data incorporated into the vessel list described in this section is used in the parameterization of the bioeconomic model described in the main text.

2.3.1. Identifying "good" fishing vessels

The raw data used to create the GFW dataset may include transmissions from anything in the ocean with an AIS transponder. Therefore, we take a number of steps to identify and extract data only originating from sources that are truly fishing vessels. We first remove all transmissions from objects that are likely fishing gear (buoys, nets, etc.), rather than vessels. Then, we apply a number of filters to remove transmissions associated with non-fishing and inactive vessels, as well as vessels that may be spoofing their positions. We use the following criteria (suggested to us directly by GFW staff in a training session for research partners) to identify our list of Maritime Mobile Service Identity (MMSI) numbers corresponding to "good" fishing vessels:

- MMSI number must be on the best fishing vessel list created and maintained by GFW;
- MMSI number cannot be used by 2+ vessels with different names simultaneously;
- MMSI number cannot be used by multiple vessels simultaneously for more than 3 days;
- MMSI number has not been found to be offsetting its position;
- vessel class can be inferred by the neural net (i.e. it is an active vessel);
- MMSI number was active for at least 1 day and fished for at least 1 hour in a year;
- MMSI number is associated with fewer than five ship names.

Finally, we cross reference our list of good MMSI numbers with a list of manually identified vessel IDs that are not fishing vessels created by GFW and exclude any vessel identifiers on that list.

2.3.2. Extracting fishing effort and vessel characteristics

Once we create our list of "good" fishing vessels, we then extract the number of hours spent fishing by each vessel in every EEZ and on the high seas in a given year. To isolate only valid fishing activity, we apply a number of filters:

- The fishing activity must have occurred during a valid segment as identified by GFW;
- The fishing activity must have occurred during a segment with more than 10 positions that was not concurrently overlapping with another, longer, segment for the same MMSI;
- The vessel performing the fishing activity must appear on the list of "good" fishing vessels created previously.

We then sum the total number of hours each vessel on our list of "good" fishing vessels spent fishing in each EEZ, or in each FAO region (if the fishing activity occurred on the high seas). Hereafter, the areas in which vessels fish may be referred to EEZs and/or FAO regions, with the understanding that FAO regions are only used to define fishing areas on the high seas. For fishing activity that occurred within an EEZ, we

also determine whether this activity occurred within the territorial waters of the coastal state, defined as being within 12 nautical miles of land.

Eight characteristics are extracted for each vessel: flag state, vessel class, total length (m), gross tonnage (gt), engine power (kW), ship name, call sign, and IMO number. We then calculate fishing effort in units of fishing kilowatt-hours (kWh), by multiplying the hours spent fishing by the engine power of the vessel. Expressing fishing effort in kWh (as opposed to just hours) allows for a more comparable metric of fishing effort across vessels with different gear types and/or sizes. The use of kWh (or kW days) as a globally standardized measure of effort has been used by many studies in the context of fisheries [16, 39-40].

GFW relies upon three different methods to assign vessel class, total length, gross tonnage, and engine power [16]. First, self-reported characteristics ("likely") are those reported by fishing vessels with AIS. However, the identity characteristics broadcast by a vessel's AIS must be manually entered, so vessels engaging in IUU fishing may choose to alter their identity to avoid detection, or there is potential for human error. Second, "known" characteristics are those that appear on vessel registries (such as the EU's vessel registry or the Consolidated List of Authorized Vessels). Third, GFW used data on 45,441 marine vessels listed on official fleet registries to train a CNN model to identify vessel characteristics. This model can use the behavior of vessels (as broadcast by AIS) to identify six classes of fishing vessels and six classes of non-fishing vessels with 95% accuracy and can predict vessel length, engine power, and gross tonnage. "Inferred" fishing vessels are those identified using the CNN. Whenever possible, known designations are considered first, followed by likely designations, and then inferred, to assign the best characteristics to each vessel. The flag state, ship name, call sign, and IMO number we use are those most frequently broadcast by the vessel.

In some cases, there is no flag state broadcast by a vessel. For these vessels, we assume the flag state to be the same as the coastal state in whose waters the vessel spent the most time fishing. This method was used to assign flag states to 474 vessels. There were also 75 vessels with an unknown flag state in our database that only fished on the high seas which were removed.

In total, the GFW-derived database of fishing effort (2018) and vessel characteristics that is used in the SubsidyExplorer toolkit includes information from 70,586 unique vessels. Breakdown of these vessels by gear type is shown in Fig. C, and breakdown by flag state and gear type is shown in Fig. D.

2.3.3. Allocating catches, landed value, and subsidies

In order to estimate the catches, revenue, and subsidies associated with each of the vessels in our fishing effort database, we first calculate catch, revenue, and subsidy rates by flag state and FAO region. These rates—expressed in terms of tonnes/kWh, USD/kWh, and USD/kWh respectively—are then used to proportionally allocate catches, estimated landed value, and subsidies based on the total fishing effort expended (in kWh) by each vessel (see methods for allocating subsidies from [31]). The main text includes an example of how this process works.

For catches and landed value, we calculate rates and perform allocations by flag state and FAO region. In cases where we do not have matching catch and effort data for a given flag state-FAO region pairing, we

instead calculate rates and perform allocations only by flag state. For subsidies, we calculate rates and perform allocations only by flag state. Additionally, we only use the proportion of total subsidies reported by Sumaila et al. [6] associated with industrial fisheries in the calculation of subsidy rates to be applied to vessels in the GFW dataset. The proportions of subsidies associated with industrial fisheries for each country were obtained by subtracting out subsidies for small-scale fisheries as determined by Schuhbauer et al. [38].

Note: A key limitation regarding our approach to estimating subsidy allocations relates to flags of convenience. Many states choose to register their fishing vessels in locations where the costs to do so are lower and/or regulations are less stringent. These states are often referred to as “flags of convenience”. In reality, vessels flying a flag of convenience are usually subsidized by the state from which the vessel’s owner (or operator) originates, which our approach cannot capture. There is very little data globally on vessel ownership, making such attributions for the purposes of subsidy allocation untenable. We recognize that the inability to capture flags of convenience is a limitation of our analysis, but did not feel that it was one we could remedy with defensible assumptions given existing data. A definitive list of states that should be considered flags of convenience does not exist, particularly not one agreed upon by all WTO Members. For those that are interested in which states represented in our data might be considered to be flags of convenience to some parties, the International Transport Workers’ Federation has created their own list of such states.

2.3.4. Converting subsidies to “effective” subsidies

The OECD has undertaken research into the relative effects of different types of fisheries subsidies on fishing effort, harvests, fleet capacity, fishers’ income, and stock size [32, 33]. The idea being that one dollar of one type of subsidy may not have the same effect on fishing effort as one dollar of another type of subsidy. Overall, these studies have found that payments made to the fishing industry based on variable input use (e.g., fuel, bait), have the greatest effect on fishing effort and stock size [32, 33]. Our model of the relationship between fisheries subsidies and fishing effort does not incorporate different relationships for different types of subsidies. Thus, we leverage these findings to convert the values of subsidies estimated for each of our vessels to “effective” subsidies.

We convert the absolute values of different types of fisheries subsidies being provided to industrial fisheries [5, 6] into “effective” subsidy values for each vessel in the GFW dataset. The OECD’s work on the relative effects of fishery supports considers six types of payments: inputs, outputs, fuel, income, capital, and vessels. Based upon the definitions provided for each category of programs [21], we first identified the most closely related subsidy types as defined by Sumaila et al. [20] to each category (Table H).

We then applied the relative effects of the six categories of programs identified by the OECD to the estimates of subsidies provided to industrial fisheries applied to our vessel list [5, 6]. The OECD measured the normalized effect of each policy type, such that the policy type with the largest impact is given a value of “1”, and all others are expressed as a proportion of that effect. We use these proportions to calculate the effective monetary values of different subsidy types. For example, if a vessel receives $100 in subsidies of a type that has an effect on fishing effort that is 40% of the maximum, then we consider that vessel to receive $40 in effective subsidies. The relative impacts of the six policy categories

assessed by the OECD used to calculate effective subsidies are given in Table I. Only the relative effects of different policy types on fishing effort in an open-access scenario were used to calculate effective subsidies. When a subsidy type (as defined in [22]) could be considered to match multiple OECD policy types, the median relative effect on fishing effort for that subsidy type was used.

2.4. Fisheries Management

The causal relationship between certain types of fishery subsidies and overfishing (or overcapacity) likely depends on the effectiveness of the management system in place in the fishery. For example, subsidies that reduce fishing costs are less likely to have an effect in fisheries managed with quotas as compared to those managed with input restrictions [34]. We therefore assign management scores to each of our vessels, such that they can later be classified into different management tiers that might respond differently to subsidy reforms. Classifying or scoring fisheries management systems is undoubtedly challenging and contentious considering the wide variety of approaches used to manage fisheries. Additionally, data on the effectiveness of fisheries management is limited. We therefore consider two different methods of assigning management scores to each of the vessels in our effort database: a survey-derived comparable metric of fisheries management systems, and F/F_{MSY}.

Stock status (e.g., B/B_{MSY}) is often used as a proxy for fisheries management because of the correlation that may exist between the two [e.g., 41]. The theory behind this is simple: the biomass of a fish stock depends on fishing pressure, and management is often instrumental to control fishing pressure. Nonetheless, harvest is not the only factor affecting biomass. Indeed, it has been shown that recruitment (which may not be completely dependent on abundance) may be the biggest predictor of stock size, and the timing of when exploitation began in the fishery is also likely to play a role. Hilborn et al. [41] specifically investigated the connections between stock biomass and fishing pressure, and the corresponding connections to management. They find that many of the worlds' assessed stocks (e.g., those in the RAMLDB) display a similar trend--fishing pressure increased and biomass decreased until 1995 (on average), when fishing pressure started to decrease. Many of these stocks have seen increasing trends in biomass since 2005 (on average), but 19% of the stocks they surveyed still were considered to be "overfished" ($B < B_{MSY}$) even though fishing mortality was controlled at or below the optimal level. Though a correlation certainly exists between B/B_{MSY} and the strength of fisheries management, we chose not to use B/B_{MSY} (or other comparable biological metrics like trophic level, see [42]) to assign management scores. Our primary objective by incorporating management scores is to restrict changes in fishing pressure, rather than to represent the amount of fish in the water available to exploit.

The two different methods of assigning management scores are described in more detail in the subsequent sections. Assignment of management indicators is performed and detailed in .../scripts/03_management.Rmd in the project repository.

2.4.1. FMI scores

Melnychuk et al. [14] have already created a comparable metric of fisheries management systems, and we use this as one potential indicator of management effectiveness. Given that the FMI scores calculated in this study relate to the behavior of the management system, we assume these to be applicable to the administering countries of each fishing area (as opposed to the flag state of each vessel). We assign the

weighted FMI indicators from Melnychuk et al. [14] to each of the vessel-EEZ/FAO region pairings in our effort database based upon the location in which the fishing effort occurred. Where an FMI score is not available for the administering state of an area, we instead assign a FMI score using the following hierarchy:

- FMI score for the sovereign state of the administering state
- Mean FMI score for all EU countries [EU countries and territories only]
- Mean regional FMI score
- Mean regional FMI score associated with the sovereign state
- Mean continental FMI score
- Minimum FMI score from Melnychuk et al. [14] [high seas only]

FMI scores applied to each fishing area using this method range from 0.21 to 0.92 (Fig. E).

2.4.2. Fishing mortality by FAO region

Given that the method described in the previous section assumes that the strength of management is more closely related to the actions of the administering country than to the behavior of the vessels fishing there, we next consider fishing mortality as an indicator of the strength of fisheries management. Fishing mortality measures the exploitation rate on fish stocks as a component of stock status and are often used to make comparisons across different stocks or areas. We calculate the median, mean, and weighted mean fishing mortality rates (F/F_{MSY}) by FAO region (Fig. F) for all stocks in the database constructed by Costello et al. [13]. This data source was chosen for these calculations to try to better represent all stocks (as opposed to just the formally assessed stocks in the RAMLDB). The weighted mean calculation uses the maximum sustainable yield (MSY) of each stock to weight F/F_{MSY}. These values are then applied to each of the vessel-EEZ/FAO region pairings in our effort database based upon the FAO region in which the fishing effort occurred.

3. Bioeconomic Model

The bioeconomic model used in the SubsidyExplorer toolkit to project the outcomes of different subsidy reform policies is similar to those used in the "Sunken Billions" reports [35, 36] to estimate rent losses for the global marine fishery. Our model assumes that global fisheries can be modeled as a single fish stock, represented by an aggregate growth function. A detailed description of the model is provided in the main text. Additionally, we assume that the global fishing industry can be represented by the four discrete fishing fleets mentioned in the previous section—two directly affected by the subsidy reform policies ("affected"), and the other two not ("unaffected"). The relative sizes of the four fleets vary depending on the selected policy, as described in the main text.

3.1. Model inspiration and general considerations

In addition to deriving inspiration from the two Sunken Billions reports [35,36], the model underlying the SubsidyExplorer toolkit is based on those used in other academic works [13,37,43-44]. Here we briefly describe the main features of some of those models, and explain key similarities and differences between them and the model underlying the SubsidyExplorer tool.

3.1.1. Descriptions of similar models

The original Sunken Billions model [35] treats global fisheries as a single fish stock with an aggregate biomass growth function. The global fishing industry (e.g., all fishing fleets) is also represented as an aggregate, by a single profit function. That profit function is made up of a harvest function that relates harvest to fishing effort and biomass, and a cost function that relates fishing cost to fishing effort. For the purposes of this model, maximum sustainable rents (MSR) are identical to profits, and are thus obtained at the fishing level corresponding to the maximum economic yield (MEY). This model assumes that biological overfishing is reversible in the long run, but it does not incorporate any costs associated with restoring the global fishery (e.g., management costs). This model considers two forms of a biological growth function (logistic and Fox). Harvest is a function of catchability, effort, and biomass, where a schooling parameter affects the ease with which biomass can be captured. Revenues are given by price times harvest, and costs include both marginal variable costs (applied to effort) and fixed costs. Ultimately, the model aims to estimate fishery rents, which are not necessarily maximized at the effort level that maximizes profits. For the purposes of their analysis they consider rents to be equal to the difference between equilibrium revenues and variable costs.

The model used in the second Sunken Billions report [36] makes some changes to the previous model, but shares many of the same characteristics. This model also treats global fisheries as an aggregate stock and characterizes the economics of the global fisheries in an aggregate way. Though this model does incorporate demand-side factors to a very limited extent, the authors of the study make note that it should not be used to predict seafood market outcomes since it primarily focuses on production-side dynamics.

We also drew inspiration from simple bioeconomic models used in academic works, including those from Costello et al. [13], Cabral et al. [37], Villaseñor-Derbez et al. [43], and Costello & Granger [44]. There is much overlap between these models, so for brevity, consider the model used by Costello et al. [13]. They use a simple bioeconomic projection model to forecast the trajectories of fisheries under different management regimes. Though they run this model for individual fisheries, this model incorporates a discrete biomass growth function, a harvest function, a profit function, and a downward-sloping demand function that determines fish price.

3.1.2. Similarities and differences to the SubsidyExplorer model

A key similarity between the SubsidyExplorer model and all of the models described in the previous section is that fish stocks and fishing fleets are intentionally simplified (and treated as aggregates) to keep the number of parameters that must be estimated to a manageable number (and to therefore keep the assumptions that need to be made to estimate those parameters to a minimum). As was done in these other analyses, we also aim to estimate the unknown model parameters using the best available empirical data on global fisheries.

The SubsidyExplorer model differs from the original Sunken Billions model in the form of the biological growth function it uses (Pella-Tomlinson vs. logistic (Schaefer-type) and Fox models). All three forms have been commonly used in fisheries assessments, though the Pella-Tomlinson model is generally considered to be an improvement on the other two (and it is able to incorporate both the Schaefer and Fox

forms as special cases). The more recent Sunken Billions model also uses the Pella-Tomlinson form, as do the bioeconomic models used by Costello et al. [13] and Cabral et al. [37].

Both Sunken Billions models employ a "schooling" parameter in the harvest function that dictates fishing efficiency. The SubsidyExplorer model instead follows the approach of Costello et al. [13] (as well as [37, 43-44]), and places an exponent on effort in the cost function to make additional units of effort increasingly costly to apply. Both essentially serve the same purpose by assuming that it's harder/more expensive to catch the last fish/fish the last day as compared to the first. Both Sunken Billions models also include fixed costs in the specification of their cost functions, though the more recent work makes note that these are not included for the purposes of modeling (and are instead incorporated into the variable cost parameter, representing annualized fixed costs), which is consistent with how the SubsidyExplorer model treats costs. Though separating these two types of costs is important for financial accounting, it is not for a long-term economic model (like those discussed here).

Similar to the more recent Sunken Billions model, the SubsidyExplorer model does not explicitly characterize or incorporate management regimes into the fishery (e.g., by way of fixed effort or catch limits), unlike the approach taken by Costello & Grainger [44]. Both the SubsidyExplorer and Sunken Billions models characterize the level of fishing by variable aggregate fishing efforts (a single fleet in Sunken Billions, four fleets in our model), but they are not specifically concerned with how those fishing efforts were determined. Both models can, however, be used to explore the pace or rate at which effort may change (which would in reality be linked to the fishery's governance structure). The Sunken Billions model treats fishing effort as being exogenous (since their primary question relates to what could happen given specified metrics of fishery sustainability), while the SubsidyExplorer model allows it to be variable in response to profits (as do the models in [13, 37]).

Both the SubsidyExplorer model and the more recent Sunken Billions model also incorporate simple demand-side factors by way of a price function (as does the model in [13]). In the Sunken Billions model, fish price is a function of biomass (reflecting the reality that when fish stocks are larger, landings often include larger individuals and/or more valuable species, both of which fetch higher prices). The SubsidyExplorer model instead assumes fish price to be a function of harvest directly.

In general, the parameterization of the SubsidyExplorer model (discussed in the next section) was most influenced by similar assumptions made in the more recent Sunken Billions work [35] and by Costello et al. [13].

3.1.3. Model aggregation: Why only global and regional?

We have been asked many times throughout the course of this analysis whether we could provide country-level results for subsidy reforms. The decision not to (even try) to do so was made very intentionally and early on. The main advantage that comes from using the type of model that is in the SubsidyExplorer toolkit is that it has very few parameters that need to be estimated, limiting the number of assumptions that must be made. Engaging in a country-level (or even higher-resolution) analysis would eliminate this benefit, and would likely necessitate better data than we had available (particularly pertaining to fisheries subsidies). The more recent Sunken Billions report [36] addresses this topic at length with regard to the regional analysis that was undertaken as part of that work. We chose to limit the

disaggregation of our analysis to a regional level for the same reasons, and note that the uncertainty around our regional results is likely higher than with our global results.

There are two key reasons for this decision, both of which would be exacerbated by trying to disaggregate further (e.g., to a country-level). First, regional fisheries data tends to be less available and reliable than global data [36]. Indeed, the process of aggregating data to the global level smooths out many types of errors that may exist at a more disaggregated level (ultimately why using the mean or median to represent a population is often effective). Second, fish stocks do not pay attention to political boundaries. This is especially problematic at a country-level, as the data on the health of stocks are not generally organized at this level. This obstacle becomes less problematic at the regional level as many fish stocks do stay roughly contained within ocean basins, but other data problems may endure.

3.2. Parameterization

3.2.1. Global analysis

As with the models used in the original and revisited Sunken Billions reports [35, 36], the base form of our analysis considers all global marine fisheries as one large fishery. It is a typical aggregate fisheries model based on fisheries economics theory and empirical knowledge. This type of model is a simplified characterization of the global fishery and is not designed to analyze the performance of individual fisheries.

Many parameters used in this model are similar to those utilized in the models from the Sunken Billions reports [35, 36]. The values of fixed parameters used in the global analysis, as well as brief descriptions of their source(s), are provided in Table D. There are also certain parameters used in the model that depend on the relative sizes and compositions of each fleet as discussed in the main text. These variable parameters are calculated at the start of each model run based on the subsidy reform policies selected in the SubsidyExplorer toolkit. Descriptions of the variable parameters in the global analysis are provided in Table E.

3.2.2. Regional analysis

Modeling the world's fisheries as a global stock allows for the easy assessment of different subsidy reform policies in a robust and transparent way due to its simplicity. However, most countries are obviously interested in assessing the effects of different reform policies on a more localized scale. We therefore also include a regional analysis. We divide all global marine fisheries into three regional fisheries in this analysis: Atlantic Ocean, Indian Ocean, and Pacific Ocean (Table F). As with the regional analysis undertaken in the most recent Sunken Billions report [36], we note that the regional results are likely to be less reliable than the global results. Unlike the regional analysis in the Sunken Billions report, we classify regions based on where the fishing takes place, rather than the physical locations of the countries doing the fishing.

We use the same bioeconomic model described above for the regional analysis, with each of the three regions modeled independently. The values of fixed parameters used in the regional analysis are provided in Table G. These parameters were estimated in the same way as the corresponding parameters for the

global analysis (see Table D). The same variable parameters described for the global analysis are also calculated at the start of each model run for the regional analysis based on the subsidy reform policies selected in the SubsidyExplorer toolkit (Table E).

4. Modeling Subsidy Reform Proposals

The subsidy reform proposals submitted by Members to the WTO advocate for placing prohibitions on certain fisheries subsidies. These potential prohibitions are often grouped into three categories: 1) subsidies supporting IUU fishing, 2) subsidies to fishing on overfished stocks, and 3) subsidies contributing to overcapacity or overfishing. Special and differential treatment (S&DT) for all potential prohibitions is sometimes treated as a fourth category (instead of being considered within each category). For each category of prohibitions, different proposals have put forth different methods or approaches to identifying those subsidies that should be prohibited.

To speed up identification of vessels or subsidies that would be likely to trigger different proposed prohibitions within the SubsidyExplorer toolkit, we create a number of additional characteristics to apply to our vessel list. These are based on our interpretation(s) of how existing data could be leveraged to identify prohibited subsidies as proposed to the WTO ("definitions"). For some proposed methods or approaches, there may be more than one possible interpretation, or multiple data sources exist that could be used. We therefore allow for different "definitions" for each category of prohibitions and apply these to our vessel list to create additional vessel characteristics.

The process of creating and assigning additional vessel characteristics based on our reform policy definitions is performed and detailed in *.../scripts/05_subsidy_reform_definitions.Rmd* in the project repository.

The SubsidyExplorer toolkit allows users to select a subsidy reform scenario to model in one of two ways: 1) by selecting one of the pre-made proposals submitted to the WTO, or 2) by creating their own proposal. Both options ultimately utilize the "definitions" discussed in this section to identify vessels that would be likely to be affected by a certain reform policy. The only difference being that for the first case, we have already identified the relevant disciplines and thresholds for each proposal, allowing the user to see the impact of that proposal with one click. In the second case, the user is free to change as many of the settings as they would like.

4.1. Subsidy reform policy "definitions"

4.1.1. Country development status

Most subsidy reform proposals suggest S&DT for developing and least-developed country (LDC) Members, so we assign the development status of the flag state to each vessel in our effort database. The WTO recognizes the LDCs designated by the UN but does not have definitions of "developed" or "developing countries. Members are allowed to self-declare as "developing", though other Members may challenge this designation. For the purposes of modeling, we make the following assumptions about

development status for the purposes of the WTO fishery subsidy negotiations based on past self-declarations made at the WTO:

- **LDCs:** WTO Members and Observers designated as LDCs by the UN are assumed to be LDCs for the purposes of the WTO fishery subsidy negotiations.
- **Developed:** OECD Members except Chile, Mexico, Korea and Turkey are assumed to be developed countries. The following Members with economies in transition as designated by the UN are also considered to be developed countries: Albania, Bosnia and Herzegovina, Montenegro, North Macedonia, Serbia, Armenia, Azerbaijan, Belarus, Georgia, Kazakhstan, the Kyrgyz Republic, the Republic of Moldova, the Russian Federation, Tajikistan, Turkmenistan, Ukraine, and Uzbekistan.
- **Developing:** All other WTO Members and Observers are considered to be developing countries for the purposes of the WTO fishery subsidy negotiations.

Some proposals have also advocated for S&DT for small vulnerable economy (SVE) Members and Observers. We assume the following countries to be SVE Members and Observers: Antigua and Barbuda, Bahamas, Barbados, Belize, Bolivia, Plurinational State of, Cuba, Dominica, Dominican Republic, El Salvador, Ecuador, Fiji, Grenada, Guatemala, Honduras, Jamaica, Mauritania, Nicaragua, Panama, Papua New Guinea, Saint Kitts and Nevis, Saint Lucia, Saint Vincent and the Grenadines, Samoa, Seychelles, Sri Lanka, Tonga, Trinidad and Tobago.

4.1.2. High seas and distant water fishing

Some proposals have introduced the idea of limiting subsidy prohibitions such that they would only apply to vessels fishing on the high seas or in the EEZs of other Members ("distant-water" fishing). This isn't a straightforward classification, as most industrial fishing vessels fish across multiple jurisdictions (i.e., within the EEZ of their own state, on the high seas, and in the EEZ(s) of other coastal states). We therefore determine the fraction of each vessel's total annual fishing effort spent fishing in each area, allowing the user to explore different thresholds relating to these proposed disciplines for high seas or distant water fishing. For example, one could imagine a proposed subsidy prohibition that would only apply to vessels that spend more than 5% of their time fishing on the high seas; alternatively, such as prohibition could only apply to vessels that spend more than 5% of their time fishing on the high seas OR in the EEZs of other coastal states.

The calculation to obtain the fraction of total annual fishing effort spent on the high seas for each vessel is straightforward. Defining what counts as distant-water fishing is somewhat more complicated as many countries have fishing agreements with one another. We apply a similar definition of distant-water fishing to that used by Cabral et al. [37].

When any of the following conditions are met, that fishing activity is NOT considered to be distant water fishing:

- The fishing activity is occurring outside of the jurisdiction of any country (i.e., on the high seas);
- The flag state of the vessel is the same as the administering state of the EEZ in which it is fishing (or one of the administering states in the case of joint regime and disputed areas);

- The flag state of the vessel is a member state of the EU, Norway, Svalbard and Jan Mayen, or Iceland, and the EEZ in which it is fishing is administered by a member state of the EU, Norway, Svalbard and Jan Mayen, or Iceland (e.g., a French flagged vessel fishing in Spain's EEZ);
- The sovereign of the vessel's flag state is a member state of the EU, Norway, Svalbard and Jan Mayen, or Iceland and the EEZ in which it is fishing is administered by a member state of the EU, Norway, Svalbard and Jan Mayen, or Iceland (e.g., a vessel flagged to the Azores fishing in Spain's EEZ).

Our definition of distant water fishing does include "sovereign fishing". Even if the vessel and EEZ in which it is fishing share a sovereign state, we still consider this activity to be distant water fishing (though this does not mean that it is foreign distant water fishing). Therefore, when either of the following conditions are met, we DO consider the activity to be distant water fishing:

- The flag state of the vessel is also the sovereign of the administering entity of the EEZ in which it is fishing (e.g. a US flagged vessel fishing in the EEZ of Palmyra Atoll).
- The sovereign country of the vessel's flag state is also the sovereign of the administering entity of the EEZ in which it is fishing (e.g. a Puerto-Rican flagged vessel fishing in the EEZ of Palmyra Atoll).

4.1.3. Fishing in domestic and territorial waters

Similar to the proposals advocating for limiting subsidy prohibitions to high seas or distant water fishing, other proposals have introduced the idea of offering exceptions for fishing that only occurs within a country's domestic waters (or only within their territorial waters).

For the purposes of making these distinctions, we consider a country's domestic waters to include the entirety of their EEZ, and their territorial waters to include all waters within 12 nautical miles of land.

4.1.4. Fishing in disputed waters

One of the subsidy reform proposals refers to fishing in disputed waters. We use the Maritime Boundaries dataset (v10) from https://Marineregions.org to identify disputed areas to provide one possible interpretation of this proposed prohibition. We consider disputed waters to be any of those classified as disputed in this dataset. We recognize that disputed regions are a sensitive matter, and it is not our intention to convey any opinions regarding the sovereignty of any ocean area.

4.1.5. IUU fishing

Nearly all of the proposed prohibitions for subsidies supporting IUU fishing would require a vessel, operator, or owner to have been identified as having been engaged in IUU fishing. Currently, the most comprehensive lists of such violators come from RFMOs or international organizations such as the International Criminal Police Organisation (INTERPOL). Very few countries maintain lists of vessels having been caught engaging in IUU activities within their waters, and those that do often do not make such determinations publicly available.

Given that there is a great deal of overlap between the IUU lists maintained by RFMOs, we use the Combined IUU Vessel List [15] as one possible method of identifying vessels in our effort database that might trigger an IUU prohibition:

> **IUU #1**: Subsidies for fishing shall be prohibited to any vessel currently listed as having engaged in IUU fishing activities by a RFMO or other international agreement.

We recognize that this method largely underestimates the actual amount of IUU fishing happening worldwide. However, any prohibition agreed upon by the WTO would require a country or international organization or agreement to make an official determination of IUU activity and this is the best source of such information currently available.

Based on the proposals submitted to the WTO, we also offer five other possible definitions of a potential IUU subsidy discipline:

> **IUU #2**: Subsidies for fishing shall be prohibited to any vessel currently listed as having engaged in IUU fishing activities by a coastal Member state.
>
> **IUU #3**: Subsidies for fishing shall be prohibited to any vessel currently listed as having engaged in IUU fishing activities by the flag Member state.
>
> **IUU #4**: Subsidies for fishing shall be prohibited to any vessel currently listed as having engaged in IUU fishing activities by the subsidizing Member state.
>
> **IUU #5**: Subsidies for fishing shall be prohibited to any vessel currently listed as having engaged in IUU fishing activities by a port Member state.
>
> **IUU #6**: Subsidies for fishing shall be prohibited to any vessel currently listed as having engaged in IUU fishing activities by a market Member state.

For these definitions, no data currently exists on a global scale to identify vessels listed as having engaged in IUU fishing activities by coastal, flag, subsidizing Member, port, or market states. Very few states maintain their own IUU lists (e.g., the EU, United States, and Norway), and the very small number of vessels on these lists generally also appear on RFMO lists. Therefore, at present, selecting one of these five options will not return any matching vessels in our effort database.

Even though state IUU lists are extremely limited today, there is reason to believe that adoption of IUU disciplines by the WTO could incentivize more states to maintain and enforce such lists in the future. As a way of thinking about the effects that an IUU discipline could have if more states started maintaining and enforcing their own IUU lists, we offer users the option of making their own assumption about the amount of IUU fishing currently occurring worldwide. This assumption is meant to simulate the % of fishing that might someday be identified and disciplined by state-maintained IUU lists.

If the user chooses to make an assumption about the percentage of total fishing effort that should be considered to be IUU in the SubsidyExplorer toolkit, we assume that the selected percentage is applied equally across all flag states.

4.1.6. Fishing on overfished stocks

Most proposed prohibitions in this category would require two findings: a determination to be made that a particular stock is overfished, and a determination to be made that a particular subsidized vessel/owner/operator is fishing on (or targeting) that stock. In practice, both of these determinations are complicated, and the practice of doing so will depend heavily on the capabilities of the administering state of an area. Nonetheless, we offer the following as a possible interpretation.

The RAMLDB includes status information on approximately 400 fisheries (representing approximately 30-40% of global catches) [12]. Though this database is not globally comprehensive, we consider it to be one method of identifying overfished stocks. We consider two possible definitions of overfished:

> **OA #1**: Subsidies for fishing shall be prohibited if the weighted mean status of all stocks in an area is overfished ($B/B_{MSY} < 1$) as determined by the most recent stock assessments in the RAMLDB.
>
> **OA #2**: Subsidies for fishing shall be prohibited if the weighted mean status of all stocks in an area is overfished ($B/B_{MSY} < 0.8$) as determined by the most recent stock assessments in the RAMLDB.

When applying these definitions, we aggregate stocks by FAO region. We also consider two additional possible definitions of overfished stocks based on the stock status database created by Costello et al. [13]:

> **OA #3**: Subsidies for fishing shall be prohibited if the weighted mean status of all stocks in an area is overfished ($B/B_{MSY} < 1$) as determined by the data-limited assessment done by Costello et al. (2016).
>
> **OA #4**: Subsidies for fishing shall be prohibited if the weighted mean status of all stocks in an area is overfished ($B/B_{MSY} < 0.8$) as determined by the data-limited assessment done by Costello et al. (2016).

4.1.7. Subsidies contributing to overcapacity and overfishing

For the final category of subsidy disciplines, we rely on the definitions of capacity-enhancing subsidies from Sumaila et al. [6]. Using the method described previously to estimate subsidy allocations, we identified vessels that received capacity-enhancing or ambiguous subsidies as those likely to trigger these disciplines.

4.2. Cap and tier considerations

Most of the "definitions" discussed in the previous section are most relevant to subsidy reform proposals that advocate for a complete prohibition of subsidies according to some criteria. Some proposals have put forth an alternative cap-based approach to limiting subsidies. Under such a system, Members would be divided into tiers based on some country-level criteria, and then the total amount of permissible subsidies would be capped based on some other criteria or rule.

For the purposes of modeling such proposals, we must define some additional country-level characteristics.

4.2.1. Subsidy types for capping

Some of the cap-based proposals include a list of "green box" subsidies of which provisioning should be allowed. Such subsidy types should thus not be included in the calculation of a Member's base amount of subsidies that will be used to determine that Member's cap and/or tier. A key assumption underlying this analysis is that the direct relationship between fisheries subsidies and fishing effort is only present for subsidy types that lower the cost of fishing [34]. Therefore, we only include the subsidy types classified as capacity-enhancing or ambiguous by Sumaila et al. [6] when calculating the rate of subsidization for each vessel (and once aggregated, for each fleet). We therefore have implicitly created a "green box" of subsidy types for which we assume that provisioning will continue in the future, but they aren't considered in our analysis because we do not assume them to have a direct effect on fishing effort.

However, we do allow users to decide if all types of capacity-enhancing and ambiguous subsidies should be included in a Member's base for capping under the cap-based approach to limiting subsidies contributing to overcapacity and overfishing. Users are free to include some or all of the ten types of capacity-enhancing and ambiguous subsidies defined by Sumaila et al. [6].

4.2.2. Grouping Members into tiers

One aspect of some cap-based proposals is the separation of Members into different tiers, thus allowing for the different tiers to receive different caps that might better reflect the circumstances of the Members in that tier. Some proposals do not utilize a tiered approach (Members are therefore all included in one tier), but other proposals have put forth various approaches to sort Members into two or three different tiers. We include the following methods of dividing Members into two or three tiers:

- % of global marine capture production;
- % of global capacity-enhancing subsidies;
- Development status.

4.2.3. Methods of setting caps

Once Members have been sorted into tiers (if applicable), most cap-based proposals have advocated for applying some sort of formula to determine each Member's total subsidy cap. We include the following methods of determining Member-specific subsidy caps:

- An absolute amount (USD);
- % of existing capacity-enhancing subsidies;
- % of landed value (from marine capture production);
- % of global average subsidies per fisher multiplied by the total number of fishers;
- The highest of some or all of the previous options;
- No cap.

Different rules can be applied to each of the different tiers (if applicable).

4.3. WTO subsidy reform proposal assumptions

Users of the SubsidyExplorer toolkit are able to select from pre-populated subsidy reform proposals submitted to the WTO. This section briefly describes each of the proposals, and our assumptions for modeling them. Documents are listed in the order they were released to the WTO and is current as of March 2022. This list may not reflect the current selection of pre-populated proposals available on the SubsidyExplorer tool—the selection of proposals under serious consideration by the WTO changes rapidly as do the interpretations of different texts. This list should therefore only be used for reference and we encourage users to defer to the tool for the most up-to-date list of proposals and their assumptions. Any text included in brackets in the proposal summaries below indicates that the values or provisions were only suggestions or included as placeholders in the corresponding text.

4.3.1. CAP - De minimis

Title: Draft text: De minimis
Date: March 21, 2019
Document Number: RD/TN/RL/81
Public? No
Submitting Member(s): Philippines
Summary: This proposal advocates for Member-specific de minimis limits, below which Members may maintain, grant, or provide subsidies. Four criteria are provided to determine de minimis limits: 1) The de minimis limit for developed country Members and developing country Members belonging to the top [10] global marine capture fisheries producers shall be [X]% of the average total landed value of the Member's marine capture fisheries in the period [2016-2018]. 2) For developing country Members not included in 1), the de minimis limit shall be [Y]% plus [10/15]% of the average total landed value in the most recent three year period for which data are available. 3) Developing country Members included in 1) whose large-scale commercial fisheries are responsible for less than [5/10]% of the average total landed value may instead take the de minimis limit determined under 2). 4) The de minimis level for LDC Members shall be [Z]% plus [20/30]% of the average total landed value in the most recent three year period for which data are available.

Illegal, Unreported, and Unregulated

Assumptions:

- None

S&DT:

- N/A

Overfished

Assumptions:

- None

S&DT:

- N/A

Overcapacity and Overfishing

Assumptions:

- None

S&DT:
- N/A

Cap/Tier:
- We assume that provisioning of the following types of subsidies as defined by Sumaila et al. (2019) are allowed to continue irrespective of a Member's total de minimis limit: i) fisher assistance programs; ii) rural fishers' community development program subsidies (both programs relevant to fishing in territorial waters). Therefore each Member's base subsidy level is represented by the total amount of all other capacity-enhancing and ambiguous subsidies estimated by Sumaila et al. (2019).
- We estimate the landed value of marine capture fisheries from the FAO Global Capture Production Database.
- We assume the de minimis limit for developed and developing country members belonging to the top 10 marine capture fisheries producers to be 10% of the average total landed value of the Member's marine capture fisheries between 2016-2018. For all other developing country Members, we assume the de minimis limit to be 20% of the average total landed value between 2016-2018. For LDC Members, we assume the de minimis limit to be 30% of the average total landed value between 2016-2018.

4.3.2. OFOC - Negative effects (Option A)

Title: Draft text on overfishing and overcapacity
Date: April 18, 2019
Document Number: RD/TN/RL/79/Rev.1
Public? No
Submitting Member(s): New Zealand and Iceland
Summary: This proposal advocates for prohibiting subsidies that reduce the operational or capital costs of fishing when one or more of the stocks in the relevant fishery or fisheries being targeted are either 1) being fished with a measure of fishing capacity that is greater than would be required to maintain the stock(s) at a level that would maintain [MSY] or 2) being fished at a rate that is contributing to a decline in the stock(s) below a level that would maintain [MSY]. If the subsidizing Member can demonstrate that it has other policies in place that effectively ensure the stocks(s) are maintained at or above a level required to maintain [MSY], a subsidy that would otherwise be prohibited by either of the above conditions shall be allowed.

Illegal, Unreported, and Unregulated

Assumptions:
- None

S&DT:
- N/A

Overfished

Assumptions:
- None

S&DT:
- N/A

Overcapacity and Overfishing

Assumptions:

- There is uncertainty regarding the status of many fish stocks.
- For the purposes of modeling this proposal, we take the same approach as that used for overfished stocks. We assume that subsidies to fishing on stocks identified as overfished (B/Bmsy < 1) in the RAM Legacy Stock Assessment Database are prohibited.
- The proportion of stocks identified as overfished in the RAM Legacy Stock Assessment Database likely underestimates the actual proportion of stocks that are overfished globally, but it is unclear whether enough evidence would exist to trigger this prohibition for stocks not included in this database.
- This proposal is ambiguous about which reference point might be used to make an overfished determination, and we therefore note that this is only one possible interpretation of this text.
- It is not possible to simulate whether Members could demonstrate that they have policies in place that would negate this prohibition.

S&DT:

- None considered.

Cap/Tier:

- No

4.3.3. CAP - Optional criteria

Title: A cap-based approach to address certain fisheries subsidies that contribute to overcapacity and overfishing
Date: June 4, 2019
Document Number: TN/RL/GEN/199
Public? Yes
Submitting Member(s): China
Summary: This proposal advocates for Member-specific subsidy caps (expressed in monetary terms). Subsidies in excess of a Member's cap would be prohibited. Members may choose from one of three approaches to calculate their subsidy cap: 1) [X]% of the Member's average subsidy base for capping during the base period; 2) [Y]% of the average landed value of the Member's total wild marine capture during the base period; 3) [Z]% of the global average base for capping per fisherman multiplied by the Member's total number of fishermen during the base period. This proposal also advocates that the following four categories of "green-box" subsidies shall not be subject to the Member's capping commitments: 1) government service and management programs; 2) programs to protect fisheries resources or rebuild stocks; 3) programs to reduce fishing efforts or fishing capacity; 4) programs that rebuttably presumed not to contribute to overcapacity or overfishing.

Illegal, Unreported, and Unregulated

Assumptions:

- None

S&DT:

- N/A

Overfished

Assumptions:

- None

S&DT:
- N/A

Overcapacity and Overfishing

Assumptions:
- None

S&DT:
- N/A

Cap/Tier:
- We assume each Member's cap is equal to whichever of the three proposed approaches for calculating their cap yields the largest amount.
- Caps based on the first approach are calculated as 55% of each Member's base for capping; caps based on the second approach are calculated as 2% of each Member's estimated landed value calculated from the FAO Global Marine Capture Production Database (annual average between 2016-2018); caps based on the third approach are calculated as 55% of the global average subsidies per fisher (capacity-enhancing and ambiguous) multiplied by each Member's most recently reported total number of fishers from the FAO Yearbook of Fishery and Aquaculture Statistics (2017). If data are not available to calculate a Member's cap for a certain approach, that approach is disregarded.
- We assume that provisioning of the following types of subsidies as defined by Sumaila et al. (2019) are allowed to continue irrespective of a Member's total cap: i) subsidies for fishing access agreements; ii) fisher assistance programs; iii) vessel buyback programs; iv) rural fishers' community development programs. Therefore each Member's base for capping is represented by the total amount of all other capacity-enhancing and ambiguous subsidies estimated by Sumaila et al. (2019).
- LDCs are exempted from the capping and reduction commitments.

4.3.4. Overfished - Negative effects (Option A)

Title: Draft text on overfished stocks
Date: June 5, 2019
Document Number: RD/TN/RL/77/Rev.2
Public? No
Submitting Member(s): Australia
Summary: This proposal presents a draft text for a prohibition on subsidies for fishing on overfished stocks. It is very similar to some of the draft text provided in RD/TN/RL/119, and advocates for a prohibition on subsidies for fishing on stocks recognized as overfished by the Member under whose jurisdiction the fishing is taking place or by the relevant RFMO/A. Such subsidies shall be prohibited if they are found to be having a negative effect on the stock, defined as any of the following: 1) a lack of recovery of the fishery; 2) continuation of depletion of fish stock levels; 3) continuation of excess levels of fishing.

Illegal, Unreported, and Unregulated

Assumptions:
- None

S&DT:
- N/A

Overfished

Assumptions:
- There is uncertainty regarding the status of many fish stocks.
- For the purposes of modeling this proposal, we assume that subsidies to fishing on stocks identified as overfished (B/Bmsy < 0.8) in the RAM Legacy Stock Assessment Database are prohibited. The proportion of stocks identified as overfished in the RAM Legacy Stock Assessment Database likely underestimates the actual proportion of stocks that are overfished globally, but it is unclear whether enough evidence would exist to trigger this prohibition for stocks not included in this database.
- This proposal is ambiguous about which reference point might be used to make an overfished determination, and we therefore note that this is only one possible interpretation of this text. Additionally, as written, this text requires that there be a causal negative effect on the stock as a result of the subsidy. This is difficult to establish given existing data on subsidy provisioning and stock status, and is not possible to model, but we have chosen a more conservative reference point in regards to this condition.

S&DT:
- None considered.

Overcapacity and Overfishing

Assumptions:
- None

S&DT:
- N/A

Cap/Tier:
- No

4.3.5. IUU (Option A)

Title: Draft text on IUU fisheries subsidies
Date: June 12, 2019
Document Number: RD/TN/RL/87
Public? No
Submitting Member(s): Argentina, Colombia, Costa Rica, Panama, Peru, Uruguay, Canada, Iceland, New Zealand, and the United States
Summary: This proposal advocates for a prohibition on subsidies to vessels or operators engaged in IUU fishing. It specifies that IUU determinations could be made by the flag or subsidizing Member states, by RFMO/As in accordance with their rules and procedures and in conformity with international law, or by coastal Member states for activities in waters under their jurisdictions provided that such a determination is made fairly.

Illegal, Unreported, and Unregulated

Assumptions:
- We assume that final determinations are made by existing RFMO/A vessel lists, by flag, subsidizing, and coastal Member states.

- No publicly available data exist for most flag- and subsidizing Members, nor for coastal Members. The possible effects of modeling this proposal is therefore a conservative interpretation of this text. Users are free to explore a more ambitious interpretation by making their own assumptions about the expected IUU-findings.
- No proportionality or the duration of prohibition is considered.

S&DT:
- None considered.

Overfished

Assumptions:
- None

S&DT:
- N/A

Overcapacity and Overfishing

Assumptions:
- None

S&DT:
- N/A

Cap/Tier:
- No

4.3.6. OFOC - ABNJ

Title: Proposed text on fisheries subsidies for fishing in areas beyond national jurisdictions
Date: June 27, 2019
Document Number: RD/TN/RL/91
Public? No
Submitting Member(s): Argentina, Australia, Chile, New Zealand, the United States, and Uruguay
Summary: This proposal advocates for placing prohibitions on a Member's subsidies contingent upon, or tied to actual or anticipated, fishing activities in areas beyond that Member's national jurisdiction.

Illegal, Unreported, and Unregulated

Assumptions:
- None

S&DT:
- N/A

Overfished

Assumptions:
- None

S&DT:
- N/A

Overcapacity and Overfishing

Assumptions:
- We assume that all capacity-enhancing and ambiguous subsidies as defined by Sumaila et al. (2019) are prohibited to vessels fishing in areas beyond that Member's national jurisdiction. We note that this is an ambitious interpretation of this proposal, as the text makes it clear that the fact that a subsidy is granted is not reason enough for that subsidy to be prohibited. However, data does not exist at present to make this determination for subsidy programs globally.
- By default, we consider all capacity-enhancing and ambiguous subsidies provided to vessels spending at least 5% of their total annual effort fishing on the high seas to be prohibited.

S&DT:
- None considered.

Cap/Tier:
- No

4.3.7. OFOC - List approach

Title: Overcapacity and overfishing
Date: July 2, 2019
Document Number: RD/TN/RL/96/Rev.1
Public? No
Submitting Member(s): Jamaica on behalf of the African, Caribbean and Pacific Countries (ACP) Group
Summary: This proposal builds on TN/RL/GEN/192, as reflected in TN/RL/W/274/Rev.6, and advocates for a prohibition on subsidies for capital and operating costs, provided to large-scale industrial fishing. Capital cost subsidies include those for construction, acquisition, modernization, renovation or upgrading of vessels, direct transfers for vessel construction and modernization, purchase of machines and equipment for fishing vessels. Operating cost subsidies include those for fuel, ice, bait, personnel, income support of vessels or operators or the workers they employ, social chargers, insurance, payments based on the price of fish caught, gear, and at-sea support, or operating losses of such vessels or activities. It also considers a list of subsidies that will not be prohibited: fisheries management, permanent cessation, implementation of international agreements, relief measures, measures for health and safety on board, human capital.

Illegal, Unreported, and Unregulated

Assumptions:
- None

S&DT:
- N/A

Overfished

Assumptions:
- None

S&DT:
- N/A

Overcapacity and Overfishing

Assumptions:
- We assume that the following types of subsidies as defined by Sumaila et al. (2019) are

prohibited: i) boat construction and renovation, ii) fuel.

S&DT:
- This prohibition shall not apply to LDC Members. Subsidies provided by developed Members and developing country Members responsible for less than 2% of global marine capture calculated from the FAO Global Marine Capture Production Database (annual average between 2016-2018) for fishing within their own EEZs shall be allowed.

Cap/Tier:
- No

4.3.8. Overfished - Negative effects + rebuttable (Option B)

Title: Overfished discipline
Date: July 2, 2019
Document Number: RD/TN/RL/95
Public? No
Submitting Member(s): Jamaica on behalf of the African, Caribbean and Pacific Countries (ACP) Group
Summary: This proposal presents a draft text for a prohibition on subsidies for fishing on overfished stocks. It builds on TN/RL/GEN/192, as reflected in TN/RL/W/274/Rev.6, and advocates for a prohibition on subsidies to vessels fishing outside of a Member's territorial sea on overfished stocks. It defines overfished stocks as those recognized as such by the Member in whose jurisdiction the fishing is taking place, or by the relevant RFMO/A. It also provides a list of allowed subsidies.

Illegal, Unreported, and Unregulated

Assumptions:
- None

S&DT:
- N/A

Overfished

Assumptions:
- There is uncertainty regarding the status of many fish stocks.
- For the purposes of modeling this proposal, we assume that subsidies to fishing on stocks identified as overfished (B/Bmsy < 0.8) in the RAM Legacy Stock Assessment Database are prohibited. The proportion of stocks identified as overfished in the RAM Legacy Stock Assessment Database likely underestimates the actual proportion of stocks that are overfished globally, but it is unclear whether enough evidence would exist to trigger this prohibition for stocks not included in this database.
- This proposal is ambiguous about which reference points might be used to make an overfished determination, and we therefore note that this is only one very narrow possible interpretation of this text.
- Subsidies for fishing within a Member's territorial sea shall be allowed.

S&DT:
- S&DT in the form of technical assistance and capacity building are considered, but cannot be modeled.

Overcapacity and Overfishing

Assumptions:

- None

S&DT:

- N/A

Cap/Tier:

- No

4.3.9. CAP - Tiers

Title: A cap-based approach to addressing certain fisheries subsidies
Date: July 11, 2019
Document Number: TN/RL/GEN/197/Rev.2
Public? Yes
Submitting Member(s): Argentina, Australia, the United States, and Uruguay
Summary: This proposal advocates for negotiating Member-specific subsidy caps (expressed in monetary terms). Subsidies in excess of a Member's cap would be prohibited. This cap is intended to be supplementary to any prohibitions on subsidies that support IUU, fishing beyond national jurisdiction, and subsidies for fishing that negatively affect overfished stocks. Members would also commit to maintain fisheries management and conservation measures, and without prejudice of the ASCM.

Illegal, Unreported, and Unregulated

Assumptions:

- None

S&DT:

- N/A

Overfished

Assumptions:

- None

S&DT:

- N/A

Overcapacity and Overfishing

Assumptions:

- None

S&DT:

- N/A

Cap/Tier:

- We assume that each Member's base for capping is represented by the total amount of capacity-enhancing and ambiguous subsidies estimated by Sumaila et al. (2019).
- Members are sorted into three tiers based on their marine capture production calculated from the FAO Global Marine Capture Production Database (annual average between 2016-2018).
- Members accounting for 0.7% or more of global marine capture production are in "Tier 1"; Members accounting for 0.05% or more, but less than 0.7%, of global marine capture production are in "Tier 2"; Members accounting for less than 0.05% of global marine capture

production are in "Tier 3".
- This proposal advocates for allowing both Tier 1 and Tier 2 Members to negotiate their own subsidy caps or to accept a default cap of $50 million annually. For the purposes of modeling, we assume that Tier 1 Members receive a subsidy cap equal to 50% of their base for capping; Tier 2 Members receive the default cap of $50 million; Tier 3 Members do not receive a cap.

4.3.10. OFOC - Vessel characteristics

Title: Fishing vessels to be exclusively or mainly targeted in the context of overcapacity and overfishing disciplines
Date: August 7, 2019
Document Number: RD/TN/RL/103
Public? No
Submitting Member(s): Morocco
Summary: This proposal defines the characteristics of vessels that shall be targeted with regards to overcapacity and overfishing disciplines. It advocates that any vessel having at least three of the following six characteristics shall be affected: vessels 1) greater than 24 meters in length; 2) with a gross tonnage of more than 100 GT; 3) with fishing gear towed or hauled by motor driven equipment; 4) propelled by an engine with more than 130 kW; 5) with onboard freezing equipment for storage; 6) owned or operated by a multinational enterprise, a public limited company, or a joint venture.

Illegal, Unreported, and Unregulated

Assumptions:
- None

S&DT:
- N/A

Overfished

Assumptions:
- None

S&DT:
- N/A

Overcapacity and Overfishing

Assumptions:
- We assume that all capacity-enhancing and ambiguous subsidies as defined by Sumaila et al. (2019) are prohibited to vessels satisfying all three of the following characteristics: i) total length of more than 24 meters; ii) gross tonnage of more than 100 GT; iii) engine power of more than 130 kW.
- In general, our underlying dataset does not include many vessels with fishing gears that are not hauled by motor driven equipment. However, we cannot show that this characteristic is satisfied with certainty and thus it is not considered. The other two conditions are also not considered as it is difficult to determine which vessels have onboard freezing equipment, or the characteristics of a vessel's owning or operating company.

S&DT:
- N/A

Cap/Tier:

- No

4.3.11. IUU (Option B)

Title: Possible consolidated vertical negotiating text
Date: September 10, 2019
Document Number: RD/TN/RL/104
Public? No
Submitting Member(s): European Union
Summary: This proposal aims to consolidate all existing texts that have been put forth or discussed with regards to a prohibition on subsidies that contribute to IUU fishing. It contains alternative approaches to crafting such a discipline, as well as alternative versions of relevant definitions.

Illegal, Unreported, and Unregulated

Assumptions:

- We assume that final determinations are made by existing RFMO/A vessel lists, by flag, subsidizing, coastal, port, or market Member states.
- No publicly available data exist for most flag and subsidizing Member states, nor for coastal, port, or market Member states. The possible effects of modeling this proposal is therefore a conservative interpretation of this text. Users are free to explore a more ambitious interpretation by making their own assumptions about the expected IUU-findings.
- No proportionality or the duration of prohibition is considered.

S&DT:

- None considered.

Overfished

Assumptions:

- None

S&DT:

- N/A

Overcapacity and Overfishing

Assumptions:

- None

S&DT:

- N/A

Cap/Tier:

- No

4.3.12. Disputed waters

Title: Proposed solution to concerns on disputed waters
Date: October 21, 2019
Document Number: TN/RL/GEN/202

Public? Yes
Submitting Member(s): Philippines
Summary: This proposal advocates for prohibiting subsidies in disputed waters, unless all parties have jointly notified the WTO of any agreement to subsidize. Members should attempt to reach an agreement through bilateral consultations before going to Dispute Settlement.

Illegal, Unreported, and Unregulated

Assumptions:
- None

S&DT:
- N/A

Overfished

Assumptions:
- None

S&DT:
- N/A

Overcapacity and Overfishing

Assumptions:
- We assume that all capacity-enhancing and ambiguous subsidies as defined by Sumaila et al. (2019) are prohibited in areas classified as "disputed" in the Marine Regions' Maritime Boundaries World EEZ dataset (v10) [46]. We note that this is a very ambitious interpretation of the possible effects of this proposal.
- It is materially impossible to model non-existing agreements to subsidize in disputed areas.
- *Disclaimer: We recognize that disputed areas are a political matter and our interpretation of this text is not meant to imply the expression of any opinion whatsoever concerning the status of any country, territory, city or area or of its authorities, or concerning the delimitation of its frontier or boundaries.*

S&DT:
- None considered.

Cap/Tier:
- No

4.3.13. OFOC - Prohibition + management

Title: Proposed draft text on a prohibition of subsidies contributing to overcapacity and overfishing
Date: November 6, 2019
Document Number: RD/TN/RL/112/Rev.1
Public? No
Submitting Member(s): European Union, Japan, the Republic of Korea, and the Separate Customs Territory of Taiwan, Penghu, Kinmen, and Matsu
Summary: This proposal advocates for a general prohibition of subsidies that contribute to overcapacity and overfishing. However, it allows for subsidies if the subsidizing Member can demonstrate that the stock(s) targeted by the subsidy recipient are managed on the based on the best publicly available scientific evidence taking into account the following elements: 1) scientific stock assessments; 2) legal

institutions for resource management (i.e. vessel registration and fishery permits); 3) specific conservation and management measures for fish stocks (i.e. input, output, and technical controls taking account of fishery status); 4) monitoring, control and surveillance measures. It also provides a list of subsidies that shall be deemed not to be prohibited. This proposal also includes a reference to the precautionary principle to be included in a preamble and notes that prohibitions should not apply to subsistence fishing.

Illegal, Unreported, and Unregulated

Assumptions:
- None

S&DT:
- N/A

Overfished

Assumptions:
- None

S&DT:
- N/A

Overcapacity and Overfishing

Assumptions:
- There is uncertainty regarding the status of many fish stocks.
- For the purposes of modeling this proposal, we use the same approach as that used for overfished stocks. We assume that subsidies to fishing on stocks identified as overfished (B/Bmsy < 1) in the RAM Legacy Stock Assessment Database are prohibited. The proportion of stocks identified as overfished in the RAM Legacy Stock Assessment Database likely underestimates the actual proportion of stocks that are overfished globally, but it is unclear whether enough evidence would exist to trigger this prohibition for stocks not included in this database.
- The proposal is ambiguous about which reference points might be used to make an overfished determination, and we therefore note that this is only one very narrow possible interpretation of this text.
- Subsidies for subsistence fishing are not captured in this tool, so this exemption is not modeled.

S&DT:
- This prohibition shall not apply to subsidies granted by LDCs for fishing in their territorial waters. A transition period is considered but not modeled.

Cap/Tier:
- No

4.3.14. Facilitator's working text - IUU

Title: Elimination of subsidies that contribute to illegal, unreported and unregulated (IUU) fishing - Facilitator's working paper
Date: December 2, 2019
Document Number: RD/TN/RL/113
Public? No
Submitting Member(s): Facilitator's working paper

Summary: This document summarizes the Facilitator's recommendations regarding the elimination of subsidies that contribute to IUU fishing. Key elements: i) making IUU determinations is a right Members have based on agreements outside the WTO; ii) a subsidies prohibition should not imply an obligation related to making IUU determinations; iii) all procedures must have been followed in making the determination, including appeal and review; iv) there is general agreement that determinations by a coastal state, RFMO/A, flag state, and subsidizing Member state should all trigger the prohibition of subsidies.

Illegal, Unreported, and Unregulated

Assumptions:
- We assume that final determinations are made by existing RFMO/A vessel lists, by flag, subsidizing, and coastal Member states.
- No publically available data exist for most flag and subsidizing Member states, nor for coastal Member states. The possible effects of modeling this proposal is therefore a conservative interpretation of this text. Users are free to explore a more ambitious interpretation by making their own assumptions about the expected IUU-findings.
- No proportionality or the duration of prohibition is considered.

S&DT:
- Subsidies provided by developing country Members and LDCs for fishing within their territorial waters shall be allowed. A transition period is considered but not modeled.

Overfished

Assumptions:
- None

S&DT:
- N/A

Overcapacity and Overfishing

Assumptions:
- None

S&DT:
- N/A

Cap/Tier:
- No

4.3.15. Facilitator's working text - OFOC

Title: Overcapacity and overfishing: Facilitator's working paper
Date: December 2, 2019
Document Number: RD/TN/RL/114
Public? No
Submitting Member(s): Facilitator's working paper
Summary: This document presents the current state of the negotiations as they relate to a prohibition on subsidies that contribute to overcapacity and overfishing. A number of different approaches are discussed along with pertaining unresolved questions. This working paper does not contain text suggestions to model but is included here for reference.

4.3.16. Vessel flagging

Title: Prohibiting subsidies to fishing vessels not flying the Member's flag
Date: December 2, 2019
Document Number: TN/RL/GEN/201/Rev.1
Public? Yes
Submitting Member(s): Argentina, Australia, Indonesia, Japan, New Zealand, the United States, and Uruguay
Summary: This proposal advocates for prohibiting subsidies to vessels that are subsidized by a WTO Member, but do not fly the flag of that Member. An underlying assumption of this tool to make modeling possible is that the subsidizing state of a vessel is its flag state. Therefore, we cannot model this proposal but have included it here for reference.

4.3.17. Facilitator's working text - S&DT

Title: Special and differential treatment: Facilitator's working paper
Date: December 3, 2019
Document Number: RD/TN/RL/118
Public? No
Submitting Member(s): Facilitator's working paper
Summary: This document presents the current state of the negotiations as they relate to special and differential treatment. A number of different approaches are discussed along with pertaining unresolved questions. This working paper does not contain text suggestions to model but is included here for reference.

4.3.18. Facilitator's text - Overfished

Title: Overfished stocks: Facilitator's working paper
Date: December 4, 2019
Document Number: RD/TN/RL/119/Rev.1
Public? No
Submitting Member(s): Facilitator's working paper
Summary: This document presents a draft text on subsidies for fishing overfished stocks. It aims to consolidate possible alternative approaches that have been put forth or discussed, and is an attempt to try to focus future discussions. It provides two alternative approaches for crafting such a prohibition: subsidies shall be prohibited i) if there is a lack of recovery of the stock or if there is a continuous reduction in the level of the stock; ii) if they are not implemented in a manner that ensures rebuilding of the stock. It also provides two possible ways of characterizing and determining what constitutes an overfished stock: i) those recognized as such by the Member under whose jurisdiction the fishing is taking place or by the relevant RFMO/A; ii) those for which the mortality from fishing needs to be restricted to allow the stock to rebuild to some reference point.

INTERPRETATION #1 - Objective definition

Illegal, Unreported, and Unregulated

Assumptions:
- None

S&DT:
- N/A

Overfished

Assumptions:
- There is uncertainty regarding the status of many fish stocks.
- For the purposes of modeling this proposal, we assume that subsidies to fishing on stocks identified as overfished (B/Bmsy < 1) in the RAM Legacy Stock Assessment Database are prohibited. The proportion of stocks identified as overfished in the RAM Legacy Stock Assessment Database likely underestimates the actual proportion of stocks that are overfished globally, but it is unclear whether enough evidence would exist to trigger this prohibition for stocks not included in this database.
- The proposal is ambiguous about which reference point might be used to make an overfished determination, and we therefore note that this is only one possible interpretation of this text. Additionally, as written, this text includes two alternative definitions used to identify an overfished stock - this is the more ambitious interpretation of the two (see OFOC - Negative effects (Option A)).

S&DT:
- None considered.

Overcapacity and Overfishing

Assumptions:
- None

S&DT:
- N/A

Cap/Tier:
- No

INTERPRETATION #2 - Relevant authorities

Illegal, Unreported, and Unregulated

Assumptions:
- None

S&DT:
- N/A

Overfished

Assumptions:
- There is uncertainty regarding the status of many fish stocks.
- For the purposes of modeling this proposal, we assume that subsidies to fishing on stocks identified as overfished (B/Bmsy < 0.8) in the RAM Legacy Stock Assessment Database are prohibited. The proportion of stocks identified as overfished in the RAM Legacy Stock Assessment Database likely underestimates the actual proportion of stocks that are overfished globally, but it is unclear whether enough evidence would exist to trigger this prohibition for

stocks not included in this database.
- The proposal is ambiguous about which reference point might be used to make an overfished determination, and we therefore note that this is only one possible interpretation of this text. Additionally, as written, this text includes two alternative definitions used to identify an overfished stock - this is the less ambitious interpretation of the two (see Overfished - Negative effects (Option A)).

S&DT:
- None considered.

Overcapacity and Overfishing

Assumptions:
- None

S&DT:
- N/A

Cap/Tier:
- No

4.3.19. OFOC - Negative effects + ABNJ

Title: Proposed prohibition on subsidies contributing to overcapacity and overfishing
Date: January 13, 2020
Document Number: RD/TN/RL/121
Public? No
Submitting Member(s): Canada
Summary: This proposal advocates for placing prohibitions on any subsidies contributing to overfishing or overcapacity and any subsidies for fishing outside of the jurisdictions of coastal Members or relevant RFMO/As. Subsidies shall be considered to contribute to overfishing or overcapacity if they benefit an entity engaged in fishing on a stock that is being fished at a rate that is greater than that which would allow it to be maintained at a sustainable level.

Illegal, Unreported, and Unregulated

Assumptions:
- None

S&DT:
- N/A

Overfished

Assumptions:
- None

S&DT:
- N/A

Overcapacity and Overfishing

Assumptions:
- There is uncertainty regarding the status of many fish stocks.
- For the purposes of modeling this proposal, we use the same approach as that used for

overfished stocks. We assume that subsidies to fishing on stocks identified as overfished (B/Bmsy < 1) in the RAM Legacy Stock Assessment Database are prohibited. The proportion of stocks identified as overfished in the RAM Legacy Stock Assessment Database likely underestimates the actual proportion of stocks that are overfished globally, but it is unclear whether enough evidence would exist to trigger this prohibition for stocks not included in this database.

- The proposal is ambiguous about which reference point might be used to make an overfished determination, and we therefore note that this is only one possible interpretation of this text.
- As written, this text also places prohibitions on subsidies for fishing outside of the jurisdictions of coastal Members or relevant RFMO/As. Very few areas of the ocean are not under the jurisdiction of a RFMO/A, but it is difficult to determine on a global scale whether vessels are fishing for species governed by those RFMO/As at any given point in time.
- We therefore assume that all capacity-enhancing and ambiguous subsidies as defined by Sumaila et al. (2019) are prohibited to vessels spending at least 5% of their total annual effort fishing on the high seas, outside of the jurisdictions of coastal Members. We note that this is an ambitious interpretation of this prohibition.

S&DT:
- None considered.

Cap/Tier:
- No

4.3.20. IUU (Option C)

Title: IUU fishing
Date: February 4, 2020
Document Number: RD/TN/RL/89/Rev.2
Public? No
Submitting Member(s): Jamaica on behalf of the African, Caribbean and Pacific Countries (ACP) Group
Summary: This proposal builds on text proposal TN/RL/GEN/192, as reflected in TN/RL/W/274/Rev.6. It specifies that IUU determinations could be made by coastal Member states for activities in waters under their jurisdictions with respect to a foreign vessel or operator, or by RFMO/As in areas (and for species) under their jurisdictions. Members are encouraged to refrain from invoking the discipline based on minor infringements.

Illegal, Unreported, and Unregulated

Assumptions:
- Final determinations are made by existing RFMO/A vessel lists, by flag, subsidizing, and coastal Member states.
- No publicly available data exist for most flag and subsidizing Members, nor for coastal Members. The possible effects of modeling this proposal is therefore a conservative interpretation of this text. Users are free to explore a more ambitious interpretation by making their own assumptions about the expected IUU-findings.
- No proportionality or the duration of prohibition is considered.

S&DT:
- A transition period is considered but not modeled.

Overfished

Assumptions:

- None

S&DT:

- N/A

Overcapacity and Overfishing

Assumptions:

- None

S&DT:

- N/A

Cap/Tier:

- No

4.3.21. CAP - Formula

Title: Mechanism for Reductions and Limits of Fisheries Subsidies
Date: February 29, 2020
Document Number: RD/TN/RL/124
Public? No
Submitting Member(s): Brazil
Summary: This proposal aims at establishing a quantitative approach of reductions and limits to fisheries subsidies. The baseline of reductions is to be the annual average monetary value of fisheries subsidies (over the 3 prior years). It specifies cumulative ranges (8 ranges: $0-15 million USD to over $1.2 billion USD) of subsidies in monetary value. Members falling in each range will be subject to a specific percentage reduction in total fisheries subsidies (from 0% to 45% in increments of 5). The total subsidy reduction shall equal the sum of the cuts for each range.

Illegal, Unreported, and Unregulated

Assumptions:

- None

S&DT:

- N/A

Overfished

Assumptions:

- None

S&DT:

- N/A

Overcapacity and Overfishing

Assumptions:

- None

S&DT:

- N/A

Cap/Tier:

- We assume each Member's baseline to be the total magnitude of capacity-enhancing and ambiguous subsidies estimated by Sumaila et al. (2019).
- The formula ranges and percentages of reduction from the proposal are then applied to all Members.

4.3.22. Comprehensive text proposal

Title: Fisheries Subsidies Text Proposal
Date: March 6, 2020
Document Number: RD/TN/RL/125
Public? No
Submitting Member(s): Chad on behalf of the Least Developed Countries (LDC) Group
Summary: This proposal advocates for fisheries subsidies prohibitions across all three categories of proposed disciplines.

Illegal, Unreported, and Unregulated

Assumptions:

- We assume that final determinations are made by existing RFMO/A vessel lists, by flag, subsidizing, and coastal Member states.
- No publicly available data exist for most flag and subsidizing Members, nor for coastal Members. The possible effects of modeling this proposal is therefore a conservative interpretation of this text. Users are free to explore a more ambitious interpretation by making their own assumptions about the expected IUU-findings.
- No proportionality or the duration of prohibition is considered.

S&DT:

- None considered.

Overfished

Assumptions:

- There is uncertainty regarding the status of many fish stocks.
- For the purposes of modeling this proposal, we assume that subsidies to fishing on stocks identified as overfished (B/Bmsy < 1) in the RAM Legacy Stock Assessment Database are prohibited. The proportion of stocks identified as overfished in the RAM Legacy Stock Assessment Database likely underestimates the actual proportion of stocks that are overfished globally, but it is unclear whether enough evidence would exist to trigger this prohibition for stocks not included in this database.
- The proposal is ambiguous about which reference point might be used to make an overfished determination, and we therefore note that this is only one possible interpretation of this text.

S&DT:

- Subsidies for fishing in a Member's territorial waters shall be allowed. A transition period is considered but not modeled.

Overcapacity and Overfishing

Assumptions:

- We assume that all capacity-enhancing and ambiguous subsidies as defined by Sumaila et al. (2019) are prohibited to vessels spending at least 5% of their total annual effort fishing on the high seas, and to vessels greater than 24 m in length or with a tonnage greater than 100 GT or

with an engine power greater than 130 kW (we assume industrial fishing to be defined by the characteristics specified by OFOC – Vessel characteristics).

S&DT:

- This prohibition shall not apply to LDC Members. A transition period is considered but not modeled.

Cap/Tier:

- No

4.3.23. S&DT

Title: Article [X]: Special and differential treatment
Date: March 6, 2020
Document Number: TN/RL/GEN/200/Rev.1
Public? No
Submitting Member(s): India
Summary: This proposal advocates for special and differential treatment for all three classes of proposed disciplines. As written, this proposal does not specify potential prohibitions within each of the three categories, but only the S&DT that should be allowed for developing countries. To model the possible effects of this proposal, we therefore use the prohibition disciplines proposed in the Comprehensive text proposal (excluding any S&DT).

Illegal, Unreported, and Unregulated

Assumptions:

- Final determinations are made by existing RFMO/A vessel lists, by flag, subsidizing, and coastal Member states.
- No publicly available data exist for most flag and subsidizing Members, nor for coastal Members. The possible effects of modeling this proposal is therefore a conservative interpretation of this text. Users are free to explore a more ambitious interpretation by making their own assumptions about the expected IUU-findings.
- No proportionality or the duration of prohibition is considered.

S&DT:

- Subsidies granted by developing and LDC Members shall be allowed for fishing within their own territorial waters and on the high seas. As written, such exemptions might only apply for a transition period of 7 years, but for the purposes of modeling we assume them to apply indefinitely.

Overfished

Assumptions:

- There is uncertainty regarding the status of many fish stocks.
- For the purposes of modeling this proposal, we assume that subsidies to fishing on stocks identified as overfished (B/Bmsy < 1) in the RAM Legacy Stock Assessment Database are prohibited. The proportion of stocks identified as overfished in the RAM Legacy Stock Assessment Database likely underestimates the actual proportion of stocks that are overfished globally, but it is unclear whether enough evidence would exist to trigger this prohibition for stocks not included in this database.
- The proposal is ambiguous about which reference point might be used to make an overfished determination, and we therefore note that this is only one possible interpretation of this text.

S&DT:

- Subsidies granted by developing and LDC Members shall be allowed for fishing within their own territorial waters and within their own EEZs. As written, the EEZ exemption might only apply for a transition period of 2 years, but for the purposes of modeling we assume it to apply indefinitely.

Overcapacity and Overfishing

Assumptions:

- We assume that all capacity-enhancing and ambiguous subsidies as defined by Sumaila et al. (2019) are prohibited to vessels spending at least 5% of their total annual effort fishing on the high seas, and to vessels greater than 24 m in length or with a tonnage greater than 100 GT or with an engine power greater than 130 kW (we assume industrial fishing to be defined by the characteristics specified by OFOC – Vessel characteristics).

S&DT:

- Subsidies granted by LDCs shall be allowed, and subsidies granted by developing countries shall be allowed for fishing in their territorial seas, or for fishing in their EEZs and high seas unless any of the following conditions are met: a) GNI per capita is greater than US $5000 (constant 2015 US$, World Bank) for three consecutive years (2016-2018), b) their share of annual global marine capture exceeds 2% based on published FAO data (2018), c) they engage in distant water fishing, or d) the contribution from Agriculture, Forestry and Fishing to their national GDP (World Bank) is greater than 10% for three consecutive years (2016-2018). For the purposes of identifying Members engaging in distant water fishing here, we only include Members whose vessels fish in FAO Major Fishing Area(s) that are not directly adjacent to that Member's coastline.

Cap/Tier:

- No

4.3.24. CAP - Formula (revised)

Title: Mechanism for Reductions and Limits of Fisheries Subsidies (revised)
Date: October 6, 2020
Document Number: RD/TN/RL/124/Rev.1
Public? No
Submitting Member(s): Brazil
Summary: This proposal aims at establishing a quantitative approach of reductions and limits to maritime fisheries subsidies that is complementary to the main prohibition. The baseline of reductions is to be the annual average monetary value of fisheries subsidies (3 prior years or 1 year -optional) minus exempted subsidies (cessation, stock protection, artisanal fishing in 12nm zone, disaster relief). It specifies cumulative ranges (8 ranges: $0-15 million USD to over $1.2 billion USD) of subsidies in monetary value. Members falling in each range will be subject to a specific percentage reduction in total fisheries subsidies (from 0% to 15% and then to 45% in increments of 5). The total subsidy reduction shall equal the sum of the cuts on each range; the upper ranges carry larger reduction obligations. The proposal contains a default option of $25 million USD in cases where a Member fails to notify to establish the baseline. It also provides for a flexibility for Members with a baseline below $25 million USD that could be uplifted by $5 million USD under certain conditions (timebound, MSY and capture limit).

Illegal, Unreported, and Unregulated

Assumptions:
- None

S&DT:
- N/A

Overfished

Assumptions:
- None

S&DT:
- N/A

Overcapacity and Overfishing

Assumptions:
- None

S&DT:
- N/A

Cap/Tier:
- We assume each Member's baseline to be the total magnitude of capacity-enhancing subsidies estimated by Sumaila et al. (2019).
- The formula ranges and percentages of reduction from the proposal are then applied to all Members.
- The "uplift" allowance for Members demonstrating a need to develop capacity within their own EEZ cannot be modeled here, thus this proposal is functionally equivalent to the earlier version.

4.3.25. Chair's text - Consolidated text (Dec 2020)

Title: Draft consolidated text (Dec 2020)
Date: December 18, 2020
Document Number: RD/TN/RL/126/Rev.2
Public? No
Submitting Member(s): Chair's draft consolidated text
Summary: This Chair's text aims to consolidate all existing proposals that have been put forth or discussed with regards to a prohibition on subsidies. It was intended to provide a starting point for interactive discussion. As written, it provides two possible ways of characterizing and determining what constitutes an overfished stock: i) those recognized as such by the Member under whose jurisdiction the fishing is taking place or by the relevant RFMO/A; ii) those for which the mortality from fishing needs to be restricted to allow the stock to rebuild to some reference point. Additionally, it includes a blank placeholder for inclusion of a cap-based proposal.

INTERPRETATION #1 - Objective definition + No cap

Illegal, Unreported, and Unregulated

Assumptions:
- We assume that final determinations are made by existing RFMO/A vessel lists, by flag,

subsidizing, coastal, and port Member states.
- No publicly available data exist for most flag and subsidizing Members, nor for coastal Members. The possible effects of modeling this proposal is therefore a conservative interpretation of this text. Users are free to explore a more ambitious interpretation by making their own assumptions about the expected IUU-findings.
- No proportionality or the duration of prohibition is considered.

S&DT:
- Subsidies granted by developing and LDC Members shall be allowed for fishing within their own territorial waters.

Overfished

Assumptions:
- There is uncertainty regarding the status of many fish stocks.
- For the purposes of modeling this proposal, we assume that subsidies to fishing on stocks identified as overfished (B/Bmsy < 1) in the RAM Legacy Stock Assessment Database are prohibited. The proportion of stocks identified as overfished in the RAM Legacy Stock Assessment Database likely underestimates the actual proportion of stocks that are overfished globally, but it is unclear whether enough evidence would exist to trigger this prohibition for stocks not included in this database.
- The proposal is ambiguous about which reference point might be used to make an overfished determination, and we therefore note that this is only one possible interpretation of this text. Additionally, as written, this text includes two alternative definitions used to identify an overfished stock - this is the more ambitious interpretation of the two (see OFOC - Negative effects (Option A)).

S&DT:
- Subsidies granted by developing and LDC Members shall be allowed for fishing within their own territorial waters.

Overcapacity and Overfishing

Assumptions:
- We assume that all capacity-enhancing and ambiguous subsidies as defined by Sumaila et al. (2019) are prohibited to vessels fishing in areas beyond that Member's national jurisdiction. We note that this is an ambitious interpretation of this proposal, as the text makes it clear that the fact that a subsidy is granted is not reason enough for that subsidy to be prohibited. However, data does not exist at present to make this determination for subsidy programs globally.
- By default, we consider all capacity-enhancing subsidies provided to vessels spending at least 5% of their total annual effort fishing on the high seas or fishing in the EEZs of other coastal states to be prohibited.

S&DT:
- Subsidies granted by LDCs shall be allowed, and subsidies granted by developing countries shall be allowed for fishing in their territorial seas, or for fishing in their EEZs and high seas unless any of the following conditions are met: a) GNI per capita is greater than US $5000 (constant 2015 US$, World Bank) for three consecutive years (2016-2018), b) their share of annual global marine capture exceeds 2% based on published FAO data (2018), c) they engage in distant water fishing, or d) the contribution from Agriculture, Forestry and Fishing to their national GDP (World Bank) is greater than 10% for three consecutive years (2016-2018). For the purposes of identifying Members engaging in distant water fishing here, we only include Members whose vessels fish in FAO Major Fishing Area(s) that are not directly adjacent to that

Member's coastline.

Cap/Tier:

- No

INTERPRETATION #2 - Relevant authorities + No cap

Illegal, Unreported, and Unregulated

Assumptions:

- We assume that final determinations are made by existing RFMO/A vessel lists, by flag, subsidizing, coastal, and port Member states.
- No publicly available data exist for most flag and subsidizing Members, nor for coastal Members. The possible effects of modeling this proposal is therefore a conservative interpretation of this text. Users are free to explore a more ambitious interpretation by making their own assumptions about the expected IUU-findings.
- No proportionality or the duration of prohibition is considered.

S&DT:

- Subsidies granted by developing and LDC Members shall be allowed for fishing within their own territorial waters.

Overfished

Assumptions:

- There is uncertainty regarding the status of many fish stocks.
- For the purposes of modeling this proposal, we assume that subsidies to fishing on stocks identified as overfished (B/Bmsy < 0.8) in the RAM Legacy Stock Assessment Database are prohibited. The proportion of stocks identified as overfished in the RAM Legacy Stock Assessment Database likely underestimates the actual proportion of stocks that are overfished globally, but it is unclear whether enough evidence would exist to trigger this prohibition for stocks not included in this database.
- The proposal is ambiguous about which reference point might be used to make an overfished determination, and we therefore note that this is only one possible interpretation of this text. Additionally, as written, this text includes two alternative definitions used to identify an overfished stock - this is the less ambitious interpretation of the two (see Overfished - Negative effects (Option A)).

S&DT:

- Subsidies granted by developing and LDC Members shall be allowed for fishing within their own territorial waters.

Overcapacity and Overfishing

Assumptions:

- We assume that all capacity-enhancing and ambiguous subsidies as defined by Sumaila et al. (2019) are prohibited to vessels fishing in areas beyond that Member's national jurisdiction. We note that this is an ambitious interpretation of this proposal, as the text makes it clear that the fact that a subsidy is granted is not reason enough for that subsidy to be prohibited. However, data does not exist at present to make this determination for subsidy programs globally.
- By default, we consider all capacity-enhancing subsidies provided to vessels spending at least 5% of their total annual effort fishing on the high seas or fishing in the EEZs of other coastal states to be prohibited.

S&DT:

- Subsidies granted by LDCs shall be allowed, and subsidies granted by developing countries shall be allowed for fishing in their territorial seas, or for fishing in their EEZs and high seas unless any of the following conditions are met: a) GNI per capita is greater than US $5000 (constant 2015 US$, World Bank) for three consecutive years (2016-2018), b) their share of annual global marine capture exceeds 2% based on published FAO data (2018), c) they engage in distant water fishing, or d) the contribution from Agriculture, Forestry and Fishing to their national GDP (World Bank) is greater than 10% for three consecutive years (2016-2018). For the purposes of identifying Members engaging in distant water fishing here, we only include Members whose vessels fish in FAO Major Fishing Area(s) that are not directly adjacent to that Member's coastline.

Cap/Tier:

- No

INTERPRETATION #3 - Objective definition + CAP - Optional criteria

Same as interpretation #1, plus the following additional cap and tier assumptions:

Cap/Tier:

- We assume each Member's cap is equal to whichever of the three proposed approaches for calculating their cap yields the largest amount.
- Caps based on the first approach are calculated as 55% of each Member's base for capping; caps based on the second approach are calculated as 2% of each Member's estimated landed value calculated from the FAO Global Marine Capture Production Database (annual average between 2016-2018); caps based on the third approach are calculated as 55% of the global average subsidies per fisher (capacity-enhancing and ambiguous) multiplied by each Member's most recently reported total number of fishers from the FAO Yearbook of Fishery and Aquaculture Statistics (2017). If data are not available to calculate a Member's cap for a certain approach, that approach is disregarded.
- We assume that provisioning of the following types of subsidies as defined by Sumaila et al. (2019) are allowed to continue irrespective of a Member's total cap: i) subsidies for fishing access agreements; ii) fisher assistance programs; iii) vessel buyback programs; iv) rural fishers' community development programs. Therefore each Member's base for capping is represented by the total amount of all other capacity-enhancing and ambiguous subsidies estimated by Sumaila et al. (2019).
- LDCs are exempted from the capping and reduction commitments.

INTERPRETATION #4 - Relevant authorities + CAP - Optional criteria

Same as interpretation #2, plus the cap and tier assumptions from interpretation #4.

INTERPRETATION #5 - Objective definition + CAP - Tiers

Same as interpretation #1, plus the following additional cap and tier assumptions:

Cap/Tier:

- We assume that each Member's base for capping is represented by the total amount of capacity-enhancing and ambiguous subsidies estimated by Sumaila et al. (2019).
- Members are sorted into three tiers based on their marine capture production calculated from the FAO Global Marine Capture Production Database (annual average between 2016-2018). Members accounting for 0.7% or more of global marine capture production are in "Tier 1"; Members accounting for 0.05% or more, but less than 0.7%, of global marine capture production are in "Tier 2"; Members accounting for less than 0.05% of global marine capture

production are in "Tier 3".
- This proposal advocates for allowing both Tier 1 and Tier 2 Members to negotiate their own subsidy caps or to accept a default cap of $50 million annually. For the purposes of modeling, we assume that Tier 1 Members receive a subsidy cap equal to 50% of their base for capping; Tier 2 Members receive the default cap of $50 million; Tier 3 Members do not receive a cap.

INTERPRETATION #6 - Relevant authorities + CAP - Tiers

Same as interpretation #2, plus the cap and tier assumptions from interpretation #5.

INTERPRETATION #7 - Objective Definition + CAP - De minimis

Same as interpretation #1, plus the following additional cap and tier assumptions:

Cap/Tier:
- We assume that provisioning of the following types of subsidies as defined by Sumaila et al. (2019) are allowed to continue irrespective of a Member's total de minimis limit: i) fisher assistance programs; ii) rural fishers' community development program subsidies (both programs relevant to fishing in territorial waters). Therefore each Member's base subsidy level is represented by the total amount of all other capacity-enhancing and ambiguous subsidies estimated by Sumaila et al. (2019).
- We estimate the landed value of marine capture fisheries from the FAO Global Capture Production Database.
- We assume the de minimis limit for developed and developing country members belonging to the top 10 marine capture fisheries producers to be 10% of the average total landed value of the Member's marine capture fisheries between 2016-2018. For all other developing country Members, we assume the de minimis limit to be 20% of the average total landed value between 2016-2018. For LDC Members, we assume the de minimis limit to be 30% of the average total landed value between 2016-2018.

INTERPRETATION #8 - Relevant authorities + CAP - De minimis

Same as interpretation #2, plus the cap and tier assumptions from interpretation #7.

INTERPRETATION #9 - Objective definition + CAP - Formula

Same as interpretation #1, plus the following additional cap and tier assumptions:

Cap/Tier:
- We assume each Member's baseline to be the total magnitude of capacity-enhancing and ambiguous subsidies estimated by Sumaila et al. (2019).
- The formula ranges and percentages of reduction from the proposal are then applied to all Members.

INTERPRETATION #10 - Relevant authorities + CAP - Formula

Same as interpretation #2, plus the cap and tier assumptions from interpretation #9.

4.3.26. Various text amendments

Title: Various amendments

Date: February 19, 2021

Document Number: RD/TN/RL/135

Public? No

Submitting Member(s): Cameroon
Summary: This proposal includes amendments to the Chair's text: 1) Article 1.2 - exclusion of artisanal and small-scale fisheries from scope with transparency requirements; 2) Article 3.2.b - determination of flag states in the high seas; 3) sustainability test of Article 5.2 subject to all members approval and for a 3 year period if members demonstrate that stock is biologically sustainable; 4) Article 9 - Revision of the agreement and termination of the agreement. These amendments cannot be modeled in our framework, and this text is only included for reference.

4.3.27. Artisanal - Exemption

Title: Proposal on Artisanal Fisheries
Date: February 22, 2021
Document Number: RD/TN/RL/136
Public? No
Submitting Member(s): Ecuador, Argentina, and Chile
Summary: This proposal excludes subsidies to artisanal fisheries from the prohibitions of Articles 4 and 5. The criteria to consider the exemption is low income, resource poor or livelihood fishing to ensure food security and limited to 12 nautical miles and applicable domestic legislation. To model the possible effects of this proposal, we use the prohibition disciplines proposed in the Chair's text - Consolidated text (Dec 2020) [Relevant authorities + No cap] (excluding any S&DT).

Illegal, Unreported, and Unregulated

Assumptions:

- We assume that final determinations are made by existing RFMO/A vessel lists, by flag, subsidizing, coastal, and port Member states.
- No publicly available data exist for most flag and subsidizing Members, nor for coastal Members. The possible effects of modeling this proposal is therefore a conservative interpretation of this text. Users are free to explore a more ambitious interpretation by making their own assumptions about the expected IUU-findings.
- No proportionality or the duration of prohibition is considered.

S&DT:

- Subsidies granted by developing and LDC Members shall be allowed for fishing within their own territorial waters. A transition period is considered as an alternative but is not modeled here.

Overfished

Assumptions:

- There is uncertainty regarding the status of many fish stocks.
- For the purposes of modeling this proposal, we assume that subsidies to fishing on stocks identified as overfished (B/Bmsy < 0.8) in the RAM Legacy Stock Assessment Database are prohibited. The proportion of stocks identified as overfished in the RAM Legacy Stock Assessment Database likely underestimates the actual proportion of stocks that are overfished globally, but it is unclear whether enough evidence would exist to trigger this prohibition for stocks not included in this database.
- The proposal is ambiguous about which reference point might be used to make an overfished determination, and we therefore note that this is only one possible interpretation of this text. Additionally, as written, this text includes two alternative definitions used to identify an overfished stock - this is the less ambitious interpretation of the two (see Overfished - Negative

effects (Option A)).

S&DT:

- Subsidies granted by all Members shall be allowed for fishing within their own territorial waters.

Overcapacity and Overfishing

Assumptions:

- We assume that all capacity-enhancing and ambiguous subsidies as defined by Sumaila et al. (2019) are prohibited to vessels fishing in areas beyond that Member's national jurisdiction. We note that this is an ambitious interpretation of this proposal, as the text makes it clear that the fact that a subsidy is granted is not reason enough for that subsidy to be prohibited. However, data does not exist at present to make this determination for subsidy programs globally.
- By default, we consider all capacity-enhancing subsidies provided to vessels spending at least 5% of their total annual effort fishing on the high seas or fishing in the EEZs of other coastal states to be prohibited.

S&DT:

- Subsidies granted by LDCs shall be allowed, and subsidies granted by developing countries shall be allowed for fishing in their territorial seas, or for fishing in their EEZs and high seas unless any of the following conditions are met: a) GNI per capita is greater than US $5000 (constant 2015 US$, World Bank) for three consecutive years (2016-2018), b) their share of annual global marine capture exceeds 2% based on published FAO data (2018), c) they engage in distant water fishing, or d) the contribution from Agriculture, Forestry and Fishing to their national GDP (World Bank) is greater than 10% for three consecutive years (2016-2018). For the purposes of identifying Members engaging in distant water fishing here, we only include Members whose vessels fish in FAO Major Fishing Area(s) that are not directly adjacent to that Member's coastline. A transition period is also considered as an alternative but is not modeled here. If a transition period was employed, we assume that LDCs would enjoy a transition period specifically for them.

Cap/Tier:

- No

4.3.28. Chair's text - June 2021

Title: Draft consolidated text (June 2021)
Date: May 11, 2021
Document Number: TN/RL/W/276/Rev.1
Public? Yes
Submitting Member(s): Chair's draft consolidated text
Summary: This Chair's text represents the progress made since the previous consolidated text was released, and to provide some suggested compromises on remaining outstanding issues. As written, it still provides two possible options for S&DT for Article 5 prohibitions. For the purposes of modeling here, we use ALT 1.

INTERPRETATION #1 - Full exemption + S&DT for all developing countries

Illegal, Unreported, and Unregulated

Assumptions:

- We assume that final determinations are made by existing RFMO/A vessel lists, and by flag and coastal Member states.
- No publicly available data exist for most flag or coastal Members. The possible effects of modeling this proposal is therefore a conservative interpretation of this text. Users are free to explore a more ambitious interpretation by making their own assumptions about the expected IUU-findings.
- No proportionality or the duration of prohibition is considered.

S&DT:

- Subsidies granted by developing and LDC Members shall be allowed for fishing within their own territorial waters. As written, this exception might only apply with a transition period, but for the purposes of modeling we assume it to apply indefinitely.

Overfished

Assumptions:

- There is uncertainty regarding the status of many fish stocks.
- For the purposes of modeling this proposal, we assume that subsidies to fishing on stocks identified as overfished (B/Bmsy < 0.8) in the RAM Legacy Stock Assessment Database are prohibited. The proportion of stocks identified as overfished in the RAM Legacy Stock Assessment Database likely underestimates the actual proportion of stocks that are overfished globally, but it is unclear whether enough evidence would exist to trigger this prohibition for stocks not included in this database.
- The proposal is ambiguous about which reference point might be used to make an overfished determination, and we therefore note that this is only one possible interpretation of this text.
- The definition for an overfished stock subsidy prohibition considered here is the less ambitious interpretation considered in the previous version of the Chair's text - Consolidated text (December 2020) (see Overfished - Negative effects (Option A)).

S&DT:

- Subsidies granted by developing and LDC Members shall be allowed for fishing within their own territorial waters. As written, this exception might only apply with a transition period, but for the purposes of modeling we assume it to apply indefinitely.

Overcapacity and Overfishing

Assumptions:

- As written, this text would prohibit all capacity-enhancing and ambiguous subsidies as defined by Sumaila et al. (2019) unless a Member can demonstrate that measures are implemented to maintain stocks at a biologically sustainable level. It is unclear how many Members would be able to satisfy this sustainability criteria, and thus we assume that such an exemption could be acquired by all Members. We note that this is therefore a conservative interpretation of this text.
- We assume that all capacity-enhancing and ambiguous subsidies as defined by Sumaila et al. (2019) are prohibited to vessels fishing in areas beyond that Member's national jurisdiction. By default, we consider all capacity-enhancing subsidies provided to vessels spending at least 5% of their total annual effort fishing on the high seas or fishing in the EEZs of other coastal states to be prohibited.

S&DT:

- Subsidies granted by LDCs and developing countries shall be allowed. As written, such an exemption might only apply with a transition period, but for the purposes of modeling we assume it to apply indefinitely. We note that this is different from the S&DT called for in the text and is included to illustrate the potential effects of S&DT for all developing countries.

Cap/Tier:
- No

INTERPRETATION #2 - Full exemption

Illegal, Unreported, and Unregulated

Assumptions:
- We assume that final determinations are made by existing RFMO/A vessel lists, and by flag and coastal Member states.
- No publicly available data exist for most flag or coastal Members. The possible effects of modeling this proposal is therefore a conservative interpretation of this text. Users are free to explore a more ambitious interpretation by making their own assumptions about the expected IUU-findings.
- No proportionality or the duration of prohibition is considered.

S&DT:
- Subsidies granted by developing and LDC Members shall be allowed for fishing within their own territorial waters. As written, this exception might only apply with a transition period, but for the purposes of modeling we assume it to apply indefinitely.

Overfished

Assumptions:
- There is uncertainty regarding the status of many fish stocks.
- For the purposes of modeling this proposal, we assume that subsidies to fishing on stocks identified as overfished (B/Bmsy < 0.8) in the RAM Legacy Stock Assessment Database are prohibited. The proportion of stocks identified as overfished in the RAM Legacy Stock Assessment Database likely underestimates the actual proportion of stocks that are overfished globally, but it is unclear whether enough evidence would exist to trigger this prohibition for stocks not included in this database.
- The proposal is ambiguous about which reference point might be used to make an overfished determination, and we therefore note that this is only one possible interpretation of this text.
- The definition for an overfished stock subsidy prohibition considered here is the less ambitious interpretation considered in the previous version of the Chair’s text - Consolidated text (December 2020) (see Overfished - Negative effects (Option A)).

S&DT:
- Subsidies granted by developing and LDC Members shall be allowed for fishing within their own territorial waters. As written, this exception might only apply with a transition period, but for the purposes of modeling we assume it to apply indefinitely.

Overcapacity and Overfishing

Assumptions:
- As written, this text would prohibit all capacity-enhancing and ambiguous subsidies as defined by Sumaila et al. (2019) unless a Member can demonstrate that measures are implemented to maintain stocks at a biologically sustainable level. It is unclear how many Members would be able to satisfy this sustainability criteria, and thus we assume that such an exemption could be acquired by all Members. We note that this is therefore a conservative interpretation of this text.
- We assume that all capacity-enhancing and ambiguous subsidies as defined by Sumaila et al. (2019) are prohibited to vessels fishing in areas beyond that Member's national jurisdiction. By default, we consider all capacity-enhancing subsidies provided to vessels spending at least 5%

of their total annual effort fishing on the high seas or fishing in the EEZs of other coastal states to be prohibited.

S&DT:

- Subsidies granted by LDCs shall be allowed, and subsidies granted by developing countries shall be allowed for fishing in their territorial seas, or for fishing in their EEZs and high seas unless any of the following conditions are met: a) GNI per capita is greater than US $5000 (constant 2015 US$, World Bank) for three consecutive years (2016-2018), b) their share of annual global marine capture exceeds 2% based on published FAO data (2018), c) they engage in distant water fishing, or d) the contribution from Agriculture, Forestry and Fishing to their national GDP (World Bank) is greater than 10% for three consecutive years (2016-2018). For the purposes of identifying Members engaging in distant water fishing here, we only include Members whose vessels fish in FAO Major Fishing Area(s) that are not directly adjacent to that Member's coastline. As written, this exemption might only apply with a transition period, but for the purposes of modeling we assume it to apply indefinitely.

Cap/Tier:

- No

INTERPRETATION #3 - Managed exemption

Illegal, Unreported, and Unregulated

Assumptions:

- We assume that final determinations are made by existing RFMO/A vessel lists, and by flag and coastal Member states.
- No publicly available data exist for most flag or coastal Members. The possible effects of modeling this proposal is therefore a conservative interpretation of this text. Users are free to explore a more ambitious interpretation by making their own assumptions about the expected IUU-findings.
- No proportionality or the duration of prohibition is considered.

S&DT:

- Subsidies granted by developing and LDC Members shall be allowed for fishing within their own territorial waters. As written, this exception might only apply with a transition period, but for the purposes of modeling we assume it to apply indefinitely.

Overfished

Assumptions:

- There is uncertainty regarding the status of many fish stocks.
- For the purposes of modeling this proposal, we assume that subsidies to fishing on stocks identified as overfished (B/Bmsy < 0.8) in the RAM Legacy Stock Assessment Database are prohibited. The proportion of stocks identified as overfished in the RAM Legacy Stock Assessment Database likely underestimates the actual proportion of stocks that are overfished globally, but it is unclear whether enough evidence would exist to trigger this prohibition for stocks not included in this database.
- The proposal is ambiguous about which reference point might be used to make an overfished determination, and we therefore note that this is only one possible interpretation of this text.
- The definition for an overfished stock subsidy prohibition considered here is the less ambitious interpretation considered in the previous version of the Chair’s text - Consolidated text (December 2020) (see Overfished - Negative effects (Option A)).

S&DT:

- Subsidies granted by developing and LDC Members shall be allowed for fishing within their own territorial waters. As written, this exception might only apply with a transition period, but for the purposes of modeling we assume it to apply indefinitely.

Overcapacity and Overfishing

Assumptions:

- As written, this text would prohibit all capacity-enhancing and ambiguous subsidies as defined by Sumaila et al. (2019) unless a Member can demonstrate that measures are implemented to maintain stocks at a biologically sustainable level. It is unclear how many Members would be able to satisfy this sustainability criteria, and thus we assume that such an exemption could only be acquired by vessels fulfilling our management criteria. We note that this may still be a conservative interpretation of this text because our management criteria are determined based on the location in which the fishing takes place, as opposed to by the flag- or subsidizing Member state.
- This text would also prohibit subsidies to fishing in areas beyond the subsidizing Member's national jurisdiction. We therefore assume that all capacity-enhancing and ambiguous subsidies as defined by Sumaila et al. (2019) are prohibited to vessels fishing in areas beyond that Member's national jurisdiction. By default, we consider all capacity-enhancing subsidies provided to vessels spending at least 5% of their total annual effort fishing on the high seas or in the EEZs of other coastal states to be prohibited.

S&DT:

- Subsidies granted by LDCs shall be allowed, and subsidies granted by developing countries shall be allowed for fishing in their territorial seas, or for fishing in their EEZs and high seas unless any of the following conditions are met: a) GNI per capita is greater than US $5000 (constant 2015 US$, World Bank) for three consecutive years (2016-2018), b) their share of annual global marine capture exceeds 2% based on published FAO data (2018), c) they engage in distant water fishing, or d) the contribution from Agriculture, Forestry and Fishing to their national GDP (World Bank) is greater than 10% for three consecutive years (2016-2018). For the purposes of identifying Members engaging in distant water fishing here, we only include Members whose vessels fish in FAO Major Fishing Area(s) that are not directly adjacent to that Member's coastline. As written, this exemption might only apply with a transition period, but for the purposes of modeling we assume it to apply indefinitely.

Cap/Tier:

- No

INTERPRETATION #4 - Sustainability scenario

Illegal, Unreported, and Unregulated

Assumptions:

- We assume that final determinations are made by existing RFMO/A vessel lists, and by flag and coastal Member states.
- No publicly available data exist for most flag or coastal Members, so we assume 20% of fishing effort worldwide to be identified as IUU.
- No proportionality or the duration of prohibition is considered.

S&DT:

- None considered.

Overfished

Assumptions:

- There is uncertainty regarding the status of many fish stocks.
- For the purposes of modeling this proposal, we assume that subsidies to fishing on stocks identified as overfished (B/Bmsy < 1) in the RAM Legacy Stock Assessment Database are prohibited. The proportion of stocks identified as overfished in the RAM Legacy Stock Assessment Database likely underestimates the actual proportion of stocks that are overfished globally, but it is unclear whether enough evidence would exist to trigger this prohibition for stocks not included in this database.
- The proposal is ambiguous about which reference point might be used to make an overfished determination, and we therefore note that this is only one possible interpretation of this text.
- The definition for an overfished stock subsidy prohibition considered here is the more ambitious interpretation considered in the previous version of the Chair's text – Consolidated text (December 2020) (see OFOC - Negative effects (Option A)).

S&DT:

- None considered.

Overcapacity and Overfishing

Assumptions:

- We assume that all capacity-enhancing and ambiguous subsidies as defined by Sumaila et al. (2019) are prohibited for all fishing (within Members' own EEZs, on the high seas, and in the EEZs of other coastal states). We note that this is an ambitious interpretation of this proposal as no exemption is provided to demonstrate that measures are implemented to maintain stocks at a biologically sustainable level.
- The specific provisions related to high seas fishing and/or fishing in the EEZs of other coastal states are covered in the first assumption.

S&DT:

- Subsidies granted by developing and LDC Members shall be allowed for fishing within their own territorial waters. This exception might only apply with a transition period, but for the purposes of modeling we assume it to apply indefinitely.

Cap/Tier:

- No

4.3.29. Chair's text - November 2021

Title: Draft Agreement - MC12
Date: November 24, 2021
Document Number: WT/MIN(21)/W/5
Public? Yes
Submitting Member(s): Chair's draft negotiating agreement
Summary: This Chair's text considered Members' comments and views in all discussions based on the previous consolidated draft documents (TN/RL/W/276 and Revs. 1 & 2). This new text aims to capture the progress that Members have made since TN/RL/W/276/Rev.2 was issued. This text also suggests compromises on outstanding issues, meaning that it contains some new language. This text is without prejudice to the position of any Member in respect of any issue.

INTERPRETATION #1 - Full exemption

Illegal, Unreported, and Unregulated

Assumptions:

- We assume that final determinations are made by existing RFMO/A vessel lists, and by flag and coastal Member states.
- No publicly available data exist for most flag or coastal Members. The possible effects of modeling this proposal is therefore a conservative interpretation of this text. Users are free to explore a more ambitious interpretation by making their own assumptions about the expected IUU-findings.
- No proportionality or the duration of prohibition is considered.

S&DT:

- Subsidies granted by developing and LDC Members shall be allowed for low income, resource-poor, or livelihood fishing. For the purposes of modeling, we assume this definition to allow developing and LDC Members to grant subsidies for fishing within their own territorial waters. As written, this exemption would only apply for a transition period of 2 years, but for the purposes of modeling we assume it to apply indefinitely.

Overfished

Assumptions:

- There is uncertainty regarding the status of many fish stocks.
- For the purposes of modeling this proposal, we assume that subsidies to fishing on stocks identified as overfished ($B/B_{msy} < 0.8$) in the RAM Legacy Stock Assessment Database are prohibited. The proportion of stocks identified as overfished in the RAM Legacy Stock Assessment Database likely underestimates the actual proportion of stocks that are overfished globally, but it is unclear whether enough evidence would exist to trigger this prohibition for stocks not included in this database.
- The proposal is ambiguous about which reference point might be used to make an overfished determination, and we therefore note that this is only one possible interpretation of this text.
- The definition for an overfished stock subsidy prohibition considered here is the less ambitious interpretation considered in the previous version of the Chair's text - Consolidated text (December 2020) (see Overfished - Negative effects (Option A)).

S&DT:

- Subsidies granted by developing and LDC Members shall be allowed for low income, resource-poor, or livelihood fishing. For the purposes of modeling, we assume this definition to allow developing and LDC Members to grant subsidies for fishing within their own territorial waters. As written, this exemption would only apply for a transition period of 2 years, but for the purposes of modeling we assume it to apply indefinitely.

Overcapacity and Overfishing

Assumptions:

- As written, this text would prohibit all capacity-enhancing and ambiguous subsidies as defined by Sumaila et al. (2019) unless a Member can demonstrate that measures are implemented to maintain stocks at a biologically sustainable level. It is unclear how many Members would be able to satisfy this sustainability criteria, and thus we assume that such an exemption could be acquired by all Members. We note that this is therefore a conservative interpretation of this text.
- This text would also prohibit subsidies to fishing in areas beyond the subsidizing Member's national jurisdiction, but this prohibition would also be contingent on whether a Member can demonstrate that measures are implemented to maintain stocks at a biologically sustainable level. We apply the same assumption regarding the ability of all Members to acquire this

exemption as it relates to subsidies for fishing in areas beyond the Member's national jurisdiction.

S&DT:

- Subsidies granted by LDCs shall be allowed, and subsidies granted by developing countries shall be allowed for fishing in their territorial seas unless they are responsible for more than 10% of annual global marine capture production. Developing Members responsible for less than 0.7% of global marine capture production will also be allowed to continue providing subsidies for fishing in their EEZs and in the high seas. As written, developing Members not meeting this criteria may also be allowed to provide subsidies for fishing in their EEZs and in the areas of competence of RFMO/As for a transition period, but this is not modeled.

Cap/Tier:

- No

INTERPRETATION #2 - Managed exemption

Illegal, Unreported, and Unregulated

Assumptions:

- We assume that final determinations are made by existing RFMO/A vessel lists, and by flag and coastal Member states.
- No publicly available data exist for most flag or coastal Members. The possible effects of modeling this proposal is therefore a conservative interpretation of this text. Users are free to explore a more ambitious interpretation by making their own assumptions about the expected IUU-findings.
- No proportionality or the duration of prohibition is considered.

S&DT:

- Subsidies granted by developing and LDC Members shall be allowed for low income, resource-poor, or livelihood fishing. For the purposes of modeling, we assume this definition to allow developing and LDC Members to grant subsidies for fishing within their own territorial waters. As written, this exemption would only apply for a transition period of 2 years, but for the purposes of modeling we assume it to apply indefinitely.

Overfished

Assumptions:

- There is uncertainty regarding the status of many fish stocks.
- For the purposes of modeling this proposal, we assume that subsidies to fishing on stocks identified as overfished (B/Bmsy < 0.8) in the RAM Legacy Stock Assessment Database are prohibited. The proportion of stocks identified as overfished in the RAM Legacy Stock Assessment Database likely underestimates the actual proportion of stocks that are overfished globally, but it is unclear whether enough evidence would exist to trigger this prohibition for stocks not included in this database.
- The proposal is ambiguous about which reference point might be used to make an overfished determination, and we therefore note that this is only one possible interpretation of this text.
- The definition for an overfished stock subsidy prohibition considered here is the less ambitious interpretation considered in the previous version of the Chair's text - Consolidated text (December 2020) (see Overfished - Negative effects (Option A)).

S&DT:

- Subsidies granted by developing and LDC Members shall be allowed for low income, resource-poor, or livelihood fishing. For the purposes of modeling, we assume this definition to allow

developing and LDC Members to grant subsidies for fishing within their own territorial waters. As written, this exemption would only apply for a transition period of 2 years, but for the purposes of modeling we assume it to apply indefinitely.

Overcapacity and Overfishing

Assumptions:

- As written, this text would prohibit all capacity-enhancing and ambiguous subsidies as defined by Sumaila et al. (2019) unless a Member can demonstrate that measures are implemented to maintain stocks at a biologically sustainable level. It is unclear how many Members would be able to satisfy this sustainability criteria, and thus we assume that such an exemption could only be acquired by vessels fulfilling our management criteria. We note that this may still be a conservative interpretation of this text because our management criteria are determined based on the location in which the fishing takes place, as opposed to by the flag- or subsidizing Member state.
- This text would also prohibit subsidies to fishing in areas beyond the subsidizing Member's national jurisdiction, but this prohibition would also be contingent on whether a Member can demonstrate that measures are implemented to maintain stocks at a biologically sustainable level. We apply the same assumption regarding the ability of all Members to acquire this exemption as it relates to subsidies for fishing in areas beyond the Member's national jurisdiction. For vessels not fulfilling our management criteria, we assume that all capacity-enhancing and ambiguous subsidies as defined by Sumaila et al. (2019) are prohibited to vessels fishing in areas beyond that Member's national jurisdiction. By default, we consider all capacity-enhancing subsidies provided to vessels spending at least 5% of their total annual effort fishing on the high seas and/or in the EEZs of another coastal state to be prohibited.

S&DT:

- Subsidies granted by LDCs shall be allowed, and subsidies granted by developing countries shall be allowed for fishing in their territorial seas unless they are responsible for more than 10% of annual global marine capture production. Developing Members responsible for less than 0.7% of global marine capture production will also be allowed to continue providing subsidies for fishing in their EEZs and in the high seas. As written, developing Members not meeting this criteria may also be allowed to provide subsidies for fishing in their EEZs and in the areas of competence of RFMO/As for a transition period, but this is not modeled.

Cap/Tier:

- No

INTERPRETATION #3 - Sustainability scenario

Illegal, Unreported, and Unregulated

Assumptions:

- We assume that final determinations are made by existing RFMO/A vessel lists, and by flag and coastal Member states.
- No publicly available data exist for most flag or coastal Members, so we assume 20% of fishing effort worldwide to be identified as IUU.
- No proportionality or the duration of prohibition is considered.

S&DT:

- None considered.

Overfished

Assumptions:

- There is uncertainty regarding the status of many fish stocks.
- For the purposes of modeling this proposal, we assume that subsidies to fishing on stocks identified as overfished (B/Bmsy < 1) in the RAM Legacy Stock Assessment Database are prohibited. The proportion of stocks identified as overfished in the RAM Legacy Stock Assessment Database likely underestimates the actual proportion of stocks that are overfished globally, but it is unclear whether enough evidence would exist to trigger this prohibition for stocks not included in this database.
- The proposal is ambiguous about which reference point might be used to make an overfished determination, and we therefore note that this is only one possible interpretation of this text.
- The definition for an overfished stock subsidy prohibition considered here is the more ambitious interpretation considered in the previous version of the Chair's text – Consolidated text (December 2020) (see OFOC - Negative effects (Option A)).

S&DT:

- None considered.

Overcapacity and Overfishing

Assumptions:

- We assume that all capacity-enhancing and ambiguous subsidies as defined by Sumaila et al. (2019) are prohibited for all fishing (within Members' own EEZs, on the high seas, and in the EEZs of other coastal states). We note that this is an ambitious interpretation of this proposal as no exemption is provided to demonstrate that measures are implemented to maintain stocks at a biologically sustainable level.
- The specific provisions related to high seas fishing and/or fishing in the EEZs of other coastal states are covered in the first assumption.

S&DT:

- Subsidies granted by developing and LDC Members shall be allowed for fishing within their own territorial waters. This exception might only apply with a transition period, but for the purposes of modeling we assume it to apply indefinitely.

Cap/Tier:

- No

INTERPRETATION #4 - Full exemption for domestic only

Illegal, Unreported, and Unregulated

Assumptions:

- We assume that final determinations are made by existing RFMO/A vessel lists, and by flag and coastal Member states.
- No publicly available data exist for most flag or coastal Members. The possible effects of modeling this proposal is therefore a conservative interpretation of this text. Users are free to explore a more ambitious interpretation by making their own assumptions about the expected IUU-findings.
- No proportionality or the duration of prohibition is considered.

S&DT:

- Subsidies granted by developing and LDC Members shall be allowed for low income, resource-poor, or livelihood fishing. For the purposes of modeling, we assume this definition to allow developing and LDC Members to grant subsidies for fishing within their own territorial waters.

As written, this exemption would only apply for a transition period of 2 years, but for the purposes of modeling we assume it to apply indefinitely.

Overfished

Assumptions:

- There is uncertainty regarding the status of many fish stocks.
- For the purposes of modeling this proposal, we assume that subsidies to fishing on stocks identified as overfished (B/Bmsy < 0.8) in the RAM Legacy Stock Assessment Database are prohibited. The proportion of stocks identified as overfished in the RAM Legacy Stock Assessment Database likely underestimates the actual proportion of stocks that are overfished globally, but it is unclear whether enough evidence would exist to trigger this prohibition for stocks not included in this database.
- The proposal is ambiguous about which reference point might be used to make an overfished determination, and we therefore note that this is only one possible interpretation of this text.
- The definition for an overfished stock subsidy prohibition considered here is the less ambitious interpretation considered in the previous version of the Chair's text - Consolidated text (December 2020) (see Overfished - Negative effects (Option A)).

S&DT:

- Subsidies granted by developing and LDC Members shall be allowed for low income, resource-poor, or livelihood fishing. For the purposes of modeling, we assume this definition to allow developing and LDC Members to grant subsidies for fishing within their own territorial waters. As written, this exemption would only apply for a transition period of 2 years, but for the purposes of modeling we assume it to apply indefinitely.

Overcapacity and Overfishing

Assumptions:

- As written, this text would prohibit all capacity-enhancing and ambiguous subsidies as defined by Sumaila et al. (2019) unless a Member can demonstrate that measures are implemented to maintain stocks at a biologically sustainable level. It is unclear how many Members would be able to satisfy this sustainability criteria, and thus we assume that such an exemption could be acquired by all Members. We note that this is therefore a conservative interpretation of this text.
- This text would also prohibit subsidies to fishing in areas beyond the subsidizing Member's national jurisdiction. As written, such a prohibition may not apply if a Member could satisfy the same sustainability criteria, unless the fishing activity falls outside the jurisdiction of a RFMO/A. Very few areas of the ocean are not under the jurisdiction of a RFMO/A, but it is difficult to determine on a global scale whether vessels are fishing for species governed by those RFMO/As at any given point in time. We therefore assume that all capacity-enhancing and ambiguous subsidies as defined by Sumaila et al. (2019) are prohibited to vessels fishing in areas beyond that Member's national jurisdiction. By default, we consider all capacity-enhancing subsidies provided to vessels spending at least 5% of their total annual effort fishing on the high seas or in the EEZs of other coastal states to be prohibited. We note that this may be an ambitious interpretation of the prohibition on subsidies to fishing in areas beyond national jurisdictions.

S&DT:

- Subsidies granted by LDCs shall be allowed, and subsidies granted by developing countries shall be allowed for fishing in their territorial seas unless they are responsible for more than 10% of annual global marine capture production. Developing Members responsible for less than 0.7% of global marine capture production will also be allowed to continue providing subsidies for fishing in their EEZs and in the high seas. As written, developing Members not meeting this

criteria may also be allowed to provide subsidies for fishing in their EEZs and in the areas of competence of RFMO/As for a transition period, but this is not modeled.

Cap/Tier:
- No

INTERPRETATION #5 - Managed exemption for domestic only

Illegal, Unreported, and Unregulated

Assumptions:
- We assume that final determinations are made by existing RFMO/A vessel lists, and by flag and coastal Member states.
- No publicly available data exist for most flag or coastal Members. The possible effects of modeling this proposal is therefore a conservative interpretation of this text. Users are free to explore a more ambitious interpretation by making their own assumptions about the expected IUU-findings.
- No proportionality or the duration of prohibition is considered.

S&DT:
- Subsidies granted by developing and LDC Members shall be allowed for low income, resource-poor, or livelihood fishing. For the purposes of modeling, we assume this definition to allow developing and LDC Members to grant subsidies for fishing within their own territorial waters. As written, this exemption would only apply for a transition period of 2 years, but for the purposes of modeling we assume it to apply indefinitely.

Overfished

Assumptions:
- There is uncertainty regarding the status of many fish stocks.
- For the purposes of modeling this proposal, we assume that subsidies to fishing on stocks identified as overfished ($B/Bmsy < 0.8$) in the RAM Legacy Stock Assessment Database are prohibited. The proportion of stocks identified as overfished in the RAM Legacy Stock Assessment Database likely underestimates the actual proportion of stocks that are overfished globally, but it is unclear whether enough evidence would exist to trigger this prohibition for stocks not included in this database.
- The proposal is ambiguous about which reference point might be used to make an overfished determination, and we therefore note that this is only one possible interpretation of this text.
- The definition for an overfished stock subsidy prohibition considered here is the less ambitious interpretation considered in the previous version of the Chair's text - Consolidated text (December 2020) (see Overfished - Negative effects (Option A)).

S&DT:
- Subsidies granted by developing and LDC Members shall be allowed for low income, resource-poor, or livelihood fishing. For the purposes of modeling, we assume this definition to allow developing and LDC Members to grant subsidies for fishing within their own territorial waters. As written, this exemption would only apply for a transition period of 2 years, but for the purposes of modeling we assume it to apply indefinitely.

Overcapacity and Overfishing

Assumptions:
- As written, this text would prohibit all capacity-enhancing and ambiguous subsidies as defined by Sumaila et al. (2019) unless a Member can demonstrate that measures are implemented to maintain stocks at a biologically sustainable level. It is unclear how many Members would be

able to satisfy this sustainability criteria, and thus we assume that such an exemption could only be acquired by vessels fulfilling our management criteria. We note that this may still be a conservative interpretation of this text because our management criteria are determined based on the location in which the fishing takes place, as opposed to by the flag- or subsidizing Member state.
- This text would also prohibit subsidies to fishing in areas beyond the subsidizing Member's national jurisdiction. As written, such a prohibition may not apply if a Member could satisfy the same sustainability criteria, unless the fishing activity falls outside the jurisdiction of a RFMO/A. Very few areas of the ocean are not under the jurisdiction of a RFMO/A, but it is difficult to determine on a global scale whether vessels are fishing for species governed by those RFMO/As at any given point in time. We therefore assume that all capacity-enhancing and ambiguous subsidies as defined by Sumaila et al. (2019) are prohibited to vessels fishing in areas beyond that Member's national jurisdiction. By default, we consider all capacity-enhancing subsidies provided to vessels spending at least 5% of their total annual effort fishing on the high seas or in the EEZs of other coastal states to be prohibited. We note that this may be an ambitious interpretation of the prohibition on subsidies to fishing in areas beyond national jurisdictions.

S&DT:
- Subsidies granted by LDCs shall be allowed, and subsidies granted by developing countries shall be allowed for fishing in their territorial seas unless they are responsible for more than 10% of annual global marine capture production. Developing Members responsible for less than 0.7% of global marine capture production will also be allowed to continue providing subsidies for fishing in their EEZs and in the high seas. As written, developing Members not meeting this criteria may also be allowed to provide subsidies for fishing in their EEZs and in the areas of competence of RFMO/As for a transition period, but this is not modeled.

Cap/Tier:
- No

INTERPRETATION #6 - Sustainability scenario + No expected IUU finding

Illegal, Unreported, and Unregulated

Assumptions:
- We assume that final determinations are made by existing RFMO/A vessel lists, and by flag and coastal Member states.
- No publicly available data exist for most flag and subsidizing Members, nor for coastal Members. The possible effects of modeling this proposal is therefore a conservative interpretation of this text. Users are free to explore a more ambitious interpretation by making their own assumptions about the expected IUU-findings.
- No proportionality or the duration of prohibition is considered.

S&DT:
- None considered.

Overfished

Assumptions:
- There is uncertainty regarding the status of many fish stocks.
- For the purposes of modeling this proposal, we assume that subsidies to fishing on stocks identified as overfished (B/Bmsy < 1) in the RAM Legacy Stock Assessment Database are prohibited. The proportion of stocks identified as overfished in the RAM Legacy Stock Assessment Database likely underestimates the actual proportion of stocks that are overfished

globally, but it is unclear whether enough evidence would exist to trigger this prohibition for stocks not included in this database.

- The proposal is ambiguous about which reference point might be used to make an overfished determination, and we therefore note that this is only one possible interpretation of this text.
- The definition for an overfished stock subsidy prohibition considered here is the more ambitious interpretation considered in the previous version of the Chair's text – Consolidated text (December 2020) (see OFOC - Negative effects (Option A)).

S&DT:

- None considered.

Overcapacity and Overfishing

Assumptions:

- We assume that all capacity-enhancing and ambiguous subsidies as defined by Sumaila et al. (2019) are prohibited for all fishing (within Members' own EEZs, on the high seas, and in the EEZs of other coastal states). We note that this is an ambitious interpretation of this proposal as no exemption is provided to demonstrate that measures are implemented to maintain stocks at a biologically sustainable level.
- The specific provisions related to high seas fishing and/or fishing in the EEZs of other coastal states are covered in the first assumption.

S&DT:

- Subsidies granted by developing and LDC Members shall be allowed for fishing within their own territorial waters. This exception might only apply with a transition period, but for the purposes of modeling we assume it to apply indefinitely.

Cap/Tier:

- No

Supplementary figures and tables

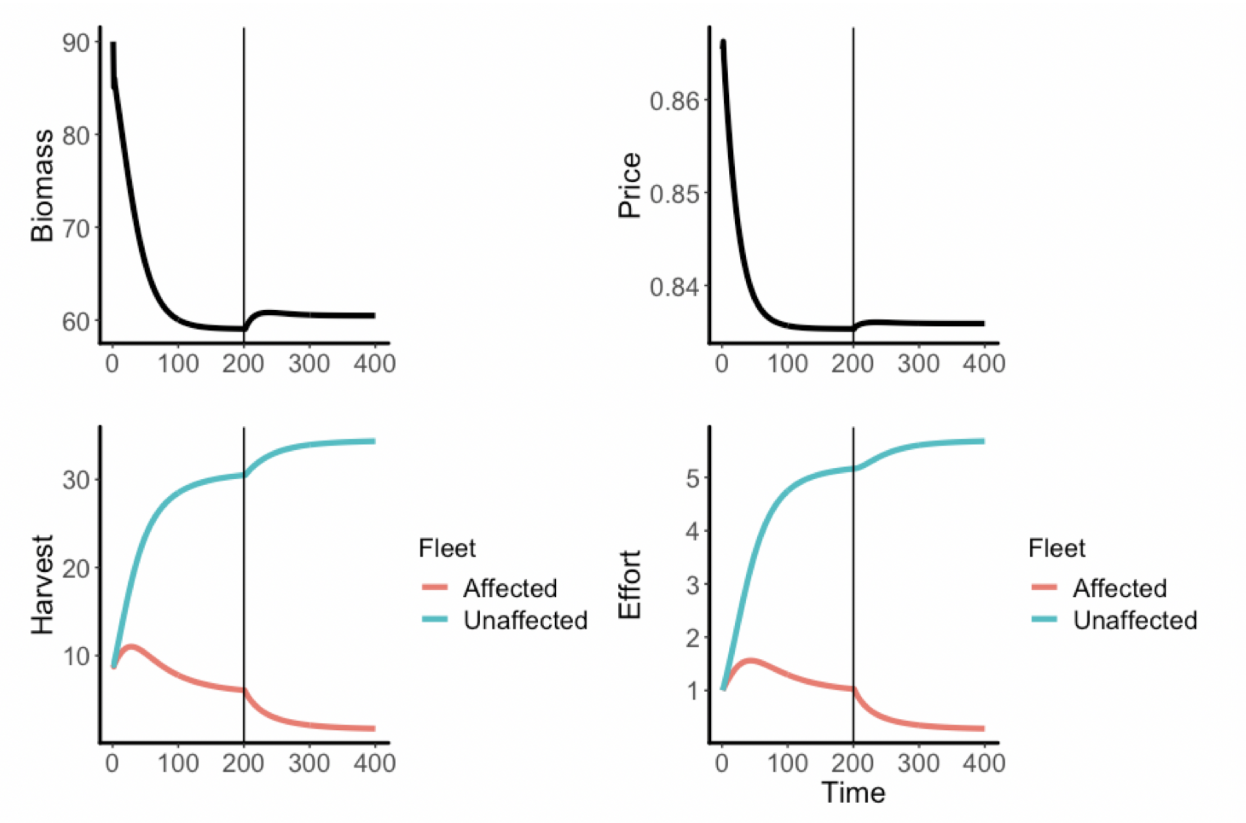

Fig A. Hypothetical illustration of a biomass- or price-driven rebound effect for a simple fishery with a single fish stock and two fleets. The vertical line at time 200 indicates the point at which subsidies are removed from the affected fleet.C

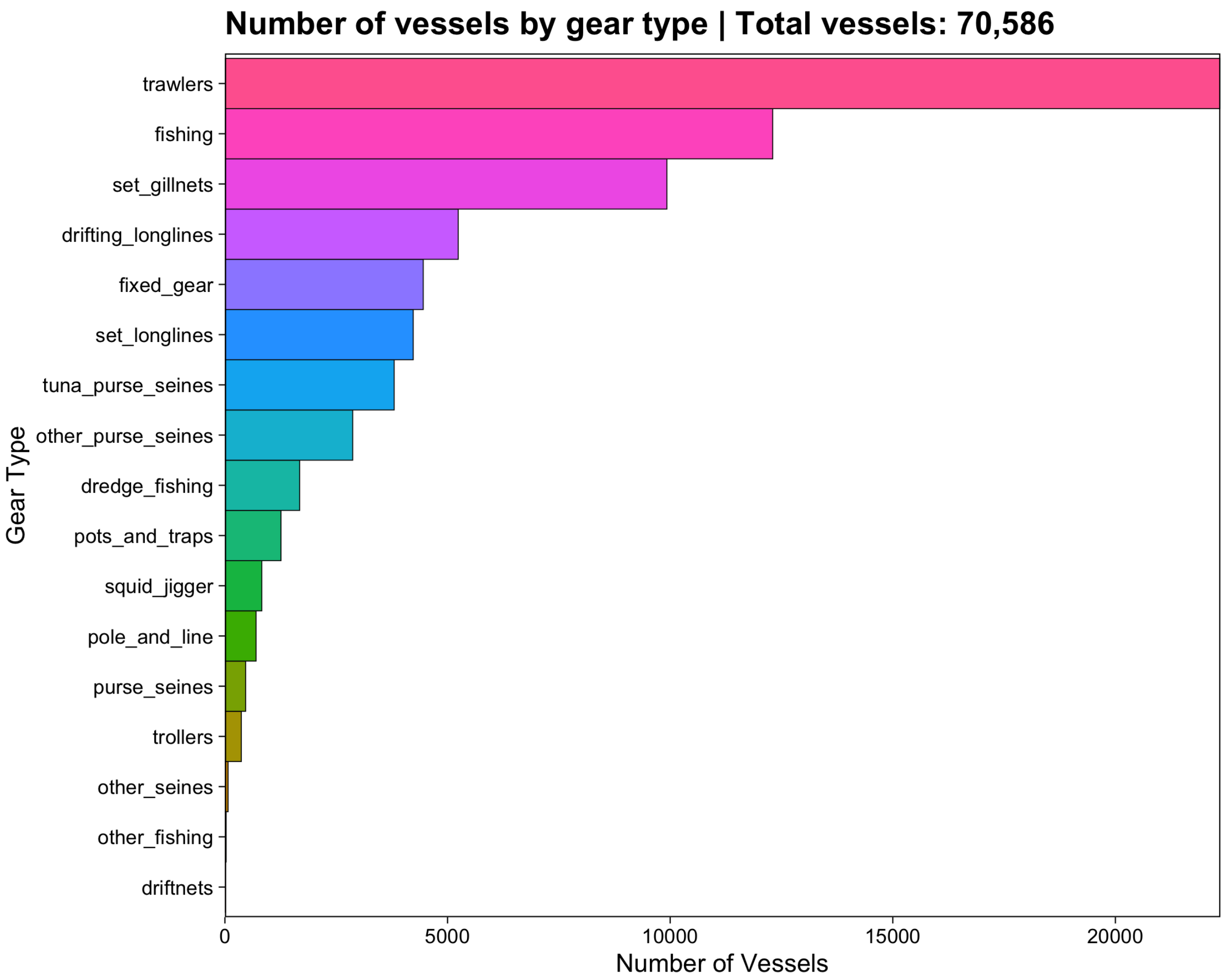

Fig B. Number of fishing vessels by gear type included in our GFW-derived global database of industrial fishing effort (2018).

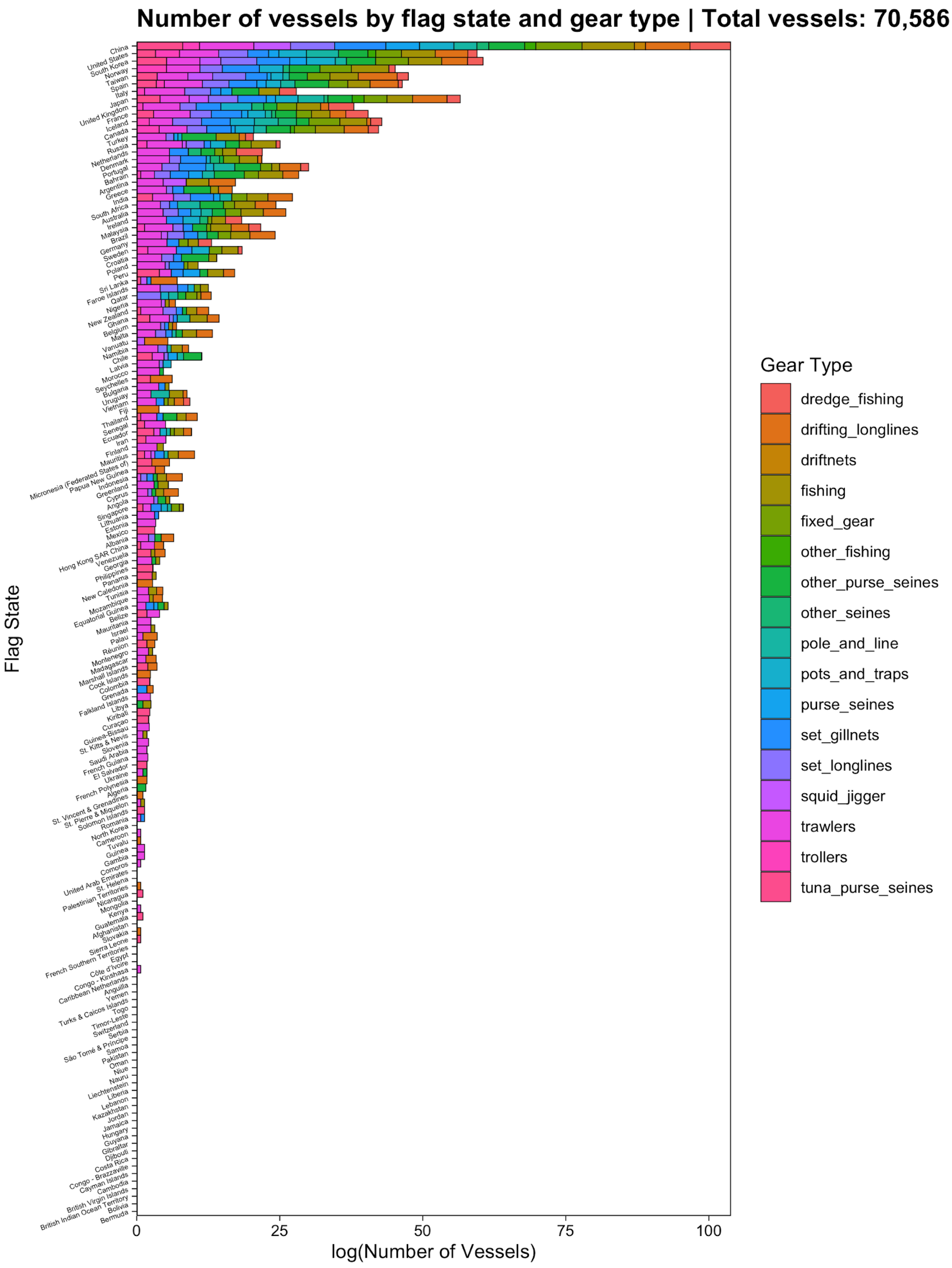

Fig C. Number of fishing vessels by flag state and gear type included in our GFW-derived global database of industrial fishing effort (2018).

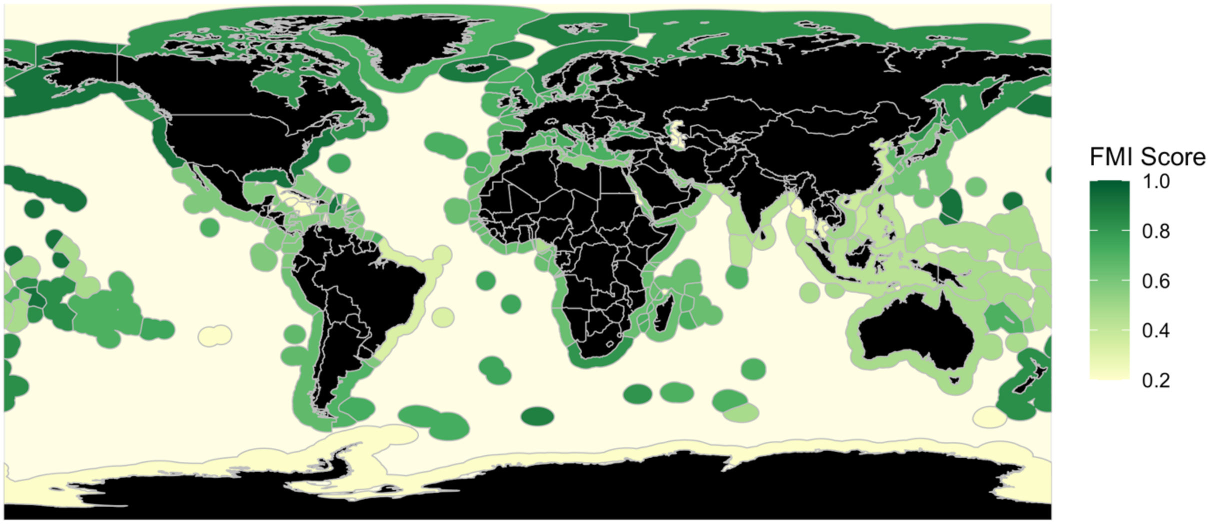

Fig D. Fisheries management indicator (FMI) scores by EEZ (and FAO statistical areas for the high seas). Extrapolated from the FMI scores in Melnychuk et al. [14]. Land boundaries depicted in this map were made using spatial data from Natural Earth (free vector and raster map data @ naturalearthdata.com). Marine boundaries depicted were made using spatial data from the Maritime Boundaries Geodatabase, version 10 [45].

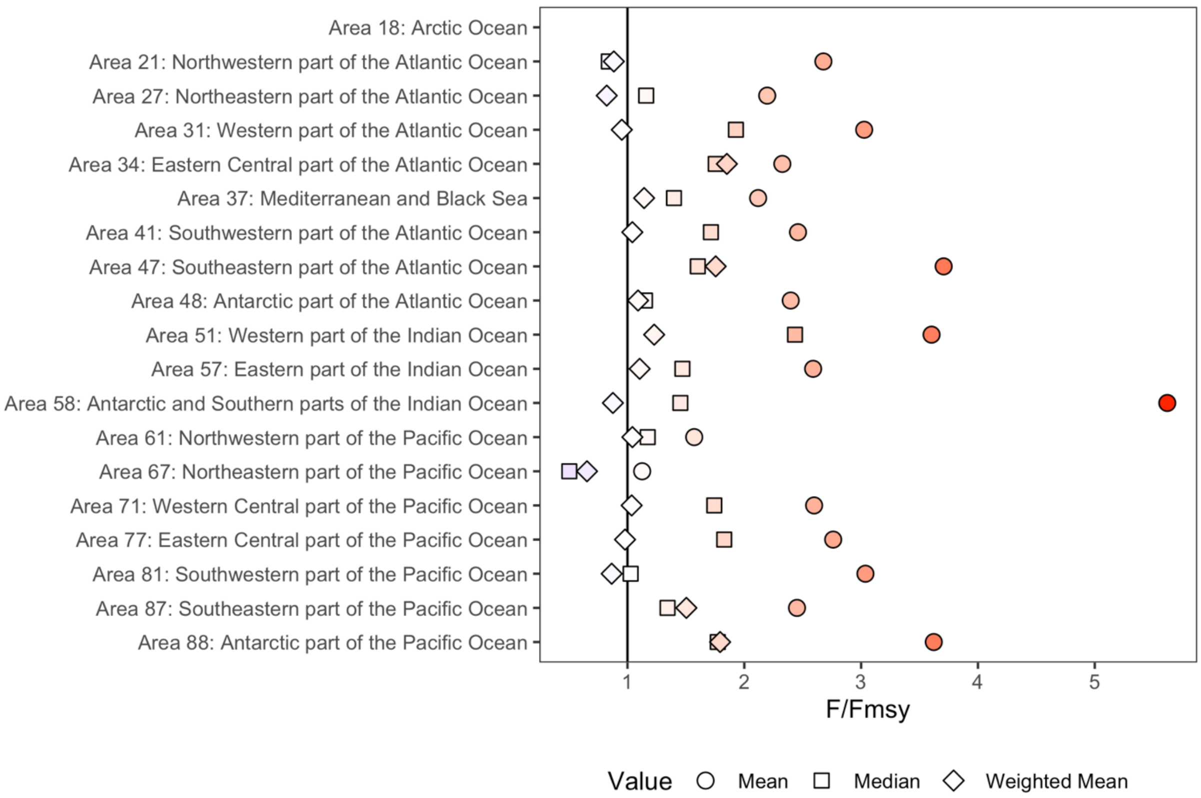

Fig E. Fishing mortality relative to that at the maximum sustainable yield (F/F_{MSY}) by FAO statistical area. All stocks assessed by Costello et al. (2016) are included. For the weighted mean calculation, the MSY of each stock was used as the weight for F/F_{MSY}.

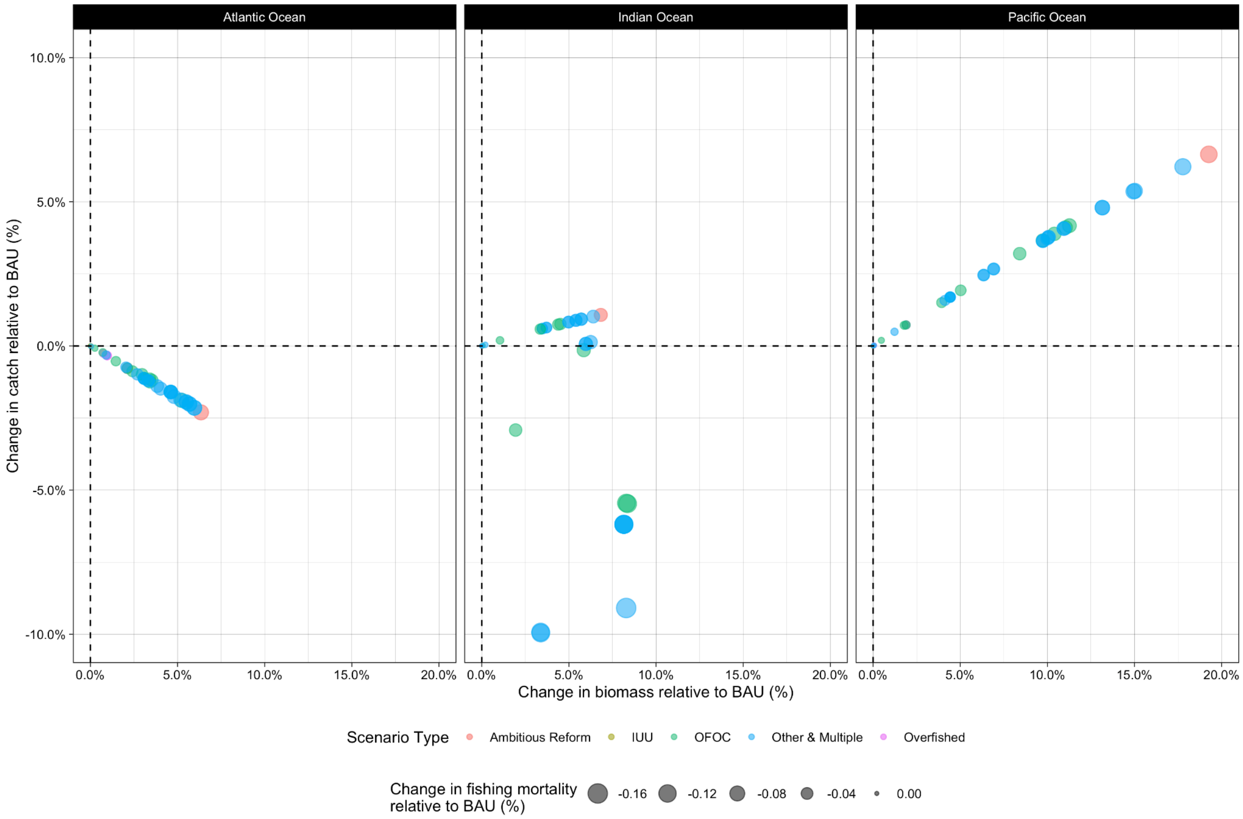

Fig F. Regional simulation model results (2050) for all pre-populated subsidy reform proposals included in SubsidyExplorer. Changes in biomass (%) are shown on the x-axis relative to a business as usual (BAU) scenario in which subsidy provisioning continues unchanged from 2018. Changes in catch (%) are shown on the y-axis relative to a BAU scenario. Changes in fishing mortality (%) relative to BAU are shown by the size of the point. The type of reform proposal is denoted by the color of the point. Proposals may pertain to reforming subsidies to illegal, unreported, and unregulated ("IUU") fishing, subsidies to fishing on overfished stocks ("Overfished"), subsidies contributing to overcapacity and overfishing ("OFOC"), or to multiple types ("Other & Multiple"). The "Ambitious Reform" scenario represents complete removal of all subsidies with the potential to be capacity-enhancing and thus represents the upper bound of effects seen from this model.

Table A. Fishery subsidy classification system based on the potential impact of a subsidy on the sustainability of the fishery resource from Sumaila et al. (2010).

Subsidy Type	Description
**A. Beneficial subsidies ("good")**	
A1. Fisheries management programs and services	These may include monitoring, control, and surveillance programs, stock assessment and resource surveys, fishery habitat enhancement programs, and stock enhancement programs.
A2. Fishery research and development (R&D)	These may include programs aimed at improving methods for fish catching and processing, as well as other programs aimed at improving fishery resources through scientific or technical developments.
A3. Marine Protected Areas (MPAs)	These may include establishment, monitoring, or enforcement of areas where commercial fishing is prohibited.
**B. Capacity-enhancing subsidies ("harmful")**	
B1. Boat construction, renewal and modernization programs	These may include lending programs below market rate geared towards fishing vessel construction, renewal and modernization (loan guarantees, restructuring, and other lending programs) as well as public support programs to adopt new and/or improve fishing technology.
B2. Fishing port construction and renovation programs	These may include provision of public funds towards fishing landing site infrastructure, port improvements for fishing fleets, harbor maintenance, jetty and landing facilities, and reduced cost or free moorage for fishing fleets.
B3. Price and marketing support, processing and storage infrastructure programs	These may include market intervention programs such as value addition and price support, or infrastructure investment programs for processing, storage, and fish auction facilities.
B4. Fishery development projects and support services	These may include programs that support fisheries enterprises development or programs that provide institutional support and services, provision of bait, and search and rescue programs.
B5. Non-fuel tax exemptions	These may include rebate and other government funded insurance support programs that have a direct impact on profits such as income tax deferral for fishers, crew insurance, duty free imports of fishing inputs, vessel insurance programs, and other economic incentive programs.
B6. Foreign access agreements	These may include explicit monetary transfers, transfers of fishing technology, or the provision of market access in another country.
B7. Fuel subsidies	This is calculated as the difference between the price per liter of fuel paid by fishers and the national price applied to fuel purchase for other uses.
**C. Ambiguous subsidies ("neutral")**	
C1. Fisher assistance programs	These include payments to fishers to stop fishing temporarily or to supplement income during bad times such as income support programs, unemployment insurance, worker adjustment programs, fishery retraining, and other direct payments to fishers.
C2. Vessel buyback programs	These may include permit or gear buybacks, or license retirements.
C3. Rural fishers' community development programs	These may include programs with an overall objective of poverty alleviation and food sufficiency.

Table B. Fishery subsidy classification system based on the implementation criteria of a transfer from the OECD's Fisheries Support Estimate (FSE) database.

Subsidy Type	Description
**0. Non-budgetary transfers to individual fishers**	
0.A. Market price support	Transfers to fishers arising from policy measures that create a gap between domestic market prices and border prices.
0.B. Fuel tax concessions	These may include programs aimed at improving methods for fish catching and processing, as well as other programs aimed at improving fishery resources through scientific or technical developments.
**1. Budgetary transfers to individual fishers**	
1.A. Transfers supporting fishing and vessel costs	Transfers to fishers based on the use of fishing inputs or factors of production--
1.A.1. Variable costs	*Transfers reducing the cost of a specific variable input or a mix of variable inputs (not including fuel tax concessions)*
1.A.2. Fixed costs	*Transfers reducing investment costs to purchase or modernize fishing vessels, gear, or any other capital asset – can be further subdivided into transfers for 1) vessel construction or purchase, 2) modernization, or 3) other transfers*
1.B. Transfers based on a fisher's income	Transfers to fishers based on their income or revenue
1.B.1. Income support	*Transfers based on income or revenue, including direct payments to vessel owners or crew.*
1.B.2. Special insurance system for fishers	*Includes measures reducing employers' social security contributions and measures providing health insurance and pension schemes with preferential conditions or rates.*
1.C. Transfers based on the reduction of productive capacity	Transfers based on the removal of vessels and licenses from a fishery, including buyouts of quota and early retirement plans
1.D. Miscellaneous transfers to fishers	Transfers to fishers that cannot be disaggregated and allocated to the other categories
**2. Transfers to the sector generally**	
2.A. Payment for access to other countries' waters	These may include government-to-government payments for the right of access, for a country's fishing fleet, to operate in another country's EEZ
2.B. Provision of infrastructure	Transfers supporting the construction, management, and access to shared facilities, including port infrastructure and activities
2.B.1. Capital expenditures	
2.B.2. Subsidized access to infrastructure	
2.C. Marketing and promotion	Transfers financing assistance to marketing and promotion of fish products
2.D. Transfers supporting fishing communities	Transfers supporting the improvement of livelihoods in fisher's communities

2.E. Education and training	Transfers financing training and education in the fishery sector
2.F. Research and development	Transfers financing research and development of activities improving production
2.G. Management of resources	Transfers financing management activities improving the productivity or the sustainability of aquatic resources
2.G.1. Management expenditures	*Transfers financing the expenditures associated with management program*
2.G.2. Stock enhancement programs	*Transfers financing stock-enhancement programs*
2.G.3. Surveillance and enforcement expenditures	*Transfers financing enforcement of management measures*
2.H. Miscellaneous transfers to general services	Transfers financing other general services that cannot be disaggregated and allocated to the other categories
**3. Cost recovery charges**	
3.A. For resource access	Charges levied on fishers to grant access to a resource, including license fees, cost of permits, and other formalities
3.B. To access infrastructure	Charges levied on fishers to grant access to infrastructure such as harbor fees and other user charges for government-provided infrastructure
3.C. For management	Charges levied on fishers to finance management, research and enforcement expenditures, or fines levied in case of infraction
3.D. Resource rent taxes and charges	Fees, taxes, or payments collected from individual fishers according to resource rents generated by fishing activities, including profit taxes
3.E. Other	Other charges levied on fishers for which there is insufficient information to allocate them to appropriate categories

Table C. Consumer Price Index (CPI) rates used to convert ex-vessel prices by year (2012 - 2018)

Year	CPI Rate
2012	229.594
2013	232.957
2014	236.736
2015	237.017
2016	240.007
2017	245.120
2018	251.107

Table D. Fixed model parameters for the global bioeconomic analysis included in SubsidyExplorer. These parameters remain constant and are not dependent on the sizes or compositions of the four fleets.

Parameter	Description	Value	Units	Source(s)	Notes
φ	Pella-Tomlinson shape parameter	0.188	---	[13, 36]	Corresponds to $B_{MSY}/K = 0.4$
MSY	Maximum sustainable yield	109,408,999	mt	[13]	MSY was adjusted to account for the 78% global coverage of the dataset from [13]
K	Biomass carrying capacity	995,621,893	mt	[36]	Global K is assumed to be 9.1 times the global MSY
g	Population growth rate	0.2747	---	---	Estimated using Equation 2 from the main text
h_0	Total harvest in the base year (2018)	84,928,268	mt	[9]	Portion of FAO reported catches assigned to industrial vessels in our database
b_0	Total biomass in the base year (2018)	212,291,690	mt	---	Estimated assuming annual biomass growth of 8 million mt/year and solving Equation 1 from the main text for the biomass that would yield observed harvests
e_0	Total fishing effort in the base year	2.42e10	kWh	GFW, see [16]	
ε	Constant elasticity of demand	-1.15	---	[13]	
p_0	Price in the base year (2018)	$1378	USD/mt	[36]	Global starting price from [36] adjusted to 2018 USD
δ	Demand constant	3.461e11	mt^2/USD	---	Estimated using Equation 7 from the main text
β	Cost non-linearity exponent	1.3	---	[13]	
η	Speed at which effort enters and exits unmanaged fisheries	0.1	kWh/USD	---	
ω	Speed at which effort enters and exists managed fisheries	0.001	kWh/USD	---	

Table E. Variable model parameters for the global bioeconomic analysis included in SubsidyExplorer. These parameters depend on the sizes and compositions of the four fleets, which is dictated by the selected subsidy reform policy. They are therefore calculated immediately prior to each model run.

Parameter	Description	Units	Sources	Notes
$h_{j,0}$	Harvest for fleet j in the base year (2018)	mt	[9]	Portion of FAO reported catches assigned to industrial vessels in the global vessel database, by fleet
$e_{j,0}$	Effort for fleet j in the base year (2018)	kWh	GFW, see [16]	
$s_{j,0}$	Rate of subsidization for fleet j in the base year (2018)	USD/kWh	[6,8]	
α_j	Cost coefficient for fleet j	---	---	Estimated using Equation 10 from the main text
q_j	Catchability for fleet j	---	---	Estimated using Equation 4 from the main text

Table F. Assignment of FAO statistical areas to regions represented in the regional bioeconomic analysis included in SubsidyExplorer.

SubsidyExplorer region	FAO statistical areas
Atlantic Ocean	21, 27, 31, 34, 37, 41, 47, 48
Indian Ocean	51, 57, 58
Pacific Ocean	18, 61, 67, 71, 77, 81, 87, 88

Table G. Regional biological model parameters used in SubsidyExplorer.

Parameter	Atlantic Ocean	Indian Ocean	Pacific Ocean	Units	Source(s)
φ	0.188	0.188	0.188	---	[13, 36]
MSY	27,193,862	8,403,365	62,607,176	mt	[13]
K	247,464,144	76,470,622	569,725,302	mt	[36]
g	0.2747	0.2747	0.2747	---	---
h_0	24,270,674	10,215,324	50,442,270	mt	[9]
b_0	111,815,482	30,583,660	121,107,224	mt	---
e_0	1.08e10	1.47e9	1.20e10	kWh	GFW, see [16]
ε	-1.15	-1.15	-1.15	---	[13]
p_0	$1378	$1378	$1378	USD/mt	[36]
δ	9.891e10	4.163e10	2.055e11	mt^2/USD	---
β	1.3	1.3	1.3	---	[13]
η	0.1	0.1	0.1	kWh/USD	---
ω	0.001	0.001	0.001	kWh/USD	---

Table H. Complementarities between subsidy types defined by Sumaila et al. [6] and certain fisheries support estimate (FSE) types for which the relative effects were assessed by the OECD.

OECD support type	Description	Complementary subsidy type(s) defined by Sumaila et al. [6]
Inputs (variable costs)	Support based on the variable costs of fishing, such as payments contingent upon the purchase of gear, bait or ice, or the use of port facilities	Fishing port construction and renovation programs (B2); Fishery development projects and support services (B4); Foreign access agreements (B6)
Outputs	Support based on the price of fish (including tariffs, market interventions and consumer subsidies)	Price and marketing support, processing and storage infrastructure programs (B3)
Fuel	Payments based upon the quantity of fuel used	Fuel subsidies (B7)
Income	Payments based on fishers' income (e.g., employment insurance, disaster payments, wage subsidies, special income tax concessions)	Non-fuel tax exemptions (B5); Fisher assistance programs (C1); Rural fishers' community development programs (C3)
Capital	Payments based on fishers' own capital (e.g., concessional loans, special tax treatment on investment or returns on investment other than for capital in fishing vessels, upskilling, marketing training and assistance)	Non-fuel tax exemptions (B5); Fisher assistance programs (C1); Rural fishers' community development programs (C3)
Vessels	Payments based on the construction, modernization, or scrapping of vessels	Boat construction, renewal and modernization programs (B1); Vessel buyback programs (C2)

Table I. Relative performance of six fishery support types on fishing effort, fleet capacity, fishers' income, and stock size from the OECD. Impacts were assessed under two management scenarios: open-access and a total-allowable catch (TAC). Adapted from Fig. 3.17 from Martini & Innes [32].

Support category	Open-access				TAC			
	Effort	Capacity	Income	Stock	Effort	Capacity	Income	Stock
Inputs	**1.00**	0.02	0.28	1.00	1.00	-0.17	0.78	1.00
Outputs	**0.87**	0.27	0.59	0.87	0.89	0.11	0.91	0.89
Fuel	**0.84**	0.12	0.11	0.84	0.96	-0.15	0.57	0.96
Income	**0.76**	0.37	0.85	0.76	0.76	0.29	1.00	0.76
Capital	**0.57**	0.04	0.98	0.57	0.53	-0.08	0.90	0.53
Vessels	**0.55**	1.00	1.00	0.55	0.62	1.00	0.92	0.62

Table J. Global simulation model results (2050) for all pre-populated subsidy-reform proposals included in SubsidyExplorer.

Proposal	Category	Change in 2050 relative to BAU (%)			
		Biomass	Catch	Fishing Mortality	Revenue
All subsidies	Ambitious Reform	12.49	3.494	-8.265	0.432
IUU (Option A)	IUU	0.006	-0.001	-0.005	0
IUU (Option B)	IUU	0.006	-0.001	-0.005	0
Facilitator's working text - IUU	IUU	0.004	0	-0.003	0
IUU (Option C)	IUU	0.006	-0.001	-0.005	0
CAP - De minimis	OFOC	0.341	0.097	-0.214	0.012
OFOC - Negative effects (Option A)	OFOC	1.819	0.219	-1.136	0.028
CAP - Optional criteria	OFOC	1.249	0.389	-0.954	0.05
OFOC - ABNJ	OFOC	4.841	0.342	-5.573	0.018
OFOC - List approach	OFOC	4.793	1.601	-4.121	0.183
CAP - Tiers	OFOC	6.195	2.007	-4.182	0.252
OFOC - Vessel characteristics	OFOC	8.105	1.983	-6.259	0.245
OFOC - Prohibition + management	OFOC	1.819	0.219	-1.136	0.028
OFOC - Negative effects + ABNJ	OFOC	3.93	0.191	-4.897	-0.001
CAP - Formula	OFOC	7.166	2.235	-4.964	0.281
CAP - Formula (revised)	OFOC	6.797	2.114	-4.743	0.266
Overfished - Negative effects (Option A)	Overfished	0.445	-0.087	-0.305	-0.011
Overfished - Negative effects + rebuttable (Option B)	Overfished	0.344	-0.07	-0.237	-0.009
Facilitator's text - Overfished [Objective definition]	Overfished	1.819	0.219	-1.136	0.028
Facilitator's text - Overfished [Relevant authorities]	Overfished	0.445	-0.087	-0.305	-0.011
Disputed waters	Other & Multiple	0.018	-0.004	-0.013	-0.001
Comprehensive text proposal	Other & Multiple	11.072	3.387	-7.395	0.42
S&DT	Other & Multiple	6.892	1.801	-5.501	0.224
Chair's text - Consolidated text (Dec 2020) [Objective definition]	Other & Multiple	4.429	0.176	-5.309	-0.008
Chair's text - Consolidated text (Dec 2020) [Relevant authorities]	Other & Multiple	4.422	0.185	-5.3	-0.007
Chair's text - Consolidated text (Dec 2020) [Objective definition + CAP - Optional criteria]	Other & Multiple	5.626	1.225	-4.079	0.155
Chair's text - Consolidated text (Dec 2020) [Relevant authorities + CAP - Optional criteria]	Other & Multiple	5.611	1.222	-4.07	0.155
Chair's text - Consolidated text (Dec 2020) [Objective definition + CAP - Tiers]	Other & Multiple	7.617	1.819	-5.427	0.228
Chair's text - Consolidated text (Dec 2020) [Relevant authorities + CAP - Tiers]	Other & Multiple	7.606	1.815	-5.421	0.228
Chair's text - Consolidated text (Dec 2020) [Objective definition + CAP - De minimis]	Other & Multiple	5.334	1.093	-3.877	0.138
Chair's text - Consolidated text (Dec 2020) [Relevant authorities + CAP - De minimis]	Other & Multiple	5.319	1.089	-3.868	0.138
Chair's text - Consolidated text (Dec 2020) [Objective definition + CAP - Formula]	Other & Multiple	8.175	1.988	-5.875	0.249
Chair's text - Consolidated text (Dec 2020) [Relevant authorities + CAP - Formula]	Other & Multiple	8.171	1.988	-5.874	0.249
Artisanal - Exemption	Other & Multiple	4.415	0.182	-5.294	-0.007
Chair's text - June 2021 [Full exemption + S&DT for all developing countries]	Other & Multiple	1.491	0.087	-0.988	0.011
Chair's text - June 2021 [Full exemption]	Other & Multiple	4.422	0.185	-5.3	-0.007
Chair's text - June 2021 [Managed exemption]	Other & Multiple	6.803	1.826	-5.435	0.228
Chair's text - June 2021 [Sustainability scenario]	Other & Multiple	9.31	2.39	-6.394	0.298
Chair's text - Nov 2021 [Full exemption]	Other & Multiple	0.422	-0.081	-0.29	-0.01
Chair's text - Nov 2021 [Managed exemption]	Other & Multiple	8.82	2.26	-8.701	0.211
Chair's text - Nov 2021 [Sustainability scenario]	Other & Multiple	9.31	2.39	-6.394	0.298
Chair's text - Nov 2021 [Full exemption for domestic only]	Other & Multiple	4.203	-0.041	-5.344	-0.056
Chair's text - Nov 2021 [Managed exemption for domestic only]	Other & Multiple	8.869	2.264	-8.742	0.211
Chair's text - Nov 2021 [Sustainability scenario + No expected IUU finding]	Other & Multiple	7.539	1.745	-5.956	0.217

Table K. Regional (Atlantic Ocean) simulation model results (2050) for all pre-populated subsidy-reform proposals included in SubsidyExplorer.

Proposal	Category	Change in 2050 relative to BAU (%)			
		Biomass	Catch	Fishing Mortality	Revenue
All subsidies	Ambitious Reform	6.343	-2.304	-8.132	-0.304
IUU (Option A)	IUU	0.013	-0.004	-0.016	0
IUU (Option B)	IUU	0.013	-0.004	-0.016	0
Facilitator's working text - IUU	IUU	0.008	-0.002	-0.01	0
IUU (Option C)	IUU	0.013	-0.004	-0.016	0
CAP - De minimis	OFOC	0.254	-0.079	-0.332	-0.01
OFOC - Negative effects (Option A)	OFOC	2.117	-0.771	-2.828	-0.101
CAP - Optional criteria	OFOC	0.712	-0.228	-0.934	-0.03
OFOC - ABNJ	OFOC	3.417	-1.247	-4.51	-0.164
OFOC - List approach	OFOC	1.461	-0.528	-1.961	-0.069
CAP - Tiers	OFOC	2.963	-0.986	-3.835	-0.129
OFOC - Vessel characteristics	OFOC	5.255	-1.892	-6.789	-0.249
OFOC - Prohibition + management	OFOC	2.117	-0.771	-2.828	-0.101
OFOC - Negative effects + ABNJ	OFOC	2.41	-0.878	-3.21	-0.115
CAP - Formula	OFOC	3.524	-1.192	-4.556	-0.156
CAP - Formula (revised)	OFOC	3.402	-1.147	-4.4	-0.15
Overfished - Negative effects (Option A)	Overfished	0.958	-0.333	-1.279	-0.044
Overfished - Negative effects + rebuttable (Option B)	Overfished	0.748	-0.262	-1.002	-0.034
Facilitator's text - Overfished [Objective definition]	Overfished	2.117	-0.771	-2.828	-0.101
Facilitator's text - Overfished [Relevant authorities]	Overfished	0.958	-0.333	-1.279	-0.044
Disputed waters	Other & Multiple	0.037	-0.017	-0.054	-0.002
Comprehensive text proposal	Other & Multiple	4.8	-1.757	-6.257	-0.231
S&DT	Other & Multiple	4.031	-1.482	-5.299	-0.195
Chair's text - Consolidated text (Dec 2020) [Objective definition]	Other & Multiple	3.106	-1.136	-4.115	-0.149
Chair's text - Consolidated text (Dec 2020) [Relevant authorities]	Other & Multiple	3.076	-1.123	-4.074	-0.147
Chair's text - Consolidated text (Dec 2020) [Objective definition + CAP - Optional criteria]	Other & Multiple	4.615	-1.6	-5.941	-0.21
Chair's text - Consolidated text (Dec 2020) [Relevant authorities + CAP - Optional criteria]	Other & Multiple	4.606	-1.596	-5.929	-0.21
Chair's text - Consolidated text (Dec 2020) [Objective definition + CAP - Tiers]	Other & Multiple	5.502	-1.946	-7.059	-0.256
Chair's text - Consolidated text (Dec 2020) [Relevant authorities + CAP - Tiers]	Other & Multiple	5.501	-1.945	-7.058	-0.256
Chair's text - Consolidated text (Dec 2020) [Objective definition + CAP - De minimis]	Other & Multiple	4.616	-1.6	-5.942	-0.21
Chair's text - Consolidated text (Dec 2020) [Relevant authorities + CAP - De minimis]	Other & Multiple	4.606	-1.596	-5.929	-0.21
Chair's text - Consolidated text (Dec 2020) [Objective definition + CAP - Formula]	Other & Multiple	5.691	-2.014	-7.29	-0.265
Chair's text - Consolidated text (Dec 2020) [Relevant authorities + CAP - Formula]	Other & Multiple	5.683	-2.015	-7.284	-0.265
Artisanal - Exemption	Other & Multiple	3.076	-1.123	-4.073	-0.147
Chair's text - June 2021 [Full exemption + S&DT for all developing countries]	Other & Multiple	2.041	-0.736	-2.722	-0.096
Chair's text - June 2021 [Full exemption]	Other & Multiple	3.076	-1.123	-4.074	-0.147
Chair's text - June 2021 [Managed exemption]	Other & Multiple	3.833	-1.392	-5.033	-0.183
Chair's text - June 2021 [Sustainability scenario]	Other & Multiple	5.965	-2.147	-7.656	-0.283
Chair's text - Nov 2021 [Full exemption]	Other & Multiple	0.903	-0.315	-1.207	-0.041
Chair's text - Nov 2021 [Managed exemption]	Other & Multiple	3.303	-1.198	-4.357	-0.157
Chair's text - Nov 2021 [Sustainability scenario]	Other & Multiple	5.965	-2.147	-7.656	-0.283
Chair's text - Nov 2021 [Full exemption for domestic only]	Other & Multiple	2.687	-0.983	-3.574	-0.129
Chair's text - Nov 2021 [Managed exemption for domestic only]	Other & Multiple	3.337	-1.215	-4.405	-0.159
Chair's text - Nov 2021 [Sustainability scenario + No expected IUU finding]	Other & Multiple	5.187	-1.874	-6.713	-0.246

Table L. Regional (Indian Ocean) simulation model results (2050) for all pre-populated subsidy-reform proposals included in SubsidyExplorer.

Proposal	Category	Change in 2050 relative to BAU (%)			
		Biomass	Catch	Fishing Mortality	Revenue
All subsidies	Ambitious Reform	6.832	1.077	-5.387	0.14
IUU (Option A)	IUU	0.001	0	-0.001	0
IUU (Option B)	IUU	0.001	0	-0.001	0
Facilitator's working text - IUU	IUU	0.001	0	-0.001	0
IUU (Option C)	IUU	0.001	0	-0.001	0
CAP - De minimis	OFOC	0.061	0.011	-0.05	0.001
OFOC - Negative effects (Option A)	OFOC	0.001	0	-0.001	0
CAP - Optional criteria	OFOC	1.045	0.193	-0.844	0.025
OFOC - ABNJ	OFOC	8.369	-5.473	-12.773	-0.731
OFOC - List approach	OFOC	1.943	-2.918	-4.768	-0.386
CAP - Tiers	OFOC	3.357	0.584	-2.682	0.076
OFOC - Vessel characteristics	OFOC	5.852	-0.147	-5.667	-0.019
OFOC - Prohibition + management	OFOC	0.001	0	-0.001	0
OFOC - Negative effects + ABNJ	OFOC	8.301	-5.45	-12.698	-0.728
CAP - Formula	OFOC	4.513	0.76	-3.591	0.099
CAP - Formula (revised)	OFOC	4.387	0.742	-3.492	0.096
Overfished - Negative effects (Option A)	Overfished	0	0	0	0
Overfished - Negative effects + rebuttable (Option B)	Overfished	0	0	0	0
Facilitator's text - Overfished [Objective definition]	Overfished	0.001	0	-0.001	0
Facilitator's text - Overfished [Relevant authorities]	Overfished	0	0	0	0
Disputed waters	Other & Multiple	0	0	0	0
Comprehensive text proposal	Other & Multiple	6.397	1.018	-5.056	0.132
S&DT	Other & Multiple	5.971	0.071	-5.568	0.009
Chair's text - Consolidated text (Dec 2020) [Objective definition]	Other & Multiple	8.155	-6.19	-13.263	-0.83
Chair's text - Consolidated text (Dec 2020) [Relevant authorities]	Other & Multiple	8.155	-6.19	-13.263	-0.83
Chair's text - Consolidated text (Dec 2020) [Objective definition + CAP - Optional criteria]	Other & Multiple	3.711	0.638	-2.963	0.083
Chair's text - Consolidated text (Dec 2020) [Relevant authorities + CAP - Optional criteria]	Other & Multiple	3.71	0.638	-2.962	0.083
Chair's text - Consolidated text (Dec 2020) [Objective definition + CAP - Tiers]	Other & Multiple	4.984	0.828	-3.958	0.108
Chair's text - Consolidated text (Dec 2020) [Relevant authorities + CAP - Tiers]	Other & Multiple	4.983	0.828	-3.957	0.108
Chair's text - Consolidated text (Dec 2020) [Objective definition + CAP - De minimis]	Other & Multiple	3.474	0.601	-2.776	0.078
Chair's text - Consolidated text (Dec 2020) [Relevant authorities + CAP - De minimis]	Other & Multiple	3.473	0.601	-2.775	0.078
Chair's text - Consolidated text (Dec 2020) [Objective definition + CAP - Formula]	Other & Multiple	5.708	0.929	-4.521	0.121
Chair's text - Consolidated text (Dec 2020) [Relevant authorities + CAP - Formula]	Other & Multiple	5.708	0.929	-4.521	0.121
Artisanal - Exemption	Other & Multiple	8.155	-6.19	-13.263	-0.83
Chair's text - June 2021 [Full exemption + S&DT for all developing countries]	Other & Multiple	0.202	0.037	-0.164	0.005
Chair's text - June 2021 [Full exemption]	Other & Multiple	8.155	-6.19	-13.263	-0.83
Chair's text - June 2021 [Managed exemption]	Other & Multiple	5.973	0.072	-5.569	0.009
Chair's text - June 2021 [Sustainability scenario]	Other & Multiple	5.403	0.887	-4.284	0.115
Chair's text - Nov 2021 [Full exemption]	Other & Multiple	0.001	0	-0.001	0
Chair's text - Nov 2021 [Managed exemption]	Other & Multiple	3.379	-9.926	-12.871	-1.354
Chair's text - Nov 2021 [Sustainability scenario]	Other & Multiple	5.403	0.887	-4.284	0.115
Chair's text - Nov 2021 [Full exemption for domestic only]	Other & Multiple	8.288	-9.091	-16.049	-1.236
Chair's text - Nov 2021 [Managed exemption for domestic only]	Other & Multiple	3.385	-9.958	-12.905	-1.359
Chair's text - Nov 2021 [Sustainability scenario + No expected IUU finding]	Other & Multiple	6.249	0.132	-5.757	0.017

Table M. Regional (Pacific Ocean) simulation model results (2050) for all pre-populated subsidy-reform proposals included in SubsidyExplorer.

Proposal	Category	Change in 2050 relative to BAU (%)			
		Biomass	Catch	Fishing Mortality	Revenue
All subsidies	Ambitious Reform	19.263	6.646	-10.579	0.843
IUU (Option A)	IUU	0.002	0.001	-0.001	0
IUU (Option B)	IUU	0.002	0.001	-0.001	0
Facilitator's working text - IUU	IUU	0.002	0.001	-0.001	0
IUU (Option C)	IUU	0.002	0.001	-0.001	0
CAP - De minimis	OFOC	0.476	0.194	-0.28	0.025
OFOC - Negative effects (Option A)	OFOC	1.892	0.728	-1.142	0.095
CAP - Optional criteria	OFOC	1.785	0.715	-1.051	0.093
OFOC - ABNJ	OFOC	5.027	1.929	-2.95	0.25
OFOC - List approach	OFOC	8.414	3.202	-4.808	0.412
CAP - Tiers	OFOC	9.731	3.66	-5.533	0.47
OFOC - Vessel characteristics	OFOC	11.264	4.173	-6.373	0.535
OFOC - Prohibition + management	OFOC	1.892	0.728	-1.142	0.095
OFOC - Negative effects + ABNJ	OFOC	3.933	1.502	-2.339	0.195
CAP - Formula	OFOC	11.044	4.106	-6.249	0.526
CAP - Formula (revised)	OFOC	10.4	3.888	-5.898	0.499
Overfished - Negative effects (Option A)	Overfished	0.056	0.019	-0.037	0.002
Overfished - Negative effects + rebuttable (Option B)	Overfished	0.036	0.012	-0.024	0.002
Facilitator's text - Overfished [Objective definition]	Overfished	1.892	0.728	-1.142	0.095
Facilitator's text - Overfished [Relevant authorities]	Overfished	0.056	0.019	-0.037	0.002
Disputed waters	Other & Multiple	0.003	0.001	-0.002	0
Comprehensive text proposal	Other & Multiple	17.774	6.217	-9.813	0.79
S&DT	Other & Multiple	9.746	3.644	-5.56	0.468
Chair's text - Consolidated text (Dec 2020) [Objective definition]	Other & Multiple	4.415	1.688	-2.612	0.219
Chair's text - Consolidated text (Dec 2020) [Relevant authorities]	Other & Multiple	4.429	1.697	-2.616	0.22
Chair's text - Consolidated text (Dec 2020) [Objective definition + CAP - Optional criteria]	Other & Multiple	6.93	2.673	-3.981	0.345
Chair's text - Consolidated text (Dec 2020) [Relevant authorities + CAP - Optional criteria]	Other & Multiple	6.907	2.665	-3.968	0.344
Chair's text - Consolidated text (Dec 2020) [Objective definition + CAP - Tiers]	Other & Multiple	10.08	3.779	-5.724	0.485
Chair's text - Consolidated text (Dec 2020) [Relevant authorities + CAP - Tiers]	Other & Multiple	10.058	3.772	-5.711	0.484
Chair's text - Consolidated text (Dec 2020) [Objective definition + CAP - De minimis]	Other & Multiple	6.356	2.463	-3.661	0.318
Chair's text - Consolidated text (Dec 2020) [Relevant authorities + CAP - De minimis]	Other & Multiple	6.334	2.455	-3.648	0.317
Chair's text - Consolidated text (Dec 2020) [Objective definition + CAP - Formula]	Other & Multiple	10.947	4.073	-6.196	0.522
Chair's text - Consolidated text (Dec 2020) [Relevant authorities + CAP - Formula]	Other & Multiple	10.947	4.073	-6.196	0.522
Artisanal - Exemption	Other & Multiple	4.412	1.692	-2.605	0.219
Chair's text - June 2021 [Full exemption + S&DT for all developing countries]	Other & Multiple	1.23	0.491	-0.73	0.064
Chair's text - June 2021 [Full exemption]	Other & Multiple	4.429	1.697	-2.616	0.22
Chair's text - June 2021 [Managed exemption]	Other & Multiple	9.737	3.643	-5.553	0.468
Chair's text - June 2021 [Sustainability scenario]	Other & Multiple	13.155	4.798	-7.385	0.613
Chair's text - Nov 2021 [Full exemption]	Other & Multiple	0.057	0.019	-0.038	0.003
Chair's text - Nov 2021 [Managed exemption]	Other & Multiple	14.943	5.36	-8.337	0.683
Chair's text - Nov 2021 [Sustainability scenario]	Other & Multiple	13.155	4.798	-7.385	0.613
Chair's text - Nov 2021 [Full exemption for domestic only]	Other & Multiple	4.127	1.58	-2.446	0.205
Chair's text - Nov 2021 [Managed exemption for domestic only]	Other & Multiple	15.015	5.378	-8.379	0.686
Chair's text - Nov 2021 [Sustainability scenario + No expected IUU finding]	Other & Multiple	10.003	3.733	-5.699	0.479

SI references

1. R Core Team, *R: A language and environment for statistical computing* (2019).
2. W. Chang, J. Cheng, J. Allaire, Y. Xie, J. McPherson, *shiny: Web Application Framework for R. R package version 1.4.0.* (2019).
3. World Bank, "World Development Indicators" (2020).
4. FAO, "FAO Yearbook of Fishery and Aquaculture Statistics 2017" (2019).
5. L. C. L. Teh, U. R. Sumaila, Contribution of marine fisheries to worldwide employment. *Fish Fish.* **14**, 77–88 (2011).
6. U. R. Sumaila, *et al.*, Updated estimates and analysis of global fisheries subsidies. *Mar. Policy* **109**, 103695 (2019).
7. OECD, Fisheries: Fisheries support estimates. *Fish. Aquac. Stat. OECD Agric. Stat. Database* (2019) https://doi.org/10.1787/ade64fdc-en (March 10, 2019).
8. A. Schuhbauer, R. Chuenpagdee, W. W. L. Cheung, K. Greer, U. R. Sumaila, How subsidies affect the economic viability of small-scale fisheries. *Mar. Policy* **82**, 114–121 (2017).
9. FAO, Global Capture Production Dataset (2020).
10. M. C. Melnychuk, T. Clavelle, B. Owashi, K. Strauss, Reconstruction of global ex-vessel prices of fished species. *ICES J. Mar. Sci.* **74**, 121–133 (2017).
11. D. Ricard, C. Minto, O. P. Jensen, J. K. Baum, Examining the knowledge base and status of commercially exploited marine species with the RAM Legacy Stock Assessment Database. *Fish Fish.* **13**, 380–398 (2012).
12. RAM Legacy Stock Assessment Database, Version 4.491-assessment only. Released 2020-02-20 (2020) https:/doi.org/10.5281/zenodo.3676087 (March 30, 2020).
13. C. Costello, *et al.*, Global fishery prospects under contrasting management regimes. *Proc. Natl. Acad. Sci.* **113**, 5125–5129 (2016).
14. M. C. Melnychuk, E. Peterson, M. Elliott, R. Hilborn, Fisheries management impacts on target species status. *Proc. Natl. Acad. Sci.* **114**, 178–183 (2017).
15. Trygg Mat Tracking, "Combined IUU Fishing Vessel List" (2020).
16. D. A. Kroodsma, *et al.*, Tracking the global footprint of fisheries. *Science* **359**, 904–908 (2018).
17. Global Fishing Watch, Global Fishing Watch: Sustainability through Transparency (2020) (January 21, 2020).
18. V. Arel-Bundock, *WDI: World Development Indicators (World Bank). R package version 2.6.0.* (2019).
19. U. R. Sumaila, V. Lam, F. Le Manach, W. Swartz, D. Pauly, Global fisheries subsidies: An updated estimate. *Mar. Policy* **69**, 189–193 (2016).
20. U. R. Sumaila, *et al.*, A bottom-up re-estimation of global fisheries subsidies. *J. Bioeconomics* **12**, 201–225 (2010).
21. R. Martini, "The Fisheries Support Estimate (FSE) Manual" (2016).
22. C. Costello, *et al.*, Status and Solutions for the World's Unassessed Fisheries. *Science* **338**, 517–520 (2012).
23. World Trade Organization, WTO members prepare to firm up legal text for fisheries subsidies agreement (2020).
24. H. Wickham, *tidyverse: Easily Install and Load the "Tidyverse". R package version 1.2.1.* (2017).
25. S. Firke, *janitor: Simple Tools for Examining and Cleaning Dirty Data. R package version 1.2.0.* (2019).
26. V. Arel-Bundock, N. Enevoldsen, C. Yetman, countrycode: An R package to convert country names and country codes. *J. Open Source Softw.* **3**, 848 (2018).
27. H. Wickham, J. Bryan, *readxl: Read Excel Files. R package version 1.3.1.* (2019).
28. U.S. Bureau of Labor Statistics, Consumer Price Index (2020).
29. A. Schuhbauer, A. M. Cisneros-Montemayor, R. Chuenpagdee, U. R. Sumaila, Assessing the

economic viability of small-scale fisheries: an example from Mexico. *Mar. Ecol. Prog. Ser.* **617–618**, 365–376 (2019).
30. H. Wickham, J. Bryan, *bigrquery: An Interface to Google's "BigQuery" "API". R package version 1.2.0.*
31. E. Sala, *et al.*, The economics of fishing the high seas. *Sci. Adv.* **4** (2018).
32. R. Martini, J. Innes, Relative Effects of Fisheries Support Policies (2018) https:/doi.org/https://doi.org/10.1787/bd9b0dc3-en (February 6, 2020).
33. OECD, "Support to fisheries: Levels and impacts" (2017) https:/doi.org/10.1787/00287855-en (July 18, 2018).
34. Y. Sakai, Subsidies, Fisheries Management, and Stock Depletion. *Land Econ.* **93**, 165–178 (2017).
35. World Bank, FAO, "The Sunken Billions: The Economic Justification for Fisheries Reform" (2009).
36. World Bank, "The Sunken Billions Revisited: Progress and Challenges in Global Marine Fisheries" (2017).
37. R. B. Cabral, *et al.*, Rapid and lasting gains from solving illegal fishing. *Nat. Ecol. Evol.* **2**, 650–658 (2018).
38. A. Schuhbauer, D. J. Skerritt, N. Ebrahim, F. Le Manach, U. R. Sumaila, The Global Fisheries Subsidies Divide Between Small- and Large-Scale Fisheries. *Front. Mar. Sci.* **7** (2020), doi:10.3389/fmars.2020.539214.
39. K. Greer, D. Zeller, J. Woroniak, A. Coulter, M. Winchester, M. L. D. Palomares, D. Pauly, Global trends in carbon dioxide (CO2) emissions from fuel combustion in marine fisheries from 1950 to 2016. *Marine Policy*. **107**, 103382 (2019).
40. R. W. R. Parker, J. L. Blanchard, C. Gardner, B. S. Green, K. Hartmann, P. H. Tyedmers, R. A. Watson, Fuel use and greenhouse gas emissions of world fisheries. *Nature Clim Change*. **8**, 333–337 (2018).
41. R. Hilborn, R. O. Amoroso, C. M. Anderson, J. K. Baum, T. A. Branch, C. Costello, C. L. de Moor, A. Faraj, D. Hively, O. P. Jensen, H. Kurota, L. R. Little, P. Mace, T. McClanahan, M. C. Melnychuk, C. Minto, G. C. Osio, A. M. Parma, M. Pons, S. Segurado, C. S. Szuwalski, J. R. Wilson, Y. Ye, Effective fisheries management instrumental in improving fish stock status. *PNAS* (2020), doi:10.1073/pnas.1909726116.
42. T. Chesnokova, S. McWhinnie, International Fisheries Access Agreements and Trade. *Environ Resource Econ*. **74**, 1207–1238 (2019).
43. J. C. Villaseñor-Derbez, J. Lynham, C. Costello, Environmental market design for large-scale marine conservation. *Nature Sustainability*. **3**, 234–240 (2020).
44. C. A. Grainger, C. Costello, Distributional Effects of the Transition to Property Rights for a Common-Pool Resource. *Marine Resource Economics*. **31**, 1–26 (2016).
46. Flanders Marine Institute, Maritime Boundaries Geodatabase, version 10 (2018). Available from: https://doi.org/10.14284/319.
